# Single-nucleus multiomics of murine gonads reveals transcriptional regulatory network underlying supporting lineage differentiation

**DOI:** 10.1126/sciadv.aea7403

**Published:** 2026-01-02

**Authors:** Yu-Ying Chen, Karina Rodriguez, Adriana K. Alexander, Xin Xu, Brian Papas, Martin A. Estermann, Humphrey Hung-Chang Yao

**Affiliations:** ^1^ Reproductive Developmental Biology Group, National Institute of Environmental Health Sciences, Research Triangle Park, Durham, NC 27709, USA.; ^2^ Epigenetic and RNA Biology Laboratory, National Institute of Environmental Health Sciences, Research Triangle Park, Durham, NC 27709, USA.; ^3^ Integrative Bioinformatics Support Group, National Institute of Environmental Health Sciences, Research Triangle Park, Durham, NC 27709, USA.

## Abstract

Sex determination of mammalian gonads hinges upon sex-specific differentiation of gonadal supporting cells: Sertoli cells in the testis and granulosa cells in the ovary. To gain insights into how supporting cells acquire their identities, we performed joint single-nucleus transcriptomics and chromatin accessibility assays on murine gonadal cells during sex determination. By contrasting sex-specific gene expression and corresponding chromatin accessibility among progenitor and differentiated cells, we found that sex-specific chromatin regions in supporting cells are established shortly after sex determination, accompanied by the acquisition of active histone marks. The presence of potential transcription factor–binding motifs in the open chromatin regions revealed regulatory networks underlying ovary-enriched factors LEF1 and MSX1, which promote granulosa fate by inducing granulosa-specific genes such as *Foxl2* and *Fst*. Our results not only identify the gene regulatory framework underlying supporting cell sex differentiation but also provide invaluable resources for the field.

## Teaser

Sex-specific changes in chromatin and gene expression underlie the divergent development of the bipotential gonadal primordium.

## INTRODUCTION

The gonadal primordium, also known as the genital ridge, is among the mammalian tissues that have two divergent developmental fates: testis or ovary. In mouse embryos, the bipotential gonadal primordium arises from the epithelium along the coelomic surface of the mesonephros around embryonic day 10 (E10) (*1*, *2*). As the coelomic epithelium proliferates and constitutes the somatic compartment of the gonadal primordium, it gives rise to both the supporting and interstitial cell lineages (*2*, *3*). The supporting cell precursors, or presupporting cells, show minimal sex differences and remain bipotential before sex determination. In the XY mouse gonad, presupporting cells up-regulate the Y-linked gene *Sry* (*4*, *5*), which in turn induces the expression of its downstream effector *Sox9* (*6*). *Sox9*, along with other factors including *Fgf9* (*7*) and *Pgd2* (*8*), promotes the differentiation of presupporting cells into Sertoli cells (*9*). Conversely, in the XX mouse gonad where *Sry* is absent, presupporting cells give rise to pregranulosa cells that eventually encapsulate female germ cells and form ovarian follicles perinatally (*10*). Following sex determination, the gonadal epithelium continues to differentiate into pregranulosa cells, which occurs only in ovaries but not testes, contributing to the second wave of follicle formation (*11*, *12*). The differentiation of pregranulosa cells appears to be multifactorial. Factors promoting pregranulosa cells differentiation include *Wnt4* (*13*), *Foxl2* (*14*), *Fst* (*15*), *Runx1* (*16*), and the -KTS variant of *Wt1* (*17*). Dysregulation of these factors results in not only complete or partial sex reversal in mice but also differences of sexual development (DSDs) in humans (*13*, *16*–*21*). Despite the identification of these ovary-determining factors, the precise sequence of events driving pregranulosa cell differentiation remains elusive.

Changes in chromatin accessibility are associated with supporting cell differentiation in the murine gonad (*22*, *23*). In somatic progenitor cells, most open chromatin regions are shared between sexes. In addition, genes regulating sex differentiation are bivalently marked with both active trimethylation of histone H3 lysine 4 (H3K4me3) and repressive H3K27me3 histone marks at their promoters (*24*). By E13.5, when Sertoli and pregranulosa fates are established, more than 50% of open chromatin regions become specific to either population. This acquisition of sex-specific accessible chromatin regions is accompanied by the loss of repressive H3K27me3 histone marks of the opposite sex (*24*). These observations suggest that the increase in sex-specific open chromatin regions may promote transcriptional programs unique to Sertoli or pregranulosa cell fate.

To investigate supporting cell differentiation, we applied joint single-nucleus transcriptomics and chromatin accessibility assays (10x Genomics Multiome) in this study. This approach simultaneously profiles nuclear mRNA and DNA from individual nuclei, enabling direct linkage of gene expression and accessible chromatin profiles from the same cells. By profiling time points across early gonadal development, we examined how the acquisition of unique chromatin profiles relates to supporting cell fate commitment. In addition, by mapping enriched transcription factor (TF) motifs within pregranulosa open chromatin regions, we uncovered the transcriptional regulatory networks driving pregranulosa cell differentiation.

## RESULTS

### snRNA-seq of mouse embryonic gonads recapitulates cell types identified through single-cell RNA-seq

**Fig. 1.**
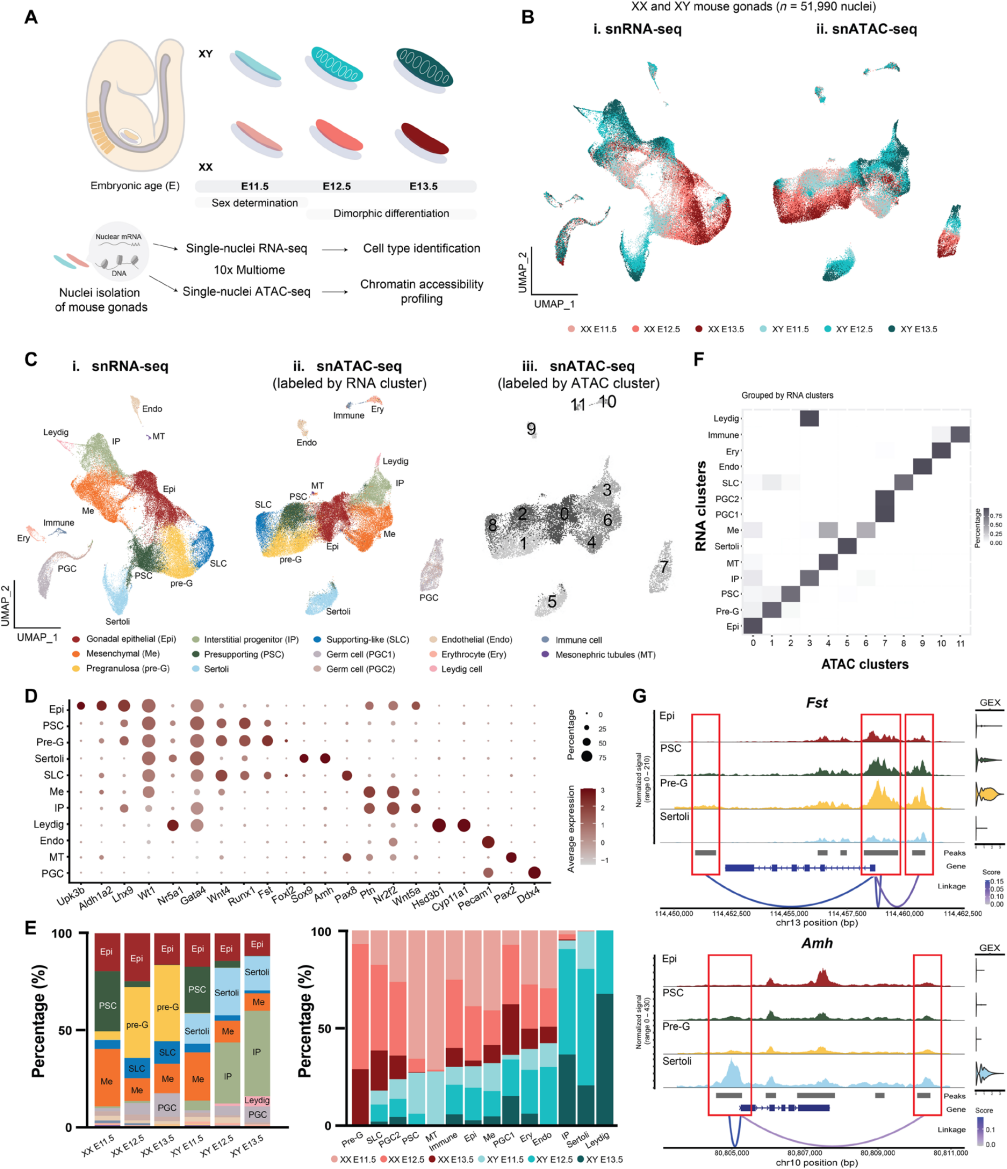
Joint single-nucleus multiomics of mouse gonadal cells during sex determination reveals highly correlated chromatin accessibility and gene expression profiles among different cell types. (**A**) Schematic of joint snRNA-seq and ATAC-seq (10x Genomics Multiome) on mouse XX and XY gonadal cells at E11.5 (initiation of sex determination), E12.5 (onset of morphological differentiation), and E13.5 (morphological dimorphism). After nucleus isolation, nuclear mRNA was used for snRNA-seq and cell type annotation, while DNA from the same nucleus was used for snATAC-seq and chromatin accessibility profiling. (**B**) UMAP of snRNA-seq (i) and snATAC-seq (ii) data collected from E11.5 to E13.5 XX and XY gonadal cells color coded by embryonic sex and stage. (**C**) UMAP of snRNA-seq (i) and snATAC-seq (ii and iii) data collected from E11.5 to E13.5 XX and XY gonadal cells color coded by unbiased clustering based on either RNA (i and ii) or ATAC data (iii). Annotations for the snRNA-seq clusters are: gonadal epithelial (Epi), mesenchymal (Me), pregranulosa (pre-G), interstitial progenitor (IP), presupporting (PSC), supporting-like cell (SLC), primordial germ cell (PGC), endothelial cell (Endo), erythrocyte (Ery), and mesonephric tubule (MT). (**D**) Marker gene expression for cell type annotation based on RNA clustering. (**E**) Percentage of cell type composition within each embryonic sex and stage (left), and percentage of sex and stage composition within each cell type (right). (**F**) Heatmap of cluster distribution of cells based on ATAC clustering (*x* axis) and RNA clustering (*y* axis), as grouped by individual RNA cluster (the sum of each row is 1). (**G**) Peak-gene linkage plots of *Fst* (top) and *Amh* (bottom) in epithelial (EPI), presupporting (PSC), pregranulosa (pre-G), and Sertoli cells of combined sex and developmental stages. The red boxes denote called peaks (gray bars) that are linked to gene expression (GEX). Linkage score represents the level of association between chromatin accessibility and gene expression.

To delineate sex-specific transcriptional networks underlying somatic cell differentiation in the gonad, we performed joint single-nucleus RNA sequencing (snRNA-seq) and Assay for Transposase-Accessible Chromatin (ATAC) sequencing (ATAC-seq) (10x Genomics Multiome) on mouse XX and XY gonadal cells at E11.5 (initiation of sex determination), E12.5 (onset of morphological differentiation), and E13.5 (morphological dimorphism) (Fig. 1A). The assay allowed us to obtain information on gene expression and chromatin status from the same nuclei simultaneously. After quality control and filtering (*25*), we profiled a total of 51,990 nuclei (12,335, 13,625, and 5704 for the XX, and 5134, 10,246, and 4946 for the XY nuclei at E11.5, E12.5, and E13.5, respectively) (fig. S1). We first projected the snRNA-seq data of individual nucleus onto lower dimensional space using the Uniform Manifold Approximation and Projection (UMAP). We observed that the transcriptomes of XX and XY nuclei overlapped extensively at E11.5 and diverged apart by sex at E12.5 and E13.5 (Fig. 1B, i, and figs. S2 and S3A). Upon clustering based on RNA data and analyzing the expression of genes known for various cell types (*26*), we assigned 14 major cell populations, including germ cells, epithelial cells, mesenchymal cells, pregranulosa cells, Sertoli cells, presupporting cells, and supporting-like cells, which constitute the future rete testis and rete ovarii (Fig. 1C, i, and fig. S2A) (*26*). Specifically, gonadal epithelial cells were enriched with *Upk3b* and *Aldh1a2* expression (*27*, *28*); presupporting cells expressed *Wnt4* and *Runx1* and were present in both sexes at E11.5 (*16*); supporting-like cells were enriched with *Pax8* expression (*26*); while pregranulosa and Sertoli cells were sex-specific clusters and expressed *Fst* and *Sox9,* respectively (Fig. 1D) (*15*, *29*). Consistent with other single cell RNA-seq datasets (*26*, *30*), we observed a drastic decrease of the percentage of presupporting cells from E11.5 to E12.5 in both XX and XY gonads, accompanied by the emergence of pregranulosa cells in E12.5 XX gonads and interstitial progenitor (IP) cells in E12.5 XY gonads (Fig. 1E and fig. S4, A to D). While we took advantage of the *Nr5a1-Cre*; *Rosa-tdTomato* embryos to assist with the separation of the gonad from the mesonephros, we observed a small cluster of cells from the mesonephros, i.e., *Pax2-* and *Pax8*-enriched mesonephric tubules (Fig. 1, C and D, 0.6%) (*26*). Overall, our snRNA-seq dataset, which aligned with published single-cell RNA-seq datasets, produced reliable transcriptomic basis for further downstream chromatin analysis.

### Chromatin accessibility and gene expression profiles of gonadal somatic cells are highly correlated

To capture cell type–specific ATAC peaks, we performed peak calling using MACS2 (*31*) on individual RNA clusters. Similar to snRNA-seq data, UMAP of snATAC-seq data showed overlapping of XX and XY nuclei at E11.5 and divergence by sex at E12.5 and E13.5 (Fig. 1B, ii, and figs. S2 and S3B). Upon clustering based on ATAC data, we observed that chromatin states generally correlated with gene expression among major cell types (Fig. 1C, ii and iii, and F). Despite the correlation, small proportions of presupporting cells and supporting-like cells shared the same ATAC cluster with pregranulosa cells, indicating a closer similarity in their chromatin landscape (Fig. 1F and fig. S5). In addition, mesenchymal cells encompassed various chromatin states, while fetal Leydig cells shared similar chromatin profile with IPs at the applied resolution (Fig. 1F). Last, to correlate accessible chromatin peaks to gene expression, we performed linkage analysis (*32*) on the whole dataset to obtain peaks and genes that were statistically significantly associated with one another within transcription start site (TSS) ± 500 kb (linkage distance) as compared across cell types (data S1). This analysis computes the correlation between gene expression and accessibility of each peak within the linkage distance. Hence, a single gene may be linked to multiple peaks, and a single peak may be associated with multiple genes. With the approach, we detected the testis-specific enhancer of *Sox9* (*33*, *34*), characterized by a prominent accessible peak located 13 kb upstream of *Sox9* TSS in Sertoli cells, which showed a positive correlation with *Sox9* expression (fig. S6A).

Using canonical pregranulosa cell marker *Fst* and Sertoli marker *Amh* as examples, we identified that the promoter peak and the peaks right upstream and downstream of *Fst* were positively correlated to *Fst* expression (Fig. 1G). These peaks were accessible in epithelial, presupporting, and pregranulosa cells, but not in Sertoli cells, whereas the promoter peak and a peak downstream of *Amh* were positively correlated to *Amh* expression (Fig. 1G). These two peaks were only accessible in Sertoli cells but not in pregranulosa and precursor cell types. In contrast, the intragenic peaks of *Amh* were not associated to *Amh* expression as they were also present in other cell types (Fig. 1G). Together, our analyses revealed the dynamic changes in chromatin modulation during gonadal cell differentiation and provided an opportunity to investigate how changes in chromatin accessibility across cell types and developmental time points are associated with changes in gene expression.

### Sex differences in chromatin accessibility between Sertoli and pregranulosa cells increase during E11.5 to E12.5 transition

**Fig. 2.**
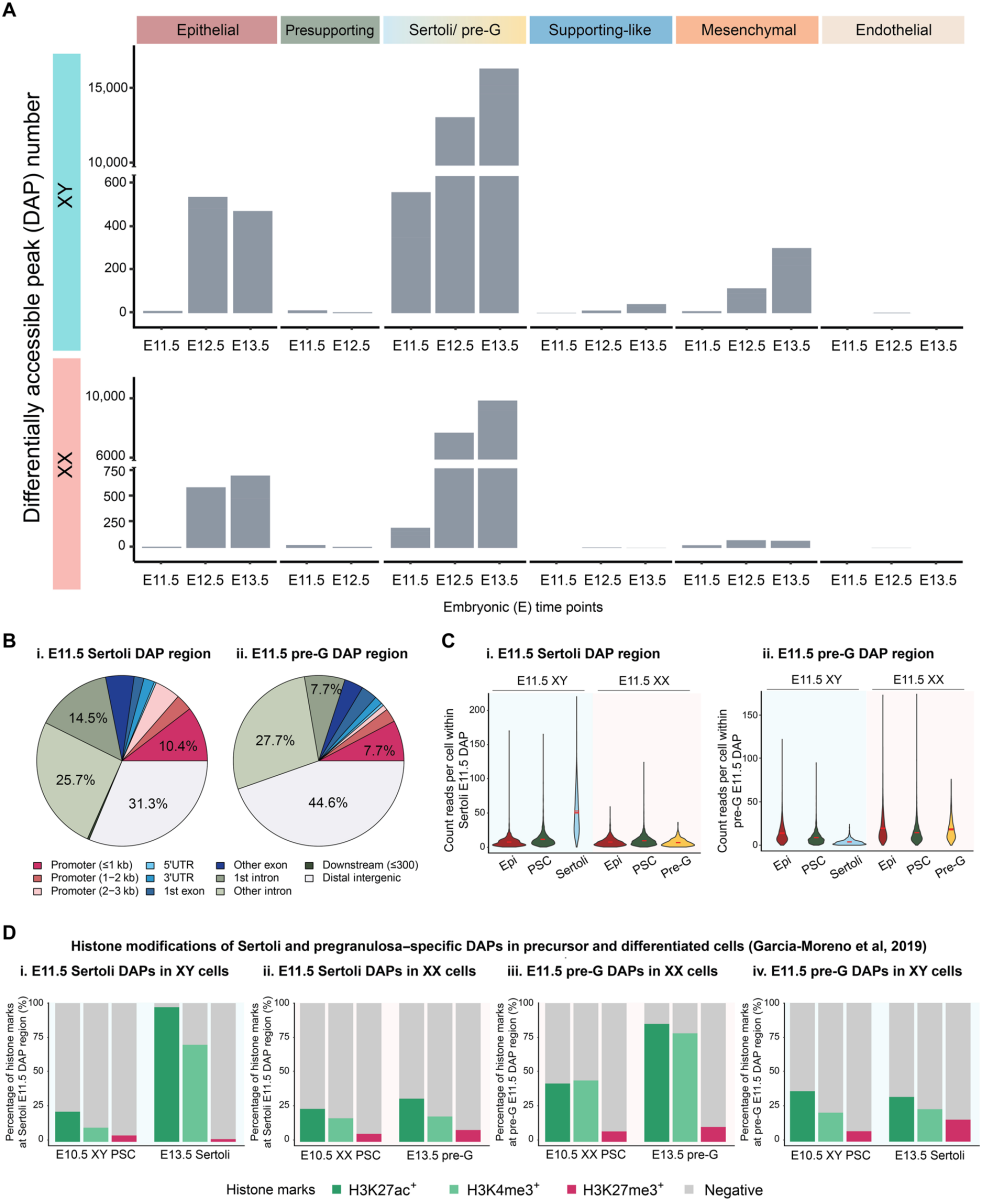
Sertoli and pregranulosa cells undergo active chromatin and epigenetic changes during sex differentiation. (**A**) Numbers of differentially accessible peaks (DAPs) between sexes across cell types and developmental time points. (**B**) Peak annotation within E11.5 Sertoli DAPs (i) and E11.5 pregranulosa cell DAPs (ii). (**C**) Chromatin accessibility, measured by average count reads per cell within E11.5 Sertoli DAPs (i) and pregranulosa E11.5 DAPs (ii) of E11.5 XX and XY epithelial, presupporting cells, Sertoli, and pregranulosa cells. (**D**) Percentage of H3K27ac, H3K4me3, and H3K27me3-positive chromatin regions [obtained from published ChIP-seq datasets, Garcia-Moreno *et al.*, 2019 (*22*, *24*)] that overlap with E11.5 Sertoli DAPs (i and ii) and E11.5 pregranulosa DAPs (iii and iv) in XY (i and iv) and XX (ii and iii) presupporting, Sertoli, or pregranulosa cells.

To understand how chromatin profiles of gonadal somatic cells change during sex determination, we compared accessible chromatin regions (differential accessible peaks, DAPs) between sexes across cell types and developmental time points in the whole genome (Fig. 2A, with XY data on the top and XX data on the bottom, data S2). The greatest number of DAPs and the most changes in DAP numbers were observed between Sertoli and pregranulosa cells from E11.5 to E12.5, followed by epithelial cells (Fig. 2A). The XY mesenchymal cells also showed drastic changes in sex-specific chromatin pattern from E11.5 to E12.5. On the other hand, presupporting cells, supporting-like cells, and endothelial cells showed minimal sex differences in terms of accessible chromatin regions (Fig. 2A). Sertoli cells displayed nearly three times (E11.5) and twice (E12.5) as many DAPs as pregranulosa cells, reflecting the delayed timeline in pregranulosa differentiation (*11*). Upon annotating the genomic region of all DAPs, we found that most Sertoli and pregranulosa-specific DAPs at E11.5 were located within distal intergenic regions and other intronic regions, followed by the promoter regions (Fig. 2B).

To explore how Sertoli and pregranulosa cells acquired sex-specific DAPs by E11.5, we measured chromatin accessibility within E11.5 Sertoli DAPs (537 unique peaks) and pregranulosa E11.5 DAPs (195 unique peaks) in the precursor cell types, namely E11.5 XX and XY epithelial cells and presupporting cells (cell type presumably derived from the epithelial cells) (Fig. 2C). We found that the average accessibility within E11.5 Sertoli DAPs was significantly higher in Sertoli than in pregranulosa cells as expected. In XY gonads, the average accessibility of Sertoli DPAs increased slightly in presupporting cells and then further increased drastically in Sertoli cells as compared to XY presupporting and epithelial cells. Such increase was not observed in pregranulosa cells (Fig. 2C, i). On the other hand, the average accessibility within E11.5 pregranulosa DAPs was significantly higher in pregranulosa than in Sertoli cells, albeit at a lower degree than the difference within E11.5 Sertoli DAPs (Fig. 2C, ii). In XX gonads, the average accessibility of pregranulosa DAPs decreased slightly in presupporting cells and then increased slightly in pregranulosa cells as compared to XX presupporting cells. In XY gonads, average accessibility of pregranulosa DAPs decreased slightly in presupporting and then decreased drastically in Sertoli cells as compared to XY epithelial cells (Fig. 2C, ii). When examining Sertoli and pregranulosa E12.5 DAPs (12,991 and 7727 unique peaks respectively), we observed a similar trend within Sertoli DAPs, while partial pregranulosa cells exhibited highest accessibility level within pregranulosa DAPs (fig. S7, A and B). We further examined changes in the accessibility of E13.5 pregranulosa-specific chromatin regions in pregranulosa cells across developmental stages. We observed a continuous increase in accessibility throughout time points in pregranulosa cells. This suggested that the acquisition of pregranulosa DAPs occurred not only passively through Sertoli cells decreasing accessibility in these regions but also actively with pregranulosa cells gaining chromatin accessibility (fig. S7C). These results indicated that Sertoli and pregranulosa cells undergo active chromatin remodeling during sex differentiation, with Sertoli cells exhibiting a greater change. During Sertoli cell differentiation, Sertoli DAPs become more accessible, while regions with pregranulosa DAPs become less accessible, whereas during pregranulosa differentiation, chromatin regions with Sertoli and pregranulosa DAPs alter in much less extent as compared to Sertoli cells.

### Sertoli and pregranulosa specific chromatin regions acquire distinctive active histone marks during sex differentiation

Sertoli and pregranulosa-enriched genes are bivalent at their promoter regions before sex determination, marked by both active H3K4me3 and repressive H3K27me3 histone marks (*24*). During sex determination, genes promoting one fate lose the repressive H3K27me3 marks, while genes promoting the alternate fate remain bivalent (*24*). To explore how the acquisition of Sertoli and pregranulosa-specific DAPs correlates with changes in histone marks, we examined the status of histone modifications within these DAPs in precursor and differentiated cells. We analyzed published chromatin immunoprecipitation sequencing (ChIP-seq) data for H3K4me3, H3K27me3, and the active acetylation of histone H3 lysine 27 (H3K27ac) from E10.5 purified mouse bipotential progenitor cells (referred to as presupporting cells or PSCs hereafter) and E13.5 Sertoli and pregranulosa cells (*22*, *24*) and investigated their associations with the DAPs in Sertoli cells and pregranulosa cells (Fig. 2D). We observed a drastic increase in active H3K4me3 and H3K27ac histone marks within Sertoli-specific DAPs (537 unique peaks) during Sertoli cell differentiation. The proportion of H3K4me3-positive Sertoli-specific DAPs rose from 10% in E10.5 presupporting to 70% in E13.5 Sertoli cells, while H3K27ac-positive Sertoli-specific DAPs increased from 22% in E10.5 presupporting to more than 97% in E13.5 Sertoli cells (Fig. 2D, i). In contrast, the levels of H3K4me3 and H3K27ac-positive Sertoli-specific DAPs showed minimal changes during pregranulosa differentiation (Fig. 2D, ii). For pregranulosa-specific DAPs (195 unique peaks), E10.5 XX presupporting cells displayed higher levels of H3K4me3 and H3K27ac marks (45 and 42%, respectively) (Fig. 2D, iii) as compared to Sertoli-specific DAPs in E10.5 XY presupporting cells (Fig. 2D, i). Following pregranulosa cell differentiation, the proportion of H3K4me3 and H3K27ac-positive pregranulosa-specific DAPs increased to 78 and 85%, respectively (Fig. 2D, iii). H3K4me3 and H3K27ac-positive pregranulosa-specific DAPs remained at a similar level during Sertoli cell differentiation (Fig. 2D, iv).

For the repressive H3K27me3 modifications, we did not observe significant changes overall. Within Sertoli-specific DAPs, H3K27me3 level decreased from 5% in E10.5 XY presupporting to 2% in E13.5 Sertoli cells (Fig. 2D, i), whereas H3K27me3 level increased slightly from 6% in E10.5 XX presupporting to 9% in E13.5 pregranulosa (Fig. 2D, ii). Within pregranulosa-specific DAPs, H3K27me3 level increased both in Sertoli (8 to 16%) (Fig. 2D, iv) and pregranulosa cells (8 to 11%) (Fig. 2D, iii). Upon overlapping the active H3K4me3 and the repressive H3K27me3 peaks, we found that only 8 of 537 E11.5 Sertoli cell–specific DAPs were bivalent for both H3K4me3 and H3K27me3 marks in E10.5 XY presupporting cells. Among them, three were linked to genes *Myh14*, *Rpl13a*, and *Gramd2*. In the case of pregranulosa cells, 13 of 195 E11.5 DAPs were bivalent for both histone marks in E10.5 XX presupporting cells. Among them, four were linked to genes *Ereg*, *Cbln1*, *Sp5*, and *Rpl12* (table S1). Similar histone methylation and acetylation patterns were observed when we compared Sertoli and pregranulosa E13.5 DAPs (16,235 and 9886 unique peaks, respectively) in presupporting and differentiated cells (fig. S8). Collectively, these results suggested that the acquisition of sex-specific accessible chromatin regions is accompanied by the deposition of active H3K4me3 and H3K27ac histone marks. Sertoli cells exhibited a four- to sevenfold increase, while pregranulosa cells showed a twofold increase, reflecting broader changes in chromatin accessibility in Sertoli cells compared to pregranulosa cells (Fig. 1C, ii).

### Increase in Sertoli and pregranulosa-specific gene expression from E11.5 to E12.5 is associated with changes in chromatin accessibility

**Fig. 3.**
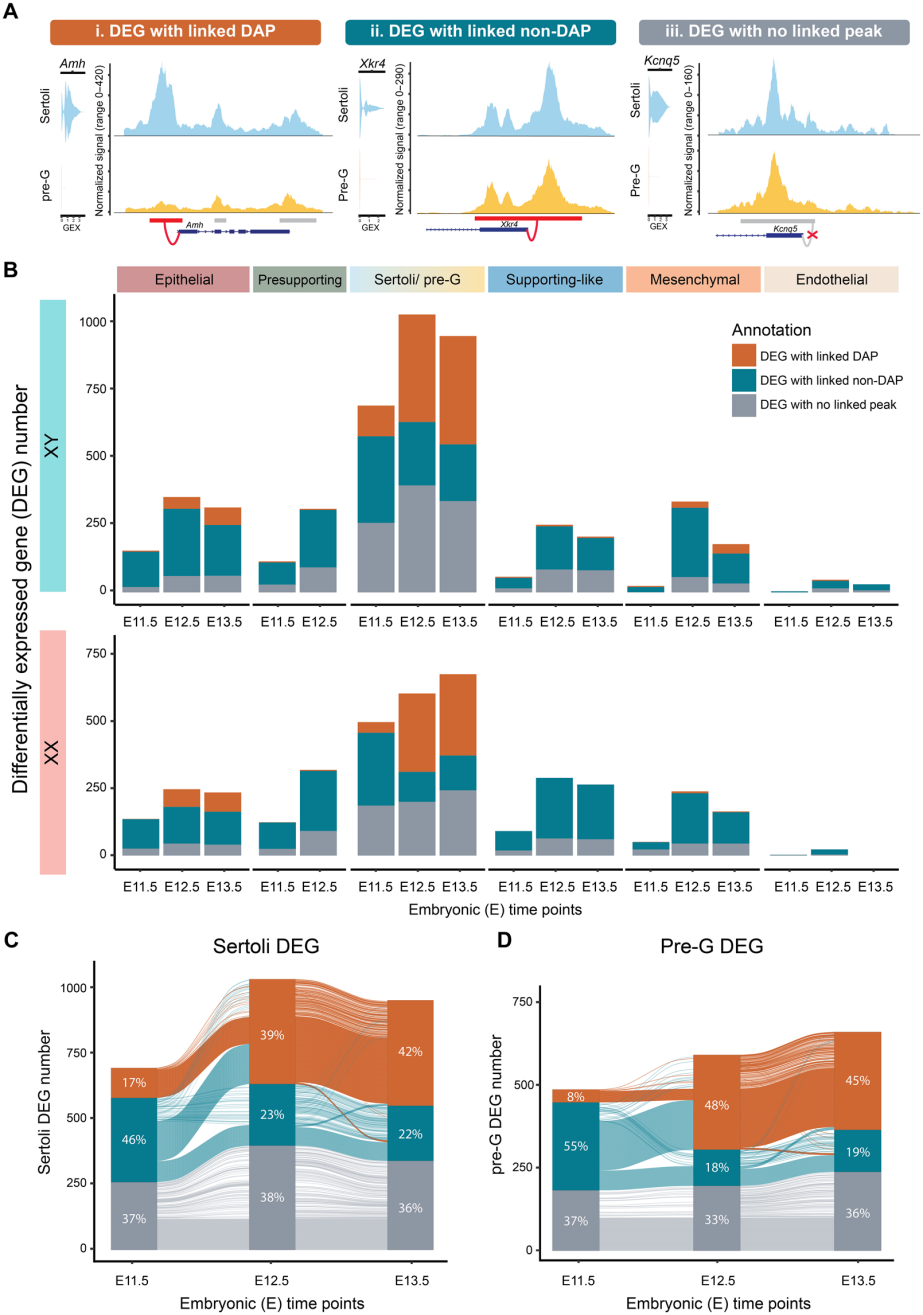
The association between Sertoli and pregranulosa-specific gene expression and differential chromatin pattern increases during sex determination. (**A**) Categorization and examples of DEGs with linked DAPs between sexes (i), linked to chromatin-accessible peaks that were not DAPs between sexes (ii), or not linked to any peaks (iii). (**B**) Numbers of DEGs between sexes across cell types and developmental time points, annotated with linkage categorization. (**C** and **D**) Changes in the annotation of three DEG categories in Sertoli (C) and pregranulosa cells (D) over developmental time points visualized with Sankey diagram.

Given the dynamic changes in chromatin landscape observed during supporting cell differentiation, we sought to determine how these alterations in chromatin accessibility are associated with gene expression. We first obtained differentially expressed genes (DEGs) between sexes across cell types and developmental time points. We then categorized DEGs that were (i) positively or negatively linked to any DAPs, (ii) positively or negatively linked to chromatin-accessible peaks that were not DAPs between sexes, or (iii) not linked to any peaks at all (Fig. 3A and data S3). For the first category, we defined DEGs with at least one linked DAP as “DEG with linked DAP.” An example of this category was *Amh*, a Sertoli DEG. Expression of *Amh* was linked to the *Amh* promoter peak, which was differentially accessible between Sertoli and pregranulosa cells (Fig. 3A, i). In the second category, when a DEG was linked to a chromatin-accessible peak, however, the peak itself was not a DAP, we classified it “DEG with linked non-DAP” (Fig. 3A, ii). For instance, the scramblase-encoding gene *Xkr4* was identified as a Sertoli DEG compared to pregranulosa cells. The expression of *Xkr4* was linked to its promoter peak, as analyzing across the entire dataset, this promoter peak was accessible and the gene was expressed in Sertoli cells, whereas in another cell type, such as immune cells, the promoter region of *Xkr4* was not accessible and the gene was not expressed (Fig. 3A, ii, and fig. S6B). Although *Xkr4* was a Sertoli DEG, its promoter peak was not a DAP between Sertoli and pregranulosa cells, rendering it a DEG with linked non-DAP between the two cell types (Fig. 3A, ii). In the third category, an example was the Potassium voltage–gated channel *Kcnq5*, a Sertoli DEG*.* Its expression was not associated with any present peaks within the TSS ± 500 kb linkage distance across cell types (fig. S6C); therefore it was assigned as “DEG with no linked peak” (Fig. 3A, iii).

On the basis of this analysis, we observed that Sertoli and pregranulosa cells exhibited the highest number of DEGs across all time points from E11.5 to E13.5 (Fig. 3B, with XY data on the top and XX data on the bottom). The DEGs with no linked peak accounted for more than 30% of DEGs, suggesting the possibility that expression of these DEGs is regulated by sex-specific TF binding in already accessible chromatin regions or through potential distal enhancer action beyond the linkage distance. Strikingly, the proportion of DEG with linked DAP increased from 16 to 38% in Sertoli and 8 to 48% in pregranulosa cells between E11.5 and E12.5 (Fig. 3B). In epithelial, presupporting, supporting-like, and mesenchymal cells, an increase in DEG number was observed between E11.5 and E12.5, with most DEGs linked to non-DAPs (Fig. 3B).

To explore the dynamics of peak-gene association across time points, we visualized changes in the annotation of three DEG categories in Sertoli and pregranulosa cells (Fig. 3, C and D). We observed that DEG with linked DAP at E11.5 retained the same annotation across time points. In contrast, a large proportion of DEGs with linked non-DAPs at E11.5 converted to DEG with linked DAP at E12.5, accounting for 53% in Sertoli cells and 60% in pregranulosa cells (teal to orange transition in Fig. 3, C and D). Although the number of DEGs with no linked peaks increased slightly, it was not due to conversion of other two categories. The distribution of the three DEG categories stabilized from E12.5 to E13.5 (Fig. 3, C and D). These results indicated that Sertoli and pregranulosa cells undergo active chromatin remodeling, particularly during the transitioning from E11.5 to E12.5. The observed increase in sex-specific gene expression that were linked to changes in chromatin status suggested potential modulation through TF binding during early differentiation.

### Motif analysis reveals TF network underlying Sertoli differentiation

**Fig. 4.**
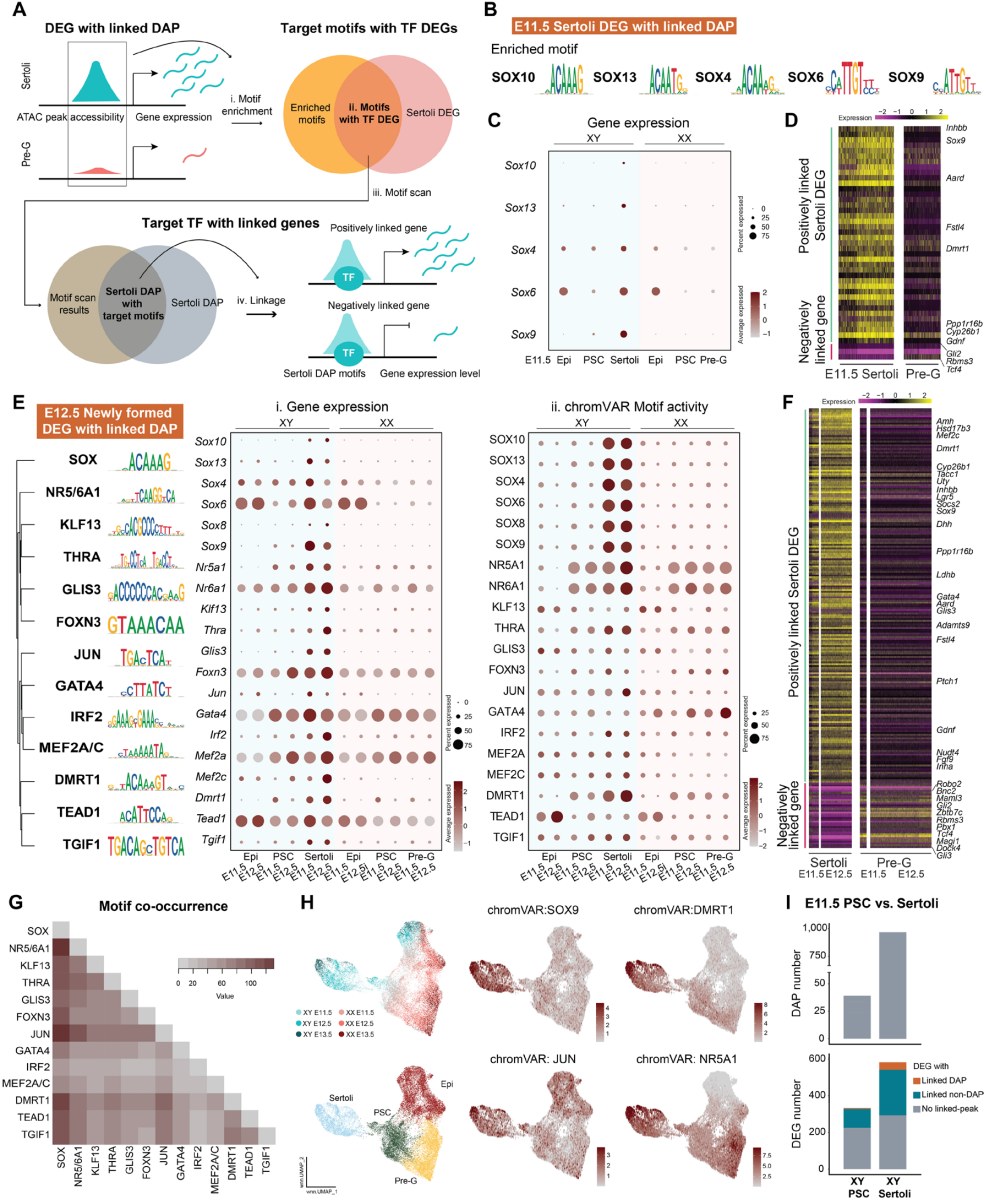
The transcription regulatory network underlying Sertoli cell differentiation. (**A**) Workflow of TF-binding motif analysis: Motif enrichment was first performed on Sertoli DAPs derived from DEG with linked DAP (i). Candidate motifs with expressed TFs were then identified by overlapping the motifs with Sertoli DEGs (ii). After this, motif scan was performed to determine motif position within Sertoli DAPs (iii). Last, downstream target genes of the motifs were identified on the basis of the linkage analysis (iv). (**B**) Identified motifs enriched within “E11.5 Sertoli DEGs with linked DAP,” with position weight matrix of the corresponding motifs. (**C**) Gene expression of the identified TFs in E11.5 XX and XY epithelial (Epi), presupporting (PSC), Sertoli, and pregranulosa cells. (**D**) Gene expression heatmap of the positively (green) and negatively (magenta) linked downstream target genes of the identified TFs in E11.5 Sertoli and pregranulosa cells. (**E**) Identified motifs enriched within E12.5 Sertoli DEGs with linked DAP, with position weight matrix of the corresponding motifs. Motifs were clustered on the basis of downstream target gene enrichment. Gene expression (i) and chromVAR motif activity (ii) of the identified TFs in E11.5 and E12.5 XX and XY epithelial, presupporting, Sertoli, and pregranulosa cells were shown. (**F**) Gene expression heatmap of the positively (green) and negatively (magenta) linked downstream target genes of the identified TFs in E11.5 and E12.5 Sertoli and pregranulosa cells. (**G**) Heatmap of motif co-occurrence based on the incidence (value) of motif sites enriched among E12.5 Sertoli DEGs with linked DAP associating with the same downstream target genes. (**H**) Feature plots of chromVAR motif activity of identified motifs on joint RNA and ATAC UMAP composed of epithelial, presupporting, Sertoli, and pregranulosa cells from both sexes throughout developmental time points. (**I**) Numbers of DAPs (top) and annotated DEGs (bottom) between E11.5 presupporting cells and Sertoli cells.

Given that the dynamic changes in sex-specific chromatin-accessible regions (DAPs) are associated with DEGs during supporting cell differentiation from E11.5 to E12.5, we hypothesized that TF-binding motifs enriched within E11.5 DAPs could be responsible for early dimorphic differentiation, while those enriched within E12.5 DAPs represent the downstream, secondary factors. These TFs together constitute the transcriptional regulatory network underlying supporting cell differentiation. To explore such network driving Sertoli cell differentiation, we performed TF-binding motif analysis using the workflow outlined in Fig. 4A. First, we performed TF motif enrichment analysis on Sertoli DAPs derived from DEG with linked DAP. Second, we overlapped the enriched motifs and Sertoli cell DEGs and identified candidate motifs with enriched TFs as DEGs. Last, we performed motif scan analysis (*35*) to determine TF motif positions within Sertoli DAPs and established the association of putative TFs with their downstream target genes (Fig. 4A). TF-binding motif analysis on E11.5 Sertoli DEG with linked DAP (Fig. 3C, E11.5 orange, in total 131 unique peaks) revealed five significantly enriched SOX TFs, SOX10, SOX13, SOX4, SOX6, and SOX9 as early TFs regulating Sertoli differentiation (Fig. 4B and data S4). Gene expression of these SOX factors showed partial expression in E11.5 XX and XY epithelial cells, low level of expression in XY presupporting cells, and an up-regulation in Sertoli cells. The expression was absent in XX presupporting and pregranulosa cells (Fig. 4C). Upon locating SOX-binding motifs within Sertoli DAPs and associating these DAPs with expression of other genes, we found that SOX motif–enriched DAPs were positively linked to the expression of Sertoli-enriched genes, including *Sox9*, *Dmrt1*, *Inhbb*, *Fstl4*, and *Cyp26b1*. Conversely, SOX motif–enriched DAPs were negatively linked to genes absent in Sertoli cells, such as *Gli2*, *Tcf4*, and *Rbms3* (Fig. 4D). Among the three negatively associated genes, *Gli2* and *Tcf4* showed higher expression in pregranulosa cells (Fig. 4D).

We next identified the downstream, secondary TFs by searching the enriched TF motifs within Sertoli-specific chromatin regions that were associated with Sertoli DEGs at E12.5 (Fig. 3C, E12.5 orange). Specifically, we analyzed two groups of accessible regions: those that converted from DEGs with linked non-DAP to DEGs with linked DAP (teal to orange transition from E11.5 to E12.5 in Fig. 3C), as well as DEG with linked DAP that appeared de novo at E12.5 (excluding orange to orange transition from E11.5 to E12.5 in Fig. 3C). We named these two groups with a total of 667 unique peaks “E12.5 newly formed DEG with linked DAP.” TF-binding motif analysis on DAPs of these two groups revealed many more motifs with enriched and expressed TFs such as DMRT1, NR5A1, GATA4, NR6A1, JUN, and interferon regulatory factor 2 (IRF2) in addition to SOX factors (Fig. 4E and data S4). Different gene expression patterns of these TFs were observed: Factors including *Sox4*, *Sox6*, *Nr6a1*, and *Tead1* showed expression starting in XY epithelial cells and increased expression in presupporting cells and Sertoli cells. On the other hand, *Sox9*, *Nr5a1*, *Foxn3*, *Gata4*, and *Mef2a* showed expression starting in XY presupporting cells and continued in Sertoli cells. Last, *Sox10*, *Sox13*, *Sox8*, *Klf13*, *Thra*, *Glis3*, *Irf2*, and *Tgif1* showed increased expression only in Sertoli cells (Fig. 4E, i). Gene expression of most of these TFs was absent in XX cells with few exceptions: *Sox6* and *Tead1* were observed in XX epithelial cells but not in presupporting or pregranulosa cells; *Gata4* and *Mef2a* were observed in presupporting and pregranulosa cells despite in lower level than Sertoli cells (Fig. 4E, i). The enriched TFs were positively associated with the expression of Sertoli DEGs such as *Amh*, *Lgr5*, *Dhh*, *Ptch1*, and *Inha*, while negatively associated with the expression of *Bnc2*, *Zbtb7c*, *Pbx1*, *Tcf4*, and *Gli3* (Fig. 4F). Many of the negatively associated genes were expressed in higher level in pregranulosa cells (Fig. 4F). To investigate whether these TFs can regulate the same target genes, we generated a motif co-occurrence matrix by calculating the incidence of TFs within motif sites that were associated with the same target genes. The highest co-occurrence of TF-binding sites was found in SOX with NR5/6A1 and JUN, followed by SOX with DMRT1 and NR5/6A1 with JUN (Fig. 4G).

To provide further evidence for the identification of these putative TFs, we applied chromVAR (*36*), a program that infers TF-associated chromatin accessibility, or motif activity, directly on single-cell ATAC-seq data. We found that SOX TFs, DMRT1, thyroid hormone receptor alpha (THRA), and JUN showed high motif activity in Sertoli cells (Fig. 4E, ii). ChromVAR also predicted that NR5A1 and NR6A1 were active motifs in both XX and XY presupporting cells but with enhanced activity in E12.5 Sertoli cells (Fig. 4E, ii). To visualize the motif activity at the single-cell level, we plotted chromVAR motif activity score on a joint RNA and ATAC UMAP of epithelial, presupporting, Sertoli, and pregranulosa cells (Fig. 4H). The SOX9 motif activity increased in Sertoli cells, while DMRT1 activity exhibited a gradual increase in presupporting cells and peaked in Sertoli cells. The motif activity for JUN showed broad distribution, with its highest level observed in Sertoli cells. Last, NR5A1 motif activity increased in presupporting cells and peaked in both Sertoli and pregranulosa cells (Fig. 4H). The observation that some motifs showed increased activity in presupporting cells before Sertoli cells, while others peaked directly in Sertoli cells prompted us to compare the two cell types directly. Comparing E11.5 XY presupporting cells with E11.5 Sertoli cells, we found that Sertoli cells exhibited a 24-fold increase in DAPs and a 1.8-fold increase in DEGs, suggesting that Sertoli cells underwent active chromatin remodeling and acquired Sertoli-specific chromatin pattern during cell differentiation readily by E11.5 (Fig. 4I). Among DEGs linked to DAPs, genes including *Msx1* and *Emx2* were down-regulated in presupporting cells, whereas *Sox9*, *Sox10*, *Lgr5*, and *Amh* displayed increased expression in Sertoli cells (data S5 and S6). These genes likely were regulated from changes on the chromatin level during presupporting to Sertoli transition.

### Motif analysis reveals LEF1 and MSX1 as initial TFs underlying pregranulosa differentiation

**Fig. 5.**
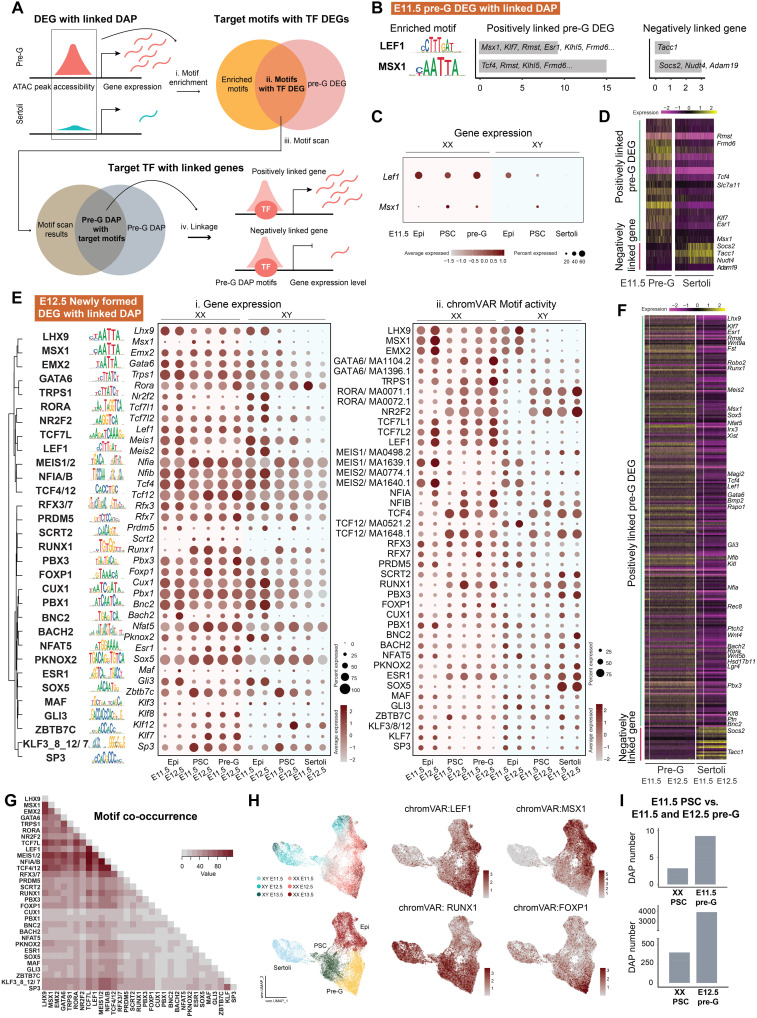
The transcription regulatory network underlying pregranulosa differentiation. (**A**) Workflow of motif analysis: Motif enrichment was first performed on pregranulosa DAPs derived from DEG with linked DAP (i). Candidate motifs with expressed TFs were then identified by overlapping the motifs with pregranulosa DEGs (ii). Then, motif scan was performed to determine motif position within pregranulosa DAPs (iii). Last, downstream target genes of the motifs were identified based on the linkage analysis (iv). (**B**) Identified motifs enriched within E11.5 pregranulosa DEGs with linked DAP, with position weight matrix of the corresponding motifs. (**C**) Gene expression of the identified TFs in E11.5 XX and XY epithelial (Epi), presupporting (PSC), pregranulosa, and Sertoli cells. (**D**) Gene expression heatmap of the positively (green) and negatively (magenta) linked downstream target genes of the identified TFs in E11.5 pregranulosa and Sertoli cells. (**E**) Identified motifs enriched within E12.5 pregranulosa DEGs with linked DAP, with position weight matrix of the corresponding motifs. Motifs were clustered on the basis of downstream target gene enrichment. Gene expression (i) and chromVAR motif activity (ii) of the identified TFs in E11.5 and E12.5 XX and XY epithelial, presupporting, pregranulosa, and Sertoli cells were shown. (**F**) Gene expression heatmap of the positively (green) and negatively (magenta) linked downstream target genes of the identified TFs in E11.5 and E12.5 pregranulosa and Sertoli cells. (**G**) Heatmap of motif co-occurrence based on the incidence (value) of motif sites enriched among E12.5 pregranulosa DEGs with linked DAP associating with the same downstream target genes. (**H**) Feature plots of chromVAR motif activity of identified motifs on joint RNA and ATAC UMAP composed of epithelial, presupporting, Sertoli, and pregranulosa cells from both sexes throughout developmental time points. (**I**) Number of DAPs between E11.5 presupporting cells and E11.5 pregranulosa cells (top) and between E11.5 presupporting cells and E12.5 pregranulosa cells (bottom).

Having established a pipeline that identified known transcriptional regulators of Sertoli cell differentiation as a proof of concept, we applied the same approach to pregranulosa cells. Using this approach, we overlapped the enriched TF motifs within pregranulosa DAPs and pregranulosa DEGs to identify putative TFs. Next, we determined the positions of TF motifs within pregranulosa DAPs and associated them with positively or negatively regulated downstream target genes (Fig. 5A). Such analysis on E11.5 pregranulosa DEGs with linked DAPs (in total 37 unique peaks) revealed LEF1 and MSX1 as the only two TFs with enriched motifs (Fig. 5B and data S4). *Lef1* expression was observed in E11.5 XX epithelial, presupporting, and pregranulosa cells but decreased in E11.5 XY epithelial and presupporting cells, and absent in Sertoli cells. *Msx1* was expressed in both presupporting cells of both sexes and remained expressed in pregranulosa but not in Sertoli cells (Fig. 5C). Linkage analysis of gene expression and chromatin accessibility further identified pregranulosa DEGs, including *Msx1* and *Klf7*, as potential direct targets of LEF1, while *Tcf4* expression could be regulated by MSX1 (Fig. 5, B and D). Conversely, *Tacc1*, *Socs2*, and *Nudt4* were identified as genes negatively linked with LEF and MSX1, with their expression absent in pregranulosa but present in Sertoli cells (Fig. 5D).

We next investigated enriched motifs within the newly formed DEGs with linked DAP regions in E12.5 pregranulosa cells to identify downstream, secondary TFs regulating pregranulosa differentiation. These regions include DEGs that converted from with linked non-DAP at E11.5 to become linked to at least one DAP at E12.5 (teal to orange in Fig. 3B), as well as DEGs with linked DAPs that appeared de novo at E12.5 (orange in Fig. 3B) in total 530 unique peaks. Motif analysis of these regions revealed many more enriched and expressed TFs beyond LEF1 and MSX1 (Fig. 5E and data S4). Among these motifs, BNC2, ZBTB7C, PBX1, TCF4, and GLI3 were negatively linked targets of Sertoli motifs (Fig. 4F). Unlike most Sertoli TFs that increased gene expression in XY cells and were absent in XX cells, most pregranulosa TFs were expressed in XY precursor cells and down-regulated in Sertoli cells (Fig. 5E). We observed expression patterns that remained consistent across XX epithelial, presupporting, and pregranulosa cells, including *Emx2*, *Trps1*, *Lef1*, and *Pbx1*. Factors including *Msx1*, *Scrt2*, *Runx1*, *Klf7*, and *Sp3* were up-regulated in presupporting cells. Notably, no factors showed increased expression exclusively in pregranulosa cells (Fig. 5E). Positively linked pregranulosa DEGs associated with these TFs included several of the identified TFs themselves, along with *Fst*, *Irx3*, *Kitl*, *Wnt4*, and *Lgr4*. Conversely, negatively linked genes, such as *Socs2* and *Tacc1*, were expressed in Sertoli but absent in pregranulosa cells (Fig. 5F). To determine whether these TFs regulate the same target genes, we generated a motif co-occurrence matrix by calculating the incidence of TFs within motif sites that were associated with the same target genes. The analysis revealed a high co-occurrence rate for TCF7L with LEF1, MEIS1/2, NFIA/B, and TCF4/12, followed by LHX9 with MSX1, EMX2, TCF7L, MEIS1/2, and TCF4/12 (Fig. 5G), suggesting that these factors may regulate target gene expression synergistically.

When we computed motif activity using chromVAR, we observed that several pregranulosa motifs showed high activity in XX precursor cell types, compared to the Sertoli motifs that exhibited increased activity specifically in Sertoli cells (Figs. 5E and 4E). We also observed that several motifs, including RORA, NR2F2, TCF4/12, PBX3, and SOX5, were predicted to be active in Sertoli cells despite the absence of corresponding gene expression (Fig. 5E). Visualizing the motif activity on a UMAP of epithelial, presupporting, Sertoli, and pregranulosa cells revealed distinct patterns: LEF1 and MSX1 showed high activity in epithelial and presupporting cells, RUNX1 exhibited increased activity in presupporting cells, and FOXP1 displayed heightened motif activity in pregranulosa cells (Fig. 5H).

To further investigate the transition from presupporting cells to pregranulosa cells, we examined the changes of DAP numbers between presupporting cells and pregranulosa cells at E11.5 and E12.5. We found minimal differences in chromatin patterns between these two cell types at E11.5 compared to those at E12.5 (Fig. 5I). DAPs acquired in E11.5 pregranulosa cells were associated to *Klf7* and *Sulf1* (data S7). In contrast, a comparison between E11.5 presupporting cells and E12.5 pregranulosa cells revealed significant increased acquisition of pregranulosa-specific chromatin regions (Fig. 5I). These included chromatin regions associated with *Fst*, *Irx3*, *Klf7*, *Lgr5*, *Tcf4*, and *Tcf12* (data S8). Collectively, our analysis identified LEF1 and MSX1 as TF that could be involved in early pregranulosa cell differentiation at E11.5. As the supporting cell differentiation progresses at E12.5, a network of transcription regulators becomes more active in pregranulosa cells, which undergo significant chromatin remodeling at this time.

### Female-biased *Lef1* and *Msx1* regulation during pregranulosa differentiation

**Fig. 6.**
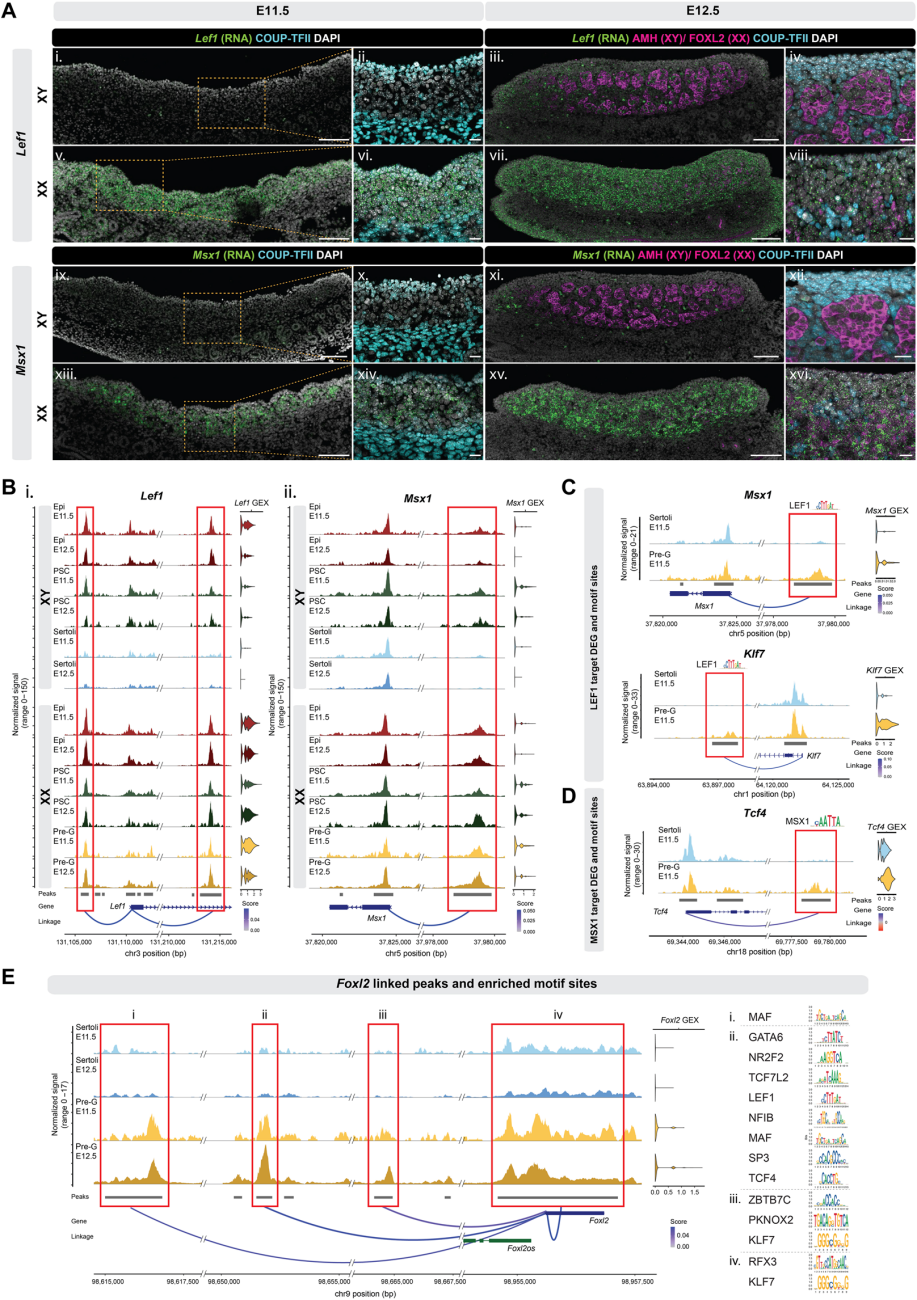
Pregranulosa cell–biased *Lef1* and *Msx1* expression and regulation. (**A**) RNA in situ hybridization (RNAscope) of *Lef1* and *Msx1* (green) combined with immunofluorescence staining of COUP-TFII (cyan, ii, iv, vi, viii, x, xii, xiv, and xvi), AMH (magenta, iii, iv, xi, and xii), and FOXL2 (magenta, vii, viii, xv, and xvi) on E11.5 and E12.5 XX and XY gonadal sections. Scale bar, 100 μm (10× objective, i, iii, v, vii, ix, xi, xiii, and xv) or 20 μm (20× objective, ii, iv, vi, viii, x, xii, xiv, and xvi). (**B**) Peak-gene linkage plots of *Lef1* (i) and *Msx1* (ii) in E11.5 and E12.5 epithelial (Epi), presupporting (PSC), Sertoli, and pregranulosa (pre-G) cells. The red boxes denote called peaks (gray bars) that are linked to gene expression (GEX). Linkage score represents the level of association between chromatin accessibility and gene expression. (**C**) Peak-gene linkage plots of *Msx1* (i) and *Klf7* (ii) in E11.5 Sertoli and pregranulosa cells, with LEF1 motif identified within the linked pregranulosa DAPs (red boxes). (**D**) Peak-gene linkage plot of *TCF4* in E11.5 Sertoli and pregranulosa cells, with MSX1 motif identified within the linked pregranulosa DAPs (red box). (**E**) Peak-gene linkage plots of *Foxl2* in E11.5 and E12.5 Sertoli and pregranulosa cells, with identified motifs enriched within each linked peaks (red boxes, i to iv) shown on the right side. DAPI, 4′,6-diamidino-2-phenylindole.

As LEF1 and MSX1 were identified as potential TFs underlying initial pregranulosa differentiation, we investigated how *Lef1* and *Msx1* were regulated at the transcript level. We first performed RNA in situ hybridization (RNAscope) combined with immunofluorescence of known markers to characterize the cellular localization of *Lef1* and *Msx1* in E11.5 and E12.5 gonads. At E11.5, we observed clear sex differences in *Lef1* and *Msx1* expression: *Lef1* transcripts were abundant in nearly all cell types in the XX gonad, whereas in the XY gonad, *Lef1* was scarcely detected (Fig. 6A, i, ii, v, and vi). Similarly, *Msx1* transcripts were present in most cells in the XX gonad except for the coelomic epithelium, while in the XY gonad, *Msx1* expression was minimal (Fig. 6A, ix, x, xiii, and xiv). At E12.5, *Lef1* remained broadly expressed in almost all cell types in the XX gonad, whereas in the XY gonad, *Lef1* expression was restricted to germ cells and interstitial cells (AMH negative/COUP-TFII–positive cells) (Fig. 6A, iii, iv, vii, and viii). Similarly, *Msx1* showed a broad expression pattern at E12.5 in an XX gonad, while in the XY gonad *Msx1* expression was low and limited to non-Sertoli cells (Fig. 6A, xi, xii, xv, and xvi).

To understand how the sex-dimorphic pattern of *Lef1* and *Msx1* expression is established, we investigated the chromatin regions that were associated with *Lef1* and *Msx1* expression (Fig. 6B). In the *Lef1* locus, two chromatin regions, one upstream of the *Lef1* promoter and one intragenic, were associated with *Lef1* expression (Fig. 6B, i). In the XX gonads, these two regions remained accessible in epithelial, presupporting, and pregranulosa cells. However, in the XY gonads, their accessibility was reduced in E12.5 presupporting cells and in both E11.5 and E12.5 Sertoli cells (Fig. 6B, i). For *Msx1*, we identified one chromatin region upstream of the *Msx1* promoter that was associated with its expression (Fig. 6B, ii). This region also remained accessible in XX epithelial, presupporting, and pregranulosa cells but exhibited reduced accessibility in Sertoli cells (Fig. 6B, ii). The decreased accessibility among these chromatin regions may explain the reduced expression of *Lef1* and *Msx1* in Sertoli cells.

Last, to investigate the potential molecular actions of LEF1 and MSX1 and their downstream regulators, we examined the presence of motifs of all potential regulators identified in Fig. 5E. We first identified a LEF1 motif within accessible regions upstream of the *Msx1* promoter and downstream of *Klf7* promoter. These regions were more accessible in pregranulosa cells than in Sertoli cells (Fig. 6C). Similarly, an MSX1 motif was identified downstream of *Tcf4* promoter within a chromatin region more accessible in pregranulosa cells than Sertoli cells (Fig. 6D). Both KLF7 and TCF4 were among the downstream, secondary factors enriched within E12.5 pregranulosa DEG with linked DAPs (Fig. 5E). Binding motifs of KLF7 and TCF4, along with other TF such as MAF, GATA6, ZBTB7C, and SP3, were mapped to the accessible peaks associated with expression of granulosa cell–specific genes *Foxl2* and *Fst* (Fig. 6E and fig. S6A). For *Foxl2*, four chromatin regions were associated with its expression: the *Foxl2* promoter and three upstream regions (Fig. 6E). Within these regions, the MAF motif was mapped to the first peak (Fig. 6E, i); motifs such as GATA6, LEF1, SP3, and TCF4 were mapped to the second peak (Fig. 6E, ii); ZBTB7C and KLF7 were among the motifs identified in the third peak (Fig. 6E, iii); and RFX3 and KLF7 were mapped to the promoter peak of *Foxl2* (Fig. 6E, iv). For *Fst*, motifs including TCF4/12, KLF7, ZBTB7C, GATA6, and RUNX1 were found within the three linked peaks closest to its promoter that were associated with *Fst* expression (fig. S6A). We also analyzed the chromatin regions linked to the expression of pro-ovarian factors *Wnt4* and *Rspo1*. Enrichment of factors such as MEIS1/2, ZBTB7C, SP3, TCF12, NFIB, and KLF7 were observed within peaks linked to *Wnt4* and *Rspo1* (fig. S6, B and C). These factors, expressed also in XY precursor cell types, may regulate *Wnt4* and *Rspo1* expression in XY precursor cells before the linked chromatin regions become inaccessible in Sertoli cells (fig. S6, B and C). Unlike *Foxl2* and *Fst*, *Wnt4* and *Rspo1* were expressed in both XX and XY precursor cell types (fig. S6, B and C). *Wnt4* was differentially expressed between E11.5 XX and XY coelomic epithelial cells; however, its expression was low in the epithelium (data S3). *Wnt4* was up-regulated in presupporting cells of both sexes, further increasing in pregranulosa cells while decreasing in Sertoli cells. Meanwhile, *Rspo1* was present in E11.5 XX and XY epithelial cells and presupporting cells. Its expression was up-regulated in pregranulosa cells but down-regulated in Sertoli cells, becoming a DEG only at this stage (data S3). Together, these findings suggest a potential mechanism by which LEF1 and MSX1 may initiate a transcription network cascade, eventually leading to up-regulation of downstream secondary factors that collectively facilitate pregranulosa gene expression and differentiation.

### Ex vivo knockdown of *Lef1* disrupts ovarian morphology and FOXL2 expression in E11.5 but not E12.5 ovary

**Fig. 7.**
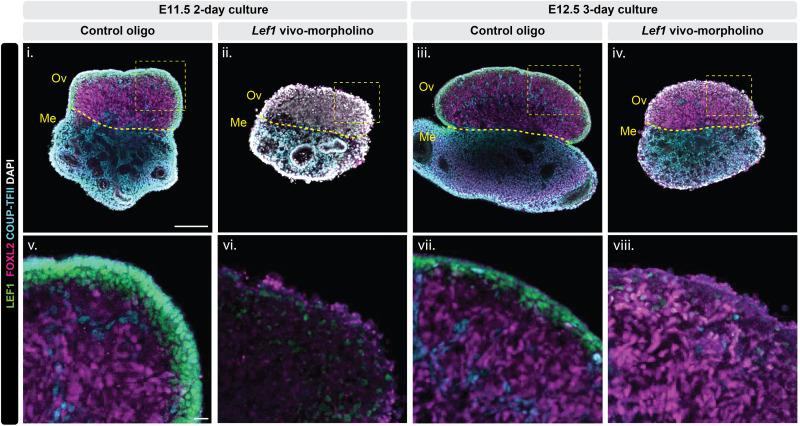
Ex vivo ovarian culture combined with *Lef1* vivo-morpholino treatment disrupts ovarian morphology and FOXL2 expression. E11.5 (i, ii, v, and vi) and E12.5 (iii, iv, vii, and viii) XX gonads with attached mesonephros were cultured for 2 days (E11.5) or 3 days (E12.5) in the presence of either control oligo (i, iii, v, and vii) or *Lef1* vivo-morpholino (ii, iv, vi, and viii). Tissues were then fixed and subjected to whole-mount immunofluorescence staining for LEF1 (green), FOXL2 (magenta), COUP-TFII (cyan), and DAPI (gray). Images show a single z slice from the whole-mount tissue. Scale bars, 100 μm (i to iv); 10 μm (zoomed-in insets, v to viii); Ov, ovary; Me, mesonephros.

To investigate the functional role of the putative transcription regulators such a*s Lef1* during early ovarian development, we applied an ex vivo hanging droplet culture system combined with an antisense knockdown approach using vivo-morpholinos (*37*). Vivo-morpholinos (Gene Tools LLC) consist of morpholino oligonucleotides conjugated to a delivery moiety that facilitates efficient cellular uptake, enabling rapid gene knockdown and short-term functional analysis in tissues. We cultured E11.5 XX gonads with either a standard control oligos or *Lef1*-targeting vivo-morpholinos for 2 days. In control samples, LEF1 proteins were most enriched in and underneath the ovarian epithelium (Fig. 7, i and v). Robust expression of FOXL2 proteins was observed as expected in pregranulosa cells with the COUP-TFII–positive mesenchymal cells scarcely dispersed among granulosa cells and in the mesonephros. In contrast, *Lef1*-morpholino–treated XX gonads were notably smaller and exhibited a marked reduction in LEF1 proteins, accompanied by a decrease of FOXL2 and COUP-TFII expression in the ovaries (Fig. 7, ii and vi). This phenotype was not observed when *Lef1*-morpholino treatment started at E12.5 (Fig. 7, iii and iv). After three days of *Lef1*-morpholino treatment at E12.5, the treated ovaries exhibited reduced LEF1 proteins; however, FOXL2 protein expression was maintained (Fig. 7, iii and iv). These results suggest that *Lef1* knockdown at E11.5 exerts a stronger effect on ovarian cell identity and FOXL2 expression than knockdown at E12.5, indicating a temporal sensitivity to *Lef1* function during early ovary development.

## DISCUSSION

### Validity of single-nucleus multiomics in cell type identification and peak-gene association

Our single-nucleus multiomics data identified all gonadal cell types with cell composition that aligns with published single-cell RNA-seq datasets (*26*, *30*). Compared to single “cell” RNA-seq, expression of certain genes (e.g., *Foxl2* in pregranulosa cells) appeared lower in our single “nucleus” RNA-seq data, likely because only nuclear mRNA was captured using the multiome assay. Nonetheless, the nuclear mRNA may reflect active transcription more closely compared to total mRNA analyzed with single-cell RNA-seq, as nuclear mRNA represents higher proportion of newly transcribed genes (*38*). In setting the parameters for linkage analysis, we noticed that the analysis is sensitive to the composition of the dataset. We obtained the most peak-gene linkages when the analysis was performed on the entire dataset, encompassing all cell types, compared to performing the analysis within individual cell types. Our approach allowed for the identification of all potential regulatory chromatin regions not limited to supporting cells. Nonetheless, the linkage results may differ if the analysis was restricted to Sertoli and pregranulosa cells alone.

### Sex- and cell type–specific chromatin remodeling during gonadal differentiation

We observed that chromatin states correlated with cell type–specific gene expression among major cell types, suggesting the involvement of chromatin remodeling in not only Sertoli and presupporting cell differentiation (*22*, *24*) but also in the emergence of supporting-like cells (*26*) and interstitial cell differentiation. While we observed exceptions to this correlation when comparing directly between RNA and ATAC clusters, we reason that they were due to the resolution of the clustering analysis used. When we explored further into the chromatin states during supporting cell differentiation across time points, we noticed that epithelial and presupporting cells at E11.5 exhibited minimal sex differences, reflecting the bipotential state of somatic precursor cells. The acquisition of Sertoli DAPs readily by E11.5 was driven by an active increase in chromatin accessibility, particularly in distal and intronic regions, as shown by increased accessibility compared with epithelial and presupporting cells. In contrast, the acquisition of pregranulosa DAPs, which occurred predominantly at E12.5, was a result from a combination of Sertoli cells decreasing accessibility and pregranulosa cells gaining accessibility in these regions. These results support the model in which SRY become active in XY supporting cell progenitors around E11.0 (*39*). The action of SRY, its downstream effector SOX9, and other TFs lead to the opening of previously condensed chromatin regions by E11.5. Without SRY and the activation of its downstream pathway, chromatin changes in pregranulosa cells were not observed until later developmental time point, potentially facilitated through other chromatin remodelers unique to the XX environment.

Sertoli and pregranulosa-enriched genes are marked initially as bivalent with both active H3K4me3 and repressive H3K27me3 histone modifications at their promoter regions, and this bivalency is lost after sex determination (*24*). In our analysis, however, we did not observe significant changes in the repressive H3K27me3 modifications within Sertoli and pregranulosa DAPs. We reason that this was due to most DAPs being located within distal intergenic and intragenic regions. Moreover, we found that known bivalent promoters of genes including *Sox9*, *Dmrt1*, and *Fgf9*, were not DAPs between Sertoli and pregranulosa cells and therefore were not included in the analysis. These results provide a glimpse into the dynamic changes in distal regulatory regions, in addition to the action of bivalent promoters, occurring during Sertoli and pregranulosa cell differentiation.

### Sertoli and pregranulosa cells exhibit increased DEG associated with open chromatin during E11.5 to E12.5 transition

We observed that Sertoli cells and pregranulosa cells exhibited the highest level of DEG with linked DAP, reflecting how Sertoli and pregranulosa cells had the highest number of DAPs. While the level of increase in DAPs in Sertoli and pregranulosa cells from E11.5 to E12.5 does not correlate with changes in the number of DEG, this may reflect the ability of multiple DAPs to function as regulatory elements for a single gene. In contrast, most DEGs in other somatic cells between sexes were linked to non-DAP. This suggests that these genes may be regulated independently of chromatin state change. For example, the linked chromatin region is equally accessible in both sexes, while gene expression is regulated by different sex-specific TFs. We also observed that more than 30% of DEGs between Sertoli and pregranulosa cells were not associated with any peaks at all. As the linkage analysis was performed within a distance of TSS ± 500 kb, distal regulatory elements beyond this linkage distance [e.g., Enh13 in Sertoli cells (*40*)] were not considered in the analysis. Therefore, these genes could either be regulated by sex-specific TFs or through distal enhancers beyond the linkage distance, which could be explored in future studies using chromosome conformation capture–based techniques (*41*).

Perhaps most strikingly, we found a large proportion of DEGs with linked non-DAPs at E11.5 converted to DEG with linked DAP at E12.5 in both Sertoli and pregranulosa cells. This pattern may result from a small subset of cells expressing high level of the gene at E11.5, rendering it a DEG, while the chromatin-accessible peaks of these genes in these cells do not reach statistical significance when analyzed in combination with the rest of the cells as a group. By E12.5, most cells increase the accessibility of the associated chromatin regions, or decrease it in the opposite sex, establishing it as a DAP. Alternatively, the DEGs with linked non-DAP at E11.5 may be regulated through mechanisms independent of chromatin accessibility. In either scenario, these dynamic changes in the association between chromatin accessibility and gene expression allowed us to identify computationally the enriched TFs underlying this active transition.

### In silico identification of known and new regulators of Sertoli cell differentiation

Motif scan and linkage analysis revealed that SOX motifs were enriched in E11.5 Sertoli DAPs linked to *Dmrt1* expression, suggesting potential direct regulation of *Dmrt1* through SOX factors at the beginning of Sertoli differentiation. This result aligns with existing knowledge that SOX9 and DMRT1 play critical roles in Sertoli cell specification and fate maintenance (*42*, *43*). Notably, despite the similarity between SOX and DMRT1 motifs, clustering based on downstream-linked genes indicated that these two genes regulate different target genes, also consistent with our understanding of their distinct roles in Sertoli cell function.

At E12.5, we identified other TFs such as NR6A1, THRA, JUN, and IRF2 with high predicted motif activity. NR6A1 shares a similar motif with NR5A1 but exhibited higher gene expression than *Nr5a1* in Sertoli cells, particularly during the E11.5 presupporting to Sertoli transition. Also known as the germ cell nuclear factor, NR6A1 is highly expressed in germ cells (*25*) and is implicated in regulating spermatogenesis and oogenesis (*44*, *45*). Its enrichment and differential expression in Sertoli cells may indicate an additional and yet-to-be-identified role in mediating Sertoli differentiation. Our analysis further suggested that THRA, JUN, and IRF2 (*46*) may regulate early Sertoli differentiation. Sertoli cells express thyroid hormone receptors and respond to thyroid hormone signaling, which drives their proliferation during the neonatal period (*47*). JUN is also expressed in Sertoli cells and may interact with PA1 to regulate junctional protein expression (*48*). Although IRF2 is known for its role in immune regulation and cell growth (*49*, *50*), its specific involvement in Sertoli cell differentiation has yet to be defined.

### Joint transcriptomic and chromatin accessibility assays reveal transcriptional regulatory networks of pregranulosa cell differentiation

We identified LEF1 and MSX1 that could function as upstream regulators of pregranulosa cell differentiation. LEF1 is a member of T cell factor/lymphoid enhancer factor (TCF/LEF) family and acts as a key TF downstream of the WNT signaling pathway (*51*). The pathway, specifically its factors WNT4 and RSPO1, play essential roles in early recruitment of presupporting cells from the epithelium in both sexes in addition to regulating ovarian differentiation (*52*). The fact that the LEF1 motif was already active in epithelial cells, preceding the up-regulation of *Wnt4* in presupporting cells, suggested that it could be regulated by a different WNT factor, such as *Wnt5a* (*53*). Alternatively, LEF1 could be regulated through other pathways or independently (*54*, *55*). The exact mechanism of how *Lef1* is regulated in precursor cell types require further characterization. The LEF1 motif remained active in XX presupporting and pregranulosa cells, while its activity was down-regulated in XY cells. As a member of the LEF/TCF family, LEF1 shares similar motifs with TCF7L1 and TCF7L2, both of which were identified as potential secondary factors in pregranulosa differentiation. As revealed in the ex vivo morpholino experiment, LEF1 appears to be required for early pregranulosa cell differentiation at E11.5, but not beyond E12.5, indicating that *Lef1* may be dispensable for the second wave of pregranulosa cell specification (*12*). Of note, the attempt to knock down *Msx1* was unsuccessful and warrants future investigation. In addition, morpholino treatment affects the whole gonad, and a cell type–specific approach would precisely delineate its role within the supporting cell lineage.

Other than transmitting WNT signaling, a potential action of LEF1 is to directly modulate *Msx1* expression (*56*). *Msx1* encodes a homeodomain TF involved in meiosis initiation (*57*). Double knockout of *Msx1* and *Msx2* exhibited reduced meiotic germ cells in mouse ovaries (*57*). However, its role in somatic cell differentiation in the ovary remains unknown. MSX1 shares similar motif with LHX9 and EMX2, both of which are involved in coelomic epithelial proliferation and early fate establishment (*58*, *59*) and were identified among the secondary factors in our analyses. The expression of *Lhx9* and *Emx2* persisted in XX presupporting cells and pregranulosa cells but was down-regulated in XY cells. In a recent study using bulk RNA and ATAC-seq of purified pregranulosa and Sertoli cells across time points of sex determination, EMX2 and LXH9 also emerged as the most enriched TF motifs during pregranulosa cell differentiation (*60*).

Among the secondary factors, we identified including RUNX1, GATA6, TRPS1, and NF1A/B as TFs with high predicted motif activity in pregranulosa cells. RUNX1 marks presupporting cells in both sexes and then becomes differentially expressed in pregranulosa cells (*16*). Along with FOXL2, RUNX1 maintain pregranulosa identity (*16*). GATA6, a member of the GATA TF family, interact with GATA4 in regulating early folliculogenesis in mice (*61*). While GATA6 and GATA4 share similar motifs, GATA4 was differentially expressed in Sertoli cells and was not identified as an enriched motif in pregranulosa cells. Nevertheless, GATA4 was expressed and predicted to have high motif activity in pregranulosa cells. TRPS1, another member of the GATA TF family, also shares a similar motif with GATA6 and GATA4. While TRPS1 has been associated with high-grade serous ovarian carcinoma (*62*), its role in early ovarian development remains underexplored. Similarly, members of the nuclear factor I family, including NFIA and NFIB, were identified in our analyses as potential secondary regulators. These two factors were implicated in ovarian cancer metastasis and drug resistance (*63*, *64*); however, their roles in ovarian differentiation have not been defined. None of these secondary factors in pregranulosa cells were negatively linked to genes encoding the identified Sertoli TFs, whereas several Sertoli TF motifs were enriched within peaks negatively linked to *Bnc2*, *Zbtb7c*, *Pbx1*, *Tcf4*, and *Gli3*. These observations suggest that Sertoli cells actively suppress pregranulosa fate through negatively regulating the expression of pregranulosa-enriched TFs, but not vice versa.

The identification of the putative TFs is predictive of their potential involvement in transcriptional regulation but does not imply causation. In this study, candidate regulators were limited to TFs. Other factors involved in signaling transduction, posttranslational modifications, or metabolic regulation that are not necessarily TFs were not included in the study. The -KTS isoform of *Wt1* was reported to be essential for pregranulosa cell differentiation (*17*). Without total RNA or long-read sequencing, we were not able to capture and quantify the variant in our dataset. Moreover, *Wt1* was not a DEG in supporting cells between sexes across time points. Last, future analyses could explore the role of molecules secreted by supporting cells in activating pathways that regulate target gene expression in other cell types. Collectively, we present a robust dataset that is particularly valuable for studying transcriptional regulatory networks underlying gonadal cell fate transitions, including the differentiation of Sertoli, pregranulosa, supporting-like cells, and the establishment of interstitial lineage. Our findings not only generate new hypotheses but also identify potential targets for functional studies to understand human DSDs and other reproductive system disorders.

## MATERIALS AND METHODS

### Animals

Twelve- to Sixteen-week-old female mice bred from homozygous Rosa-tdTomato9 females (JAX 007909) [B6.Cg-Gt(ROSA)26Sor < tm9(CAG-tdTomato)Hze>/J] crossed to homozygous Nr5a1-cre males [B6D2-Tg(Nr5a1-cre)2Klp, provided by the late K. Parker (*65*)] were used for the 10x Multiome assay. C57BL/6J (JAX 000664) female mice of the same age were used for RNA in situ hybridization and culture with vivo-morpholino treatment. The day of detection of vaginal plug after time mating was considered E0.5. Food (NIH-31 M, Harlan Teklad) and water were given ad libitum, and the mice were kept in a 12-hour light, 12-hour dark cycle with temperature ranging 21° to 23°C and relative humidity ranging 40 to 50%. All animal studies were conducted in accordance with the NIH Guide for the Care and Use of Laboratory Animals and approved by the National Institute of Environmental Health Sciences (NIEHS) Animal Care and Use Committee (Protocol 2010-0016).

### Gonadal sample collection and preparation

Whole fetal mouse gonads were collected at E11.5, E12.5, and E13.5 in ice-cold phosphate-buffered saline (PBS) using insulin needles under a fluorescence microscope (Leica M165 FC). As tdTomato fluorescence is only present in the gonad but not the mesonephros, it was used to facilitate the dissection of the gonad away from the mesonephros. Tail somite counting was performed on E11.5 embryos, which ranged from 17 to 23 somites. The sexes of E11.5 and E12.5 embryos were determined using amnion staining (*66*) and confirmed with polymerase chain reaction using primers that recognize the Y chromosome. Gonads of the same sex and age were pooled in PBS with 0.4% bovine serum albumin (Sigma-Aldrich) and immediately frozen in liquid nitrogen until nuclei isolation. Nuclei isolation was performed using the 10X Genomics Chromium Nuclei Isolation Kit (catalog no. 1000494) following the manufacturer’s protocol. The total numbers of gonads used per sex and age are listed in table S2.

### Library preparation for snRNA-seq and ATAC-seq

The 10X Genomics Chromium Next GEM Single Cell Multiome ATAC + Gene Expression Library Preparation Kit (catalog no. 1000284) was used to generate a 10X barcoded library of mRNA and transposed DNA from individual nuclei. Libraries were prepared from 3 to 19 biological replicates (pairs of gonads) and 2 technical replicates per embryonic stage and sex (table S2 and Fig. 1, A and B), averaging 8687 nuclei per sample for sequencing with Illumina NOVAseq to a minimum sequencing depth of 129,120,442 raw reads and 22,406 read pairs per nucleus (table S2 and fig. S1, A and B).

### Data preprocessing for snRNA-seq and ATAC-seq libraries

CellRanger-ARC (v3.0) (*67*, *68*) was used for count, alignment to mm10 reference genome, filtering, cell barcode, and UMI counting (table S2 and fig. S1C). Barcode swapping correction was performed for all libraries (*69*). FASTQ files of the corrected cell count matrices were generated, and the data were analyzed using Seurat (v. 4.3.0) (*70*) and Signac (v. 1.9.0) (*71*) in R (v. 4.2.1) with default settings unless otherwise noted. For snRNA-seq preprocessing, doublets within each dataset were removed with the scDblFinder (v. 1.10.0) (*72*) package using default methods. Ambient RNA was removed using the celda/decontX (v. 1.12.0) (*73*) package, with the maximum iterations of the EM algorithm set at 100. Datasets of gonadal cell populations from each embryonic stage and sex were combined into one Seurat object using the “merge” function. As batch effect was not observed in our dataset, we did not perform further integration or batch correction (fig. S1D). A subset of the data was generated using the following cutoffs: nCount_RNA > 1000 & nCount_RNA < 25,000 & percent.mt < 25. The data were normalized using the “SCTransform” function in Seurat. For snATAC-seq data preprocessing, peak calls from CellRanger were used to generate one combined peak set using the function “reduce” and a subset of the data was generated using the following cutoffs: peak size >20 base pairs (bp) and peak size <10,000 bp. Fragment objects were created using the Signac package and combined with the Seurat object. A subset of the snATAC-seq data was generated using the following cutoffs: nCount_ATAC <100,000 & nCount_ATAC >1000 & nucleosome_signal <2 & TSS.enrichment >1.

### Computational sex filtering

We applied computational filters to confirm the sex of the cells in each dataset based on Y-linked gene expression and fragments mapped to the Y chromosome (chrY). Y-linked genes that were expressed in our dataset including *Kdm5d*, *Eif2s3y*, *Uty*, and *Ddx3y*, were used to create a module score using the UCell package (v. 2.0.1) (*74*). XY cut-off value was set at 1 SD below the mean module score of all XY E13.5 samples. Whereas XX cut-off value was set at 1 SD above the mean module score of all XX E13.5 samples. Next, we examined the peaks called on chrY outside of the pseudoautosomal region. In total, 21 peaks were identified between chrY 1 and 90,000,000 bp. E11.5 and E12.5 samples with 0 fragment counts within our defined chrY peak region and Y-linked gene expression module score <1 SD were removed from the “XY” datasets. E11.5 and E12.5 samples with >0 fragment counts within our defined chrY peak region or Y-linked gene expression module score >1 SD were removed from the “XX” datasets.

### Clustering and cell type annotation

We performed linear dimensional reduction using the “RunPCA” function and nonlinear dimensional reduction using the “RunUMAP” function with dims = 1:15 on the scaled snRNA-seq data. Cell clustering was then performed using the “FindNeighbors” function with dims = 1:15 and the “FindClusters” function with resolution = 0.3. This generated in total 14 cell clusters. We combined established marker gene expression, sex, and age composition for cell type annotation. Specifically, primordial germ cell (PGC) cluster is positive for *Ddx4* and *Pou5f1* expression; pregranulosa is an XX cluster and is positive for *Fst* and *Foxl2* (*15*); Sertoli is an XY-only cluster and is positive for *Amh* and *Sox9* (*29*); presupporting cell (PSC) shares similar gene expression with pregranulosa and Sertoli including *Wnt4*, *Wnt6*, *Gata4*, and *Wt1*, and is enriched in both sexes at E11.5 (*16*); supporting-like cells (SLCs) are positive for *Pax8* and *Slc25a21* (*26*); epithelial cluster is positive for *Upk3b* and *Aldh1a*2 (*27*, *28*); Leydig cluster is positive for *Cyp11a1* and *Hsd3b1* and composed of only XY samples; mesenchymal (Me) cluster is positive for *Ptn* and *Nr2f2*, while the expression pattern is similar to IP cluster, IP is more closely related to the Leydig cluster; erythrocyte cluster is positive for *Hba-a1*; immune cluster is positive for *Cx3cr1* and *Fcgr1*; endothelial cluster is positive for *Pecam1* but negative of PGC gene expression; and mesonephric tubule is positive for *Pax2* (*2*, *26*, *30*, *75*).

### Peak calling by cluster and linkage analysis

We performed peak calling using MACS2 (v. 2.2.7.1) (*31*) on individual RNA clusters to obtain cluster-specific peaks. Peaks on nonstandard chromosomes were removed using the “keepStandardChromosomes” function in Signac with the option ‘pruning.mode = “coarse”’ and peaks in mm10 genomic blacklist regions were removed. Counts in each peak were determined using Signac functionalities. The data were normalized by latent semantic indexing (LSI) using the “RunTFIDF,” “FindTopFeatures,” and “RunSVD” functions in Signac. Visualization was performed using UMAP with dims = 2:30, reduction = “lsi,” and algorithm = 3. MACS2 called peaks were linked to scaled gene expression data using the entire dataset with the “LinkPeaks” function in Signac for quantifying the correlation between peak accessibility with gene expression.

### Identification of DEGs and DAPs

DEGs in each cluster were determined using the “FindAllMarkers” function in Seurat based on the Wilcoxon rank sum test with min.pct = 0.25 and logfc.threshold = 0.25. DEGs between male and female somatic clusters at each stage were identified with the “FindMarkers” function in Seurat with min.pct = 0.25 and logfc.threshold = 0.25. DAPs in each cluster were determined using the FindAllMarkers function based on the likelihood-ratio (LR) test with min.pct = 0.05 and logfc.threshold = 0.25. DAPs between male and female somatic clusters at each time point were identified with the FindMarkers function with min.pct = 0.01 and logfc.threshold = 0.1. To annotate linkage between DEGs and DAPs, when at least one DAP is significantly (*P* < 0.05) linked to a DEG, the DEG is annotated DEG with linked DAP; when any of the significantly linked peak to a DEG is not a DAP, the DEG is annotated DEG with linked non-DAP; and when a DEG has no significantly linked peak, it is annotated DEG with no linked peak (data S3). In case of DAP, when at least one DEG is significantly (*P* < 0.05) linked to a DAP, the DAP is annotated “DAP with linked DEG”; when any of the significantly linked gene to a DAP is not a DEG, the DAP is annotated “DAP with linked non-DEG”; and when a DAP has no significantly linked gene it is annotated “DAP with no linked-gene” (data S2).

### Peak annotation and histone mark analysis

Peak annotation was performed using ChIPseeker (v. 1.42.0) (*76*, *77*). To analyze chromatin accessibility within cell type–specific DAPs, function “CountsInRegion” in Signac was applied. To examine histone marks overlapping cell type–specific DAPs, we applied published ChIP-seq datasets of H3K27ac (GSE118755) (*22*), H3K4me3, and H3K27me3 (GSE130749) (*24*) on fluorescence-activated cell–sorted gonadal cells. Genome coordinates were converted from mm9 to mm10 assembly using the UCSC Genome Browser (*78*) LiftOver tool before the analysis.

### TF-binding site motif enrichment analysis

DNA sequence motif analysis of DAPs linked to DEGs was performed using the “FindMotifs” function in Signac. Motif position frequency matrices for vertebrates were obtained from the 2022 JASPAR Core database (*79*) with, in total, 1956 elements. Background peaks were selected to match the GC content in the peak set by using the “AccessiblePeaks,” “GetAssayData,” and “MatchRegionStats” functions in Signac. Enriched motifs were filtered with p.adjust <0.05 and fold.enrichment >1.25. MotifScan (v. 1.3.0) (*35*) was used to determine the genomic position of linked peaks to DEGs to identify predicted target genes of TFs.

### RNA in situ hybridization and immunostaining

Gonads from E11.5 and E12.5 C57BL/6J mice were dissected and fixed in 4% paraformaldehyde overnight at 4°C before being processed for paraffin embedding and sectioning into 5 μm slices with standard protocols. Multiplex fluorescent reagent kit v2 (RNAscope, Advanced Cell Diagnostics) was used for RNA in situ hybridization. All procedures were carried out according to manufacturer’s recommendations. Specifically, RNAscope probes Mm-Lef1 (catalog no. 441861) and Mm-Msx1 (catalog no. 421841, Advanced Cell Diagnostics) were applied. Before 4′,6-diamidino-2-phenylindole (DAPI) staining, the slides were blocked in blocking buffer (5% donkey serum/0.1% Triton X-100 in PBS) for 1 hour at room temperature and counterstained with anti–COUP-TFII (1:200, R&D Systems, catalog no. PP-H7147-00), anti-AMH (1:500, Santa Cruz Biotechnology, catalog no. sc-6886), or anti-FOXL2 (1:200, Novus Biological, catalog no. NB100-1277) antibodies diluted in blocking buffer overnight at 4°C. The next day, the slides were washed and incubated with donkey anti-mouse (1:200, Invitrogen, catalog no. A10037) and donkey anti-goat (1:200, Invitrogen, catalog no. A21447) secondary antibodies diluted in blocking buffer incubation for 1 hour at room temperature. The slides were imaged with a Zeiss LSM 900 confocal microscope using Zen software.

### Embryonic gonad culture with vivo-morpholino treatment

E11.5 and E12.5 XX gonads with attached mesonephros were dissected from C57BL/6J mice and cultured in hanging droplets (*37*) of Dulbecco’s modified Eagle’s medium/F12 (Gibco, catalog no. 21041-025) supplemented with 10% heat-inactivated fetal bovine serum (Gibco, catalog no. 16140071) and 1× penicillin-streptomycin (Sigma-Aldrich, catalog no. P0781). For each embryo, gonads were cultured individually in medium containing either 5 μM standard control vivo-morpholino (Gene Tools, 5′ CCTCTTACCTCAGTTACAATTTATA 3′), 5 μM *Lef1* vivo-morpholino (Gene Tools, combined variants 201, 202, and 204: 5′ GGCTGTGTAATCTCCGCTCCGCTGC 3′ and variant 203: 5′ GGCTTGTCTGACCACCCCTGCCAT 3′), or 15 μM *Msx1* vivo-morpholino (Gene Tools, 5′ GCCATGCAGAGCCGGACCCCCCCTC 3′). Cultures were maintained for 48 hours (E11.5) or 72 hours (E12.5) at 37°C in 5% CO_2_.

### Whole-mount immunofluorescence staining

Cultured gonads with mesonephros were collected and fixed in 4:1 methanol:dimethyl sulfoxide at −20°C for a minimum of 24 hours. Tissues were then washed in 50% methanol in 1× PBS for 30 min at room temperature, followed by three 1-hour washes in blocking buffer (5% donkey serum/0.1% Triton X-100 in PBS). Tissues were incubated overnight at 4°C in blocking buffer containing anti–COUP-TFII (1:200, R&D Systems, catalog no. PP-H7147-00), anti-FOXL2 (1:200, Novus Biological, catalog no. NB100-1277), and anti-LEF1 (1:200, catalog no. PA5-119910) antibodies. The next day, tissues were washed three times for 1 hour each in blocking buffer at 4°C, followed by incubation with donkey anti-mouse (1:200, Invitrogen, catalog no. A10037), donkey anti-goat (1:200, Invitrogen, catalog no. A21447), and donkey anti-rabbit (1:200, Invitrogen, catalog no. A21206) secondary antibodies diluted in blocking buffer incubation for 1 hour at room temperature. Tissues were dehydrated with sequential 1-hour washes in increasing concentrations of methanol (25, 50, 75% with DAPI, and 100%) and then cleared in 1:2 benzyl alcohol:benzyl benzoate for at least 24 hours. Imaging was performed using a Zeiss LSM 900 confocal microscope using Zen software.

### Statistical analyses

No sample size calculation was performed. Statistical analyses were considered significant if *P* < 0.05.

## Supplementary Materials

### The PDF file includes:

Figs. S1 to S10 Tables S1 and S2 Legends for data S1 to S8 Compiled code
