# Supplementary material for: Single-nucleus multiomics of murine gonads reveals transcriptional regulatory network underlying supporting lineage differentiation
Source: Sci Adv. 2026 Jan 2;12(1):eaea7403. doi: 10.1126/sciadv.aea7403 (PMC12758517; doi:10.1126/sciadv.aea7403)
Supplement: Supplementary file 1 — Figs. S1 to S10 Tables S1 and S2 Legends for data S1 to S8 Compiled code [file sciadv.aea7403_sm.pdf]

Science Advances
AAAS

Supplementary Materials for

Single-nucleus multiomics of murine gonads reveals transcriptional regulatory network underlying supporting lineage differentiation

Yu-Ying Chen *et al.*

Corresponding author: Humphrey Hung-Chang Yao, humphrey.yao@nih.gov

Sci. Adv. **12**, eaea7403 (2026)
DOI: 10.1126/sciadv.aea7403

The PDF file includes:

Figs. S1 to S10
Tables S1 and S2
Legends for data S1 to S8
Compiled code

Other Supplementary Material for this manuscript includes the following:

Data S1 to S8

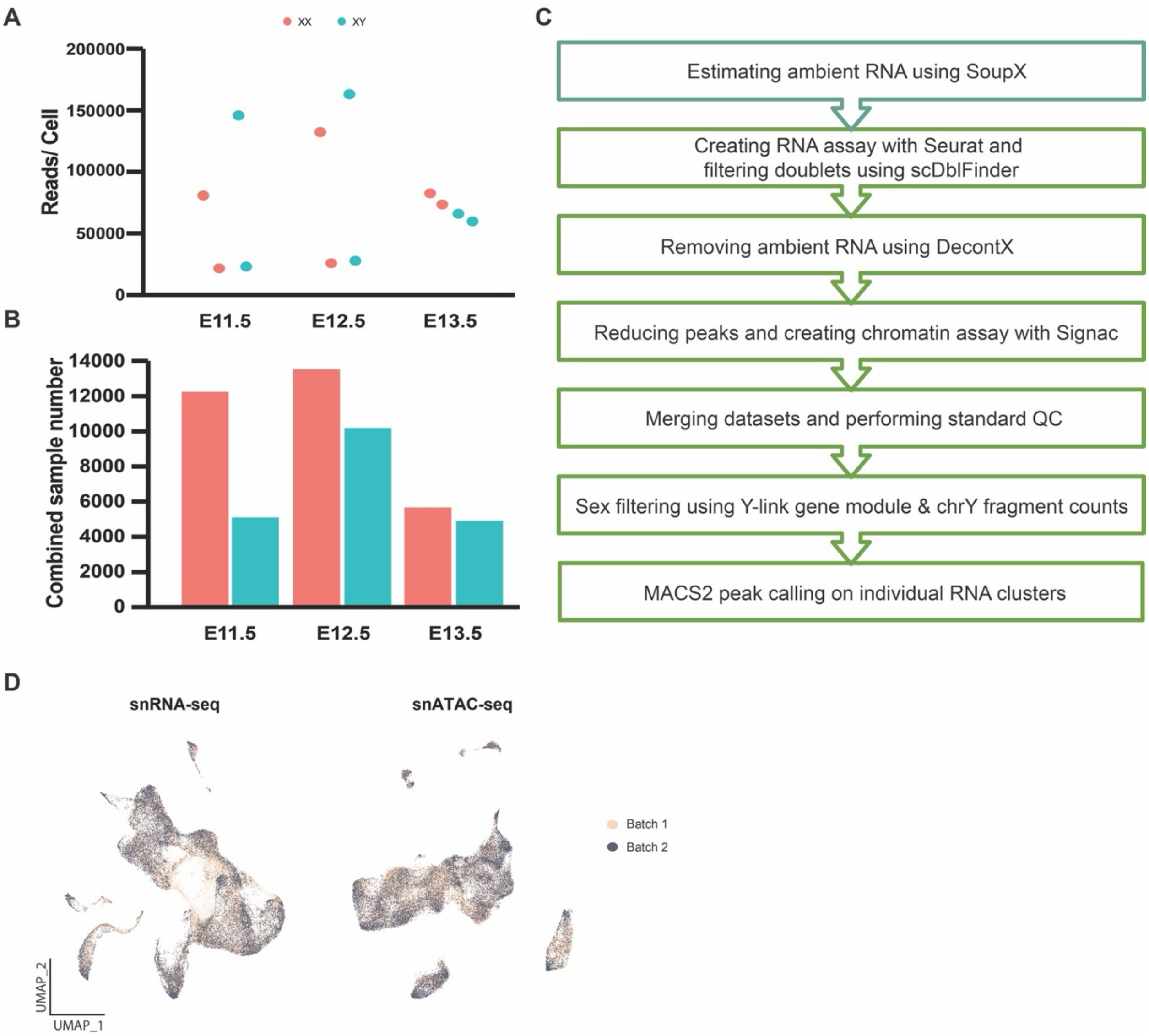

Figure S1. Joint single-nucleus multiomics pre-analysis pipeline. (A-B) Sequencing depth (A) and nuclei number (B) of individual sample. (C) Pre-analysis pipeline, including ambient RNA removal, doublets filtering, sex filtering, and peak calling. (D) UMAP visualization of snRNA-seq and snATAC-seq data, color-coded by batch.

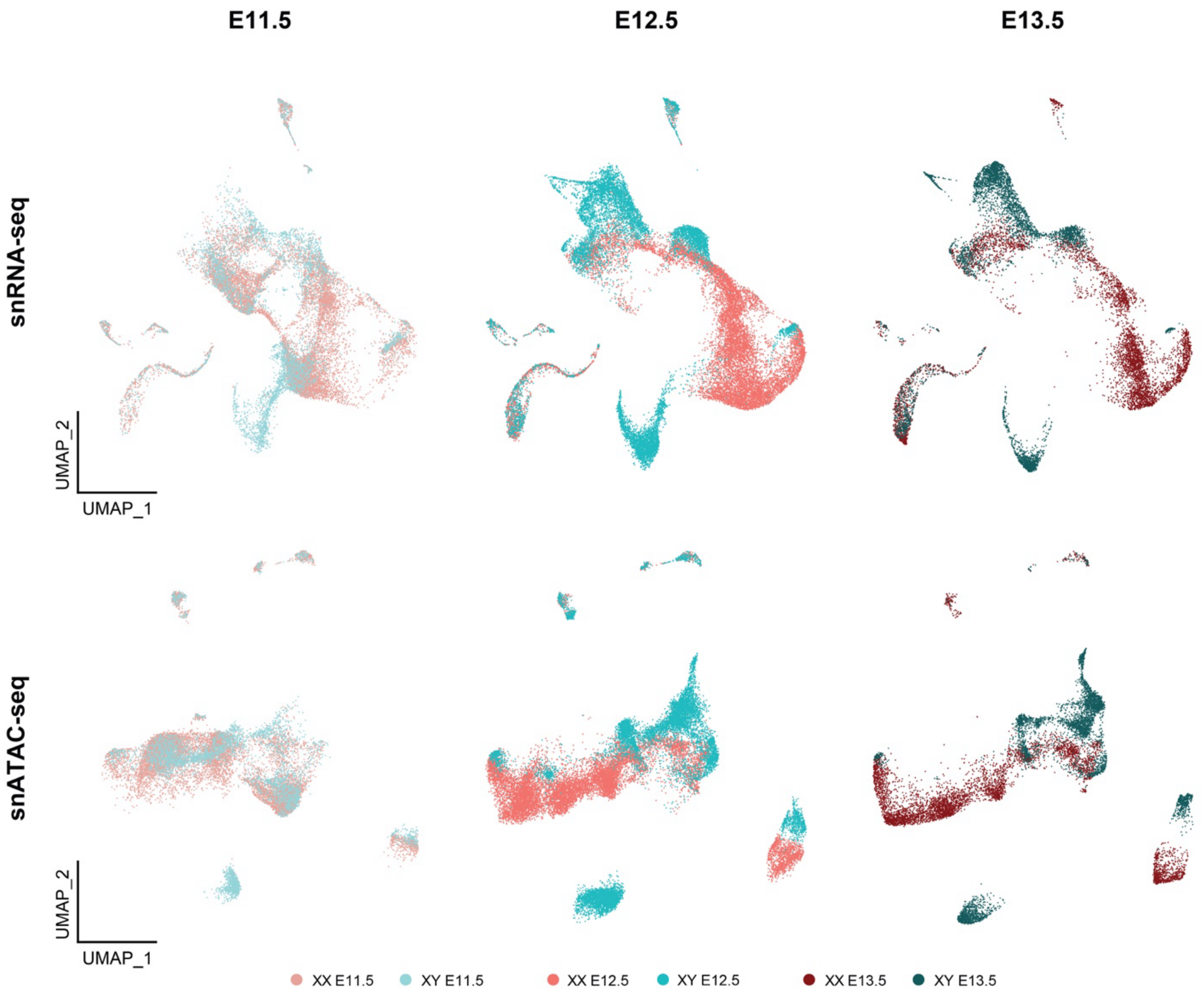

Figure S2. **UMAP of RNA and ATAC profiles across developmental timepoints**. UMAP of snRNA-seq (top) and snATAC-seq (bottom) data of XX and XY gonadal cells color coded by developmental timepoints.

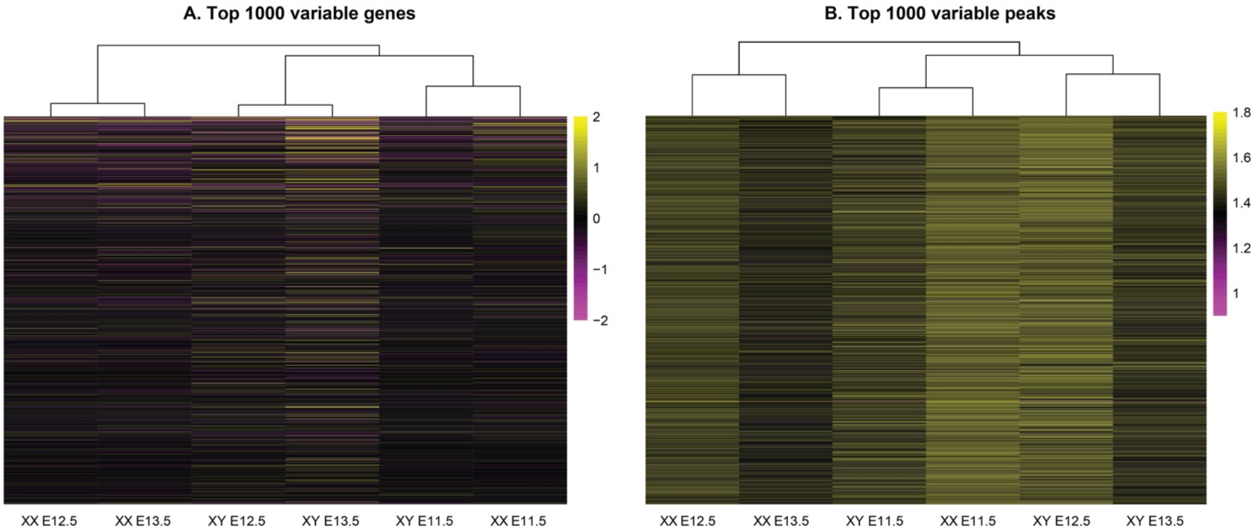

Figure S3. **Heatmaps of average expression and chromatin accessibility across sex and developmental timepoints.** (A) Heatmap of the top 1,000 variable genes, showing average expression for each sex and developmental timepoint group. (B) Heatmap of the top 1,000 variable chromatin accessibility peaks, showing average accessibility per group. Dendrograms were generated using hierarchical clustering based on pairwise Pearson correlation between groups.

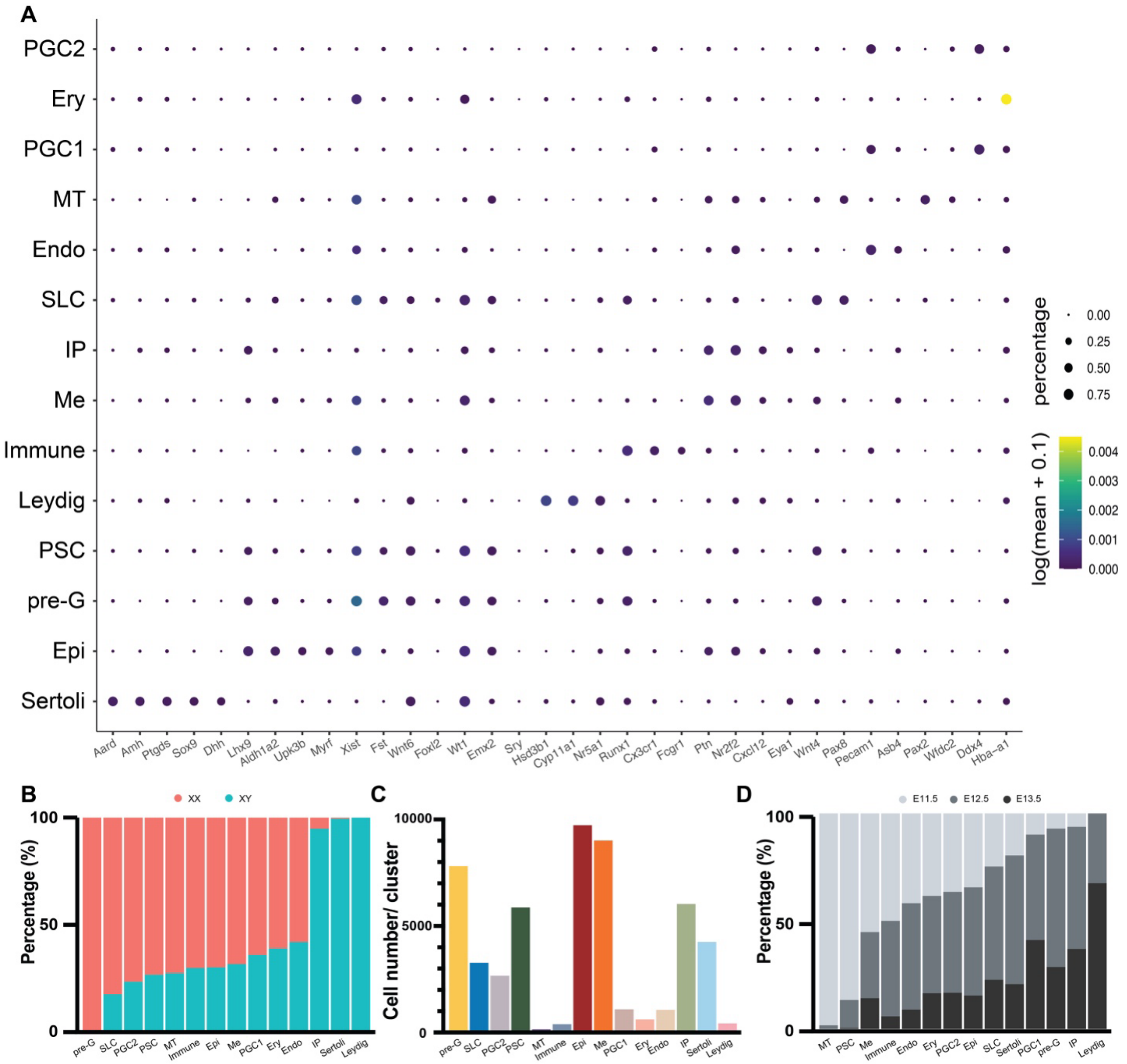

Figure S4. Cell type annotation and distribution. (A) Gene expression profile of the top DEGs identified in each cell type, annotated through RNA clustering. (B) Percentage of cells from each sex within each cell type. (C) Total number of nuclei in each cluster. (D) Percentage of developmental timepoints in each cluster. Annotations for the clusters are: Gonadal epithelial (Epi), Mesenchymal (Me), Pre-granulosa (pre-G), Interstitial progenitor (IP), Pre-supporting (PSC), Supporting-like cell (SLC), Germ cell (PGC), Endothelial cell (Endo), Erythrocyte (Ery), Mesonephric tubule (MT).

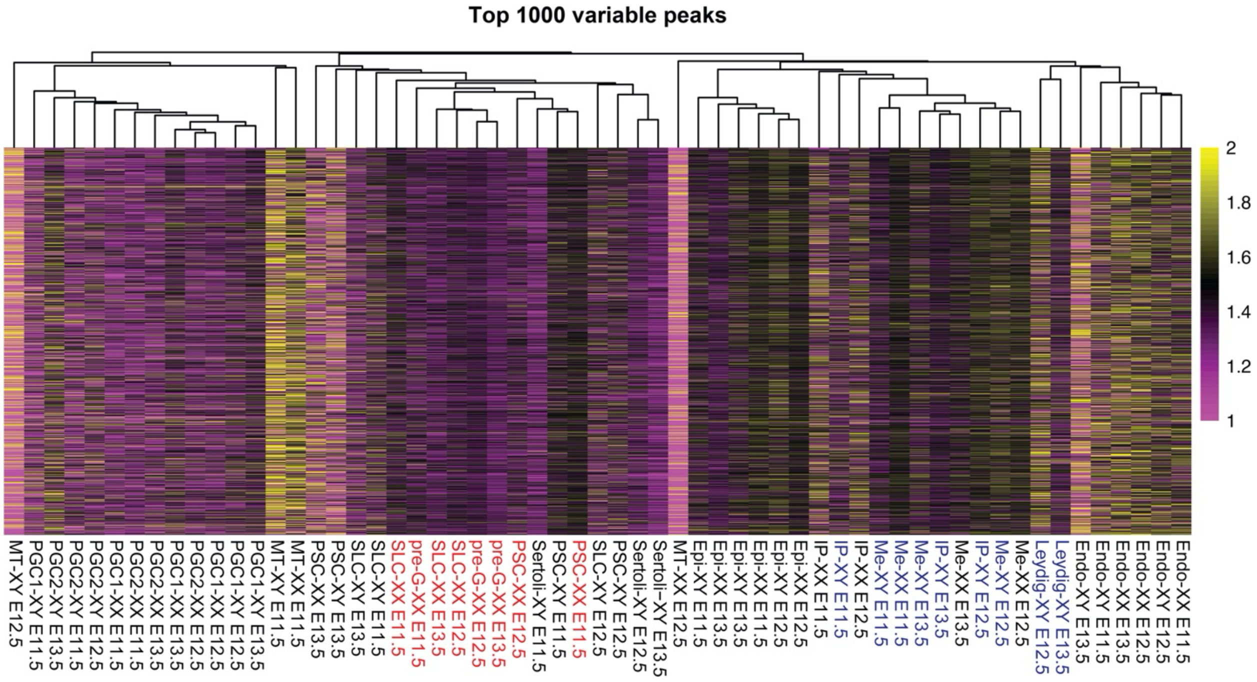

Figure S5. **Heatmap of average chromatin accessibility across cell types**. Heatmap displaying the average accessibility of the top 1,000 variable peaks across individual cell clusters. Dendrograms indicate hierarchical clustering of clusters based on pairwise Pearson correlation of their chromatin accessibility profiles. XX pre-supporting cells (PSC), supporting-like cells (SLC), and pre-granulosa (pre-G) cells are highlighted in red, emphasizing their closely related chromatin pattern. XY Leydig cells, interstitial progenitor cells (IP), and mesenchymal cells (Me) are highlighted in blue.

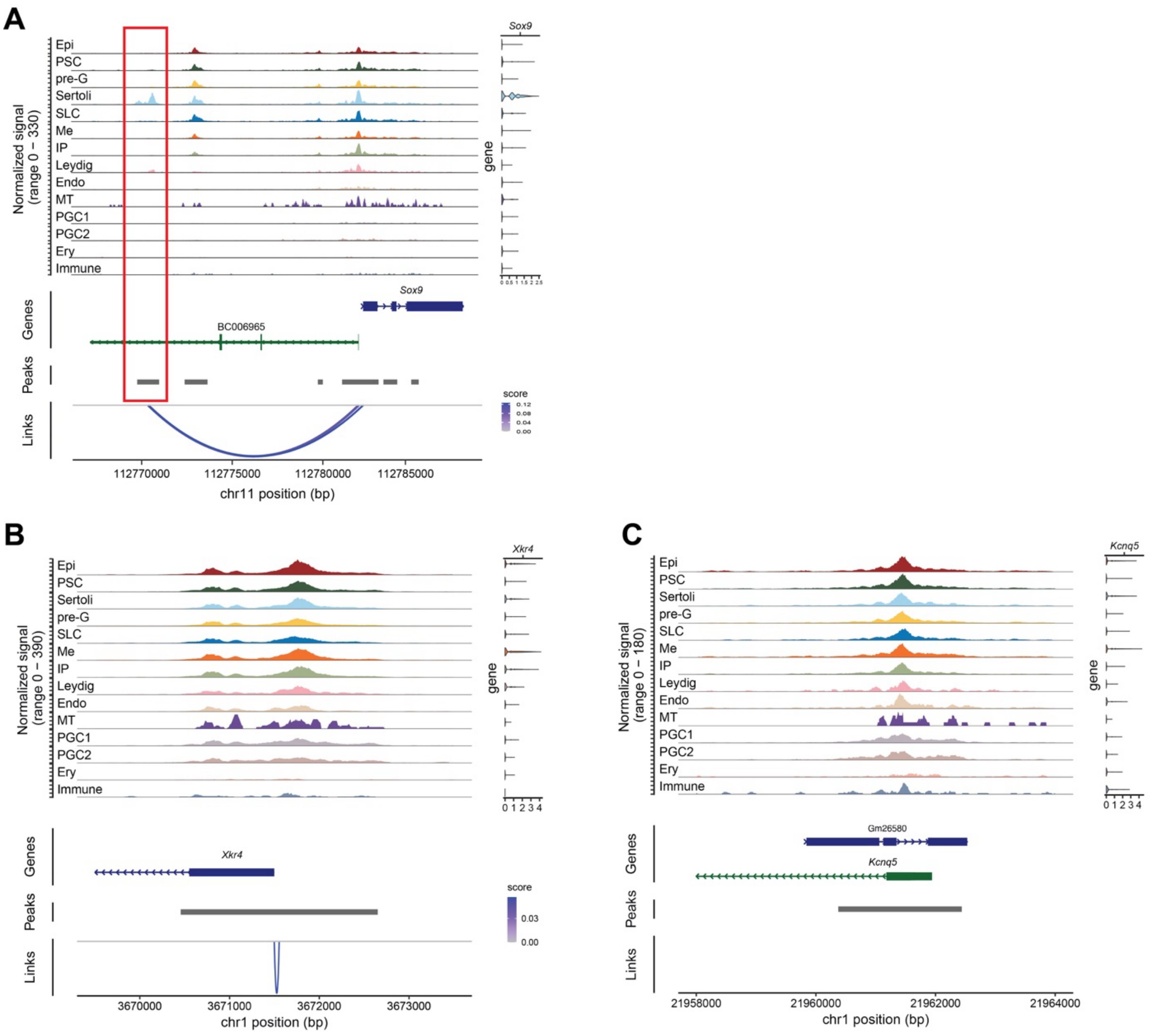

Figure S6. Linkage plot of *Sox9*, *Xkr4*, and *Kcnq5*. (A) Peak-gene linkage plot for *Sox9* across cell types from combined sex and developmental stages. The linkage score represents the level of association between chromatin accessibility and gene expression. The red box denotes the TES enhancer location. (B) Peak-gene linkage plot for *Xkr4* across cell types from combined sex and developmental stages. The linkage score represents the level of association between chromatin accessibility and gene expression. (C) Peak-gene linkage plot for *Kcnq5* across cell types from combined sex and developmental stages, with no linkage identified. Annotations for the clusters are: Gonadal epithelial (Epi), Pre-supporting (PSC), Pre-granulosa (pre-G), Supporting-like cell (SLC), Mesenchymal (Me), Interstitial progenitor (IP), Endothelial cell (Endo), Mesonephric tubule (MT), Germ cell (PGC), Erythrocyte (Ery).

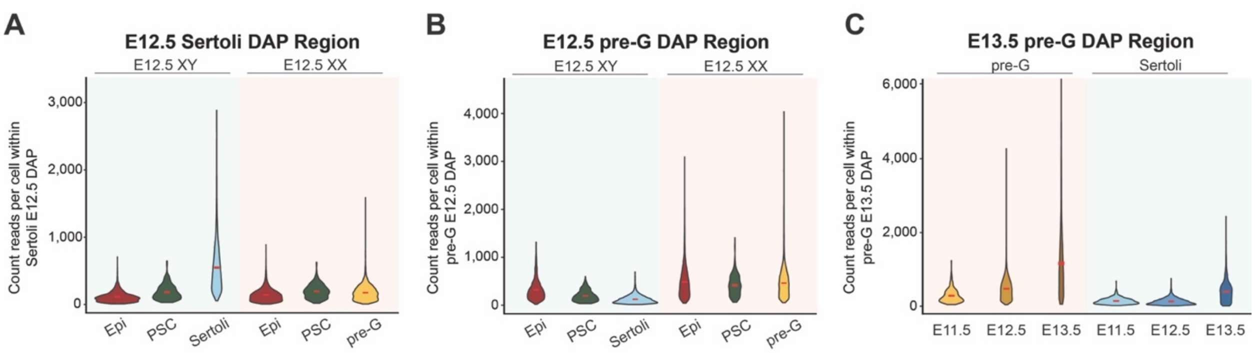

Figure S7. Chromatin accessibility of Sertoli and pre-granulosa DAPs in precursor and differentiated supporting cells. (A) Chromatin accessibility, measured by total count reads per cell, within E12.5 Sertoli DAP regions in E12.5 XY and XX epithelium (Epi), pre-supporting cells (PSC), Sertoli, and pre-granulosa (pre-G) cells. (B) Total count reads per cell within E12.5 pre-granulosa DAP region in E12.5 XY and XX epithelium, pre-supporting, Sertoli, and pre-granulosa cells. (C) Total count reads per cell within E13.5 pre-granulosa DAP regions (10,124 unique peaks) in pre-supporting and Sertoli cells throughout developmental timepoints.

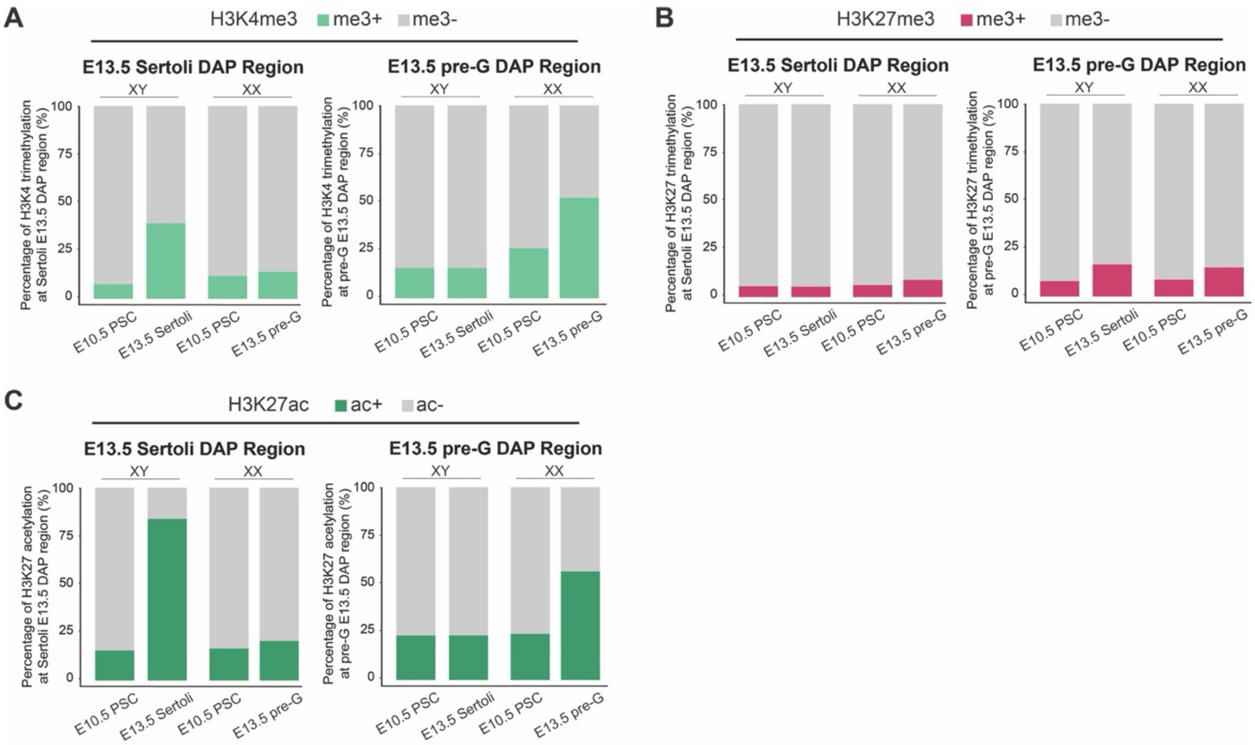

Figure S8. Changes in histone modifications within E13.5 Sertoli and pre-granulosa DAPs. (A-C) Percentage of chromatin regions positive for H3K4me3 (A), H3K27me3 (B), and H3K27ac (C), based on published ChIP-seq datasets (Garcia-Moreno et al, 2019 (*22*, *24*)), overlapping E13.5 Sertoli and pre-granulosa DAPs in XY and XX pre-supporting, Sertoli or pre-granulosa cells.

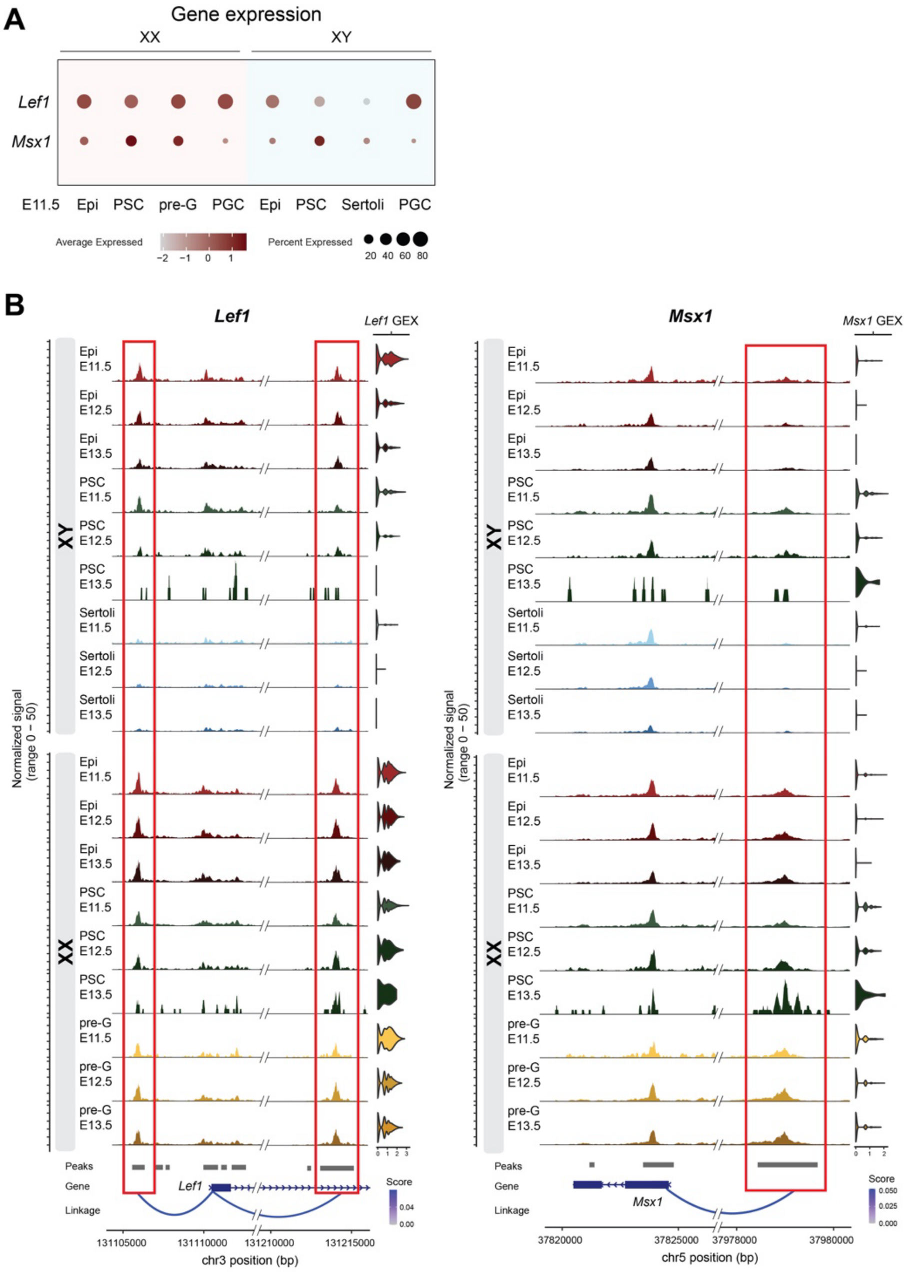

Figure S9. *Lef1* and *Msx1* expression and regulation (A) Gene expression of *Lef1* and *Msx1* in E11.5 XX and XY epithelial (Epi), pre-supporting (PSC), pre-granulosa (pre-G), Sertoli, and germ cells (PGC). (B) Peak-gene linkage plots of *Lef1* and *Msx1* in E11.5, E12.5, and E13.5 epithelial (Epi), pre-supporting (PSC), Sertoli, and pre-granulosa (pre-G) cells. The red boxes denote called peaks (grey bars) that are linked to gene expression (GEX). Linkage score represents the level of association between chromatin accessibility and gene expression.

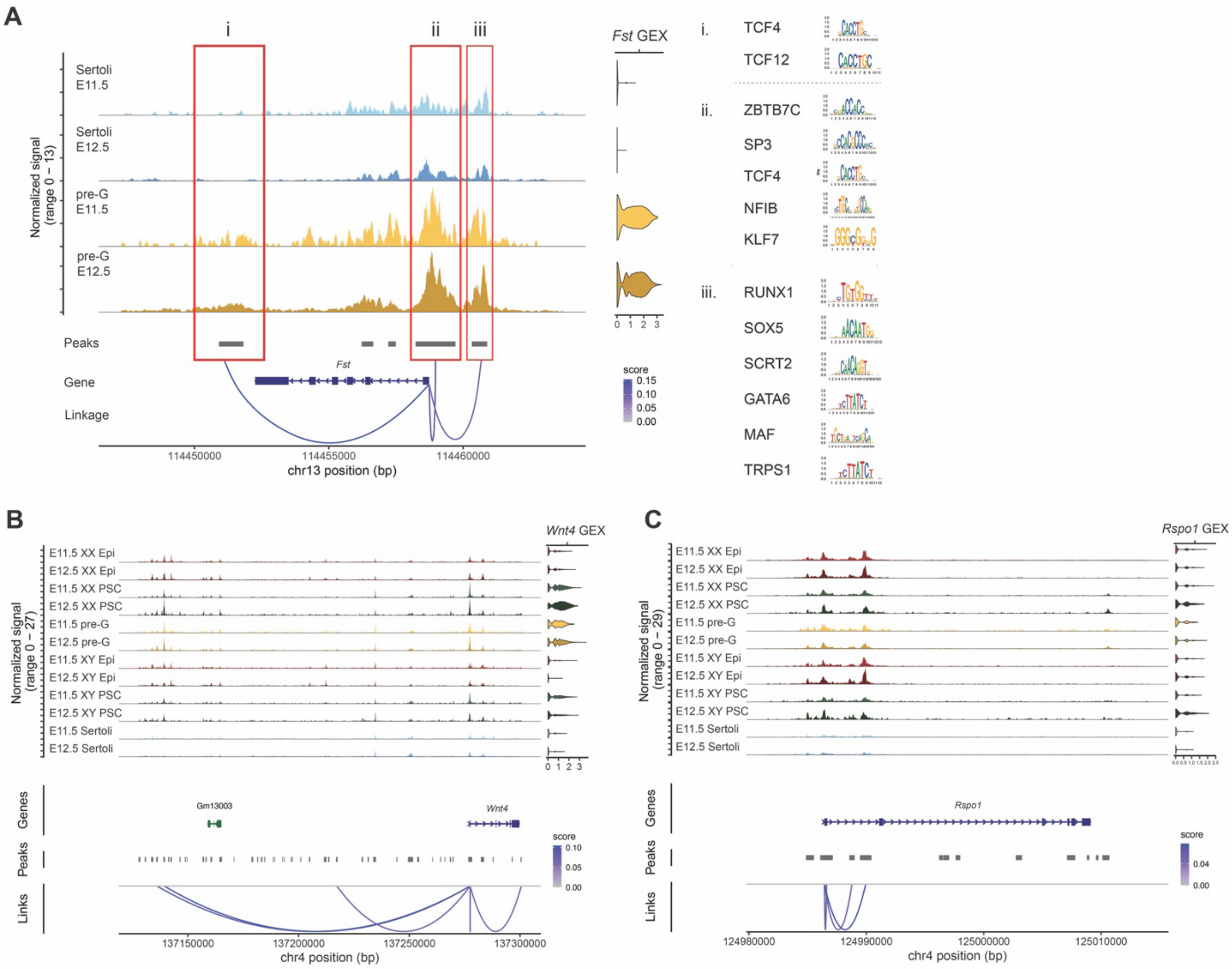

Figure S10. Linkage plots of *Fst*, *Wnt4*, and *Rspo*. (A) Pre-granulosa TF motifs identified within each peak (i-iii) that are associated with *Fst* expression in E11.5 and E12.5 Sertoli and pre-granulosa cells. (B) Linkage plot of *Wnt4* in E11.5 and E12.5 XX and XY epithelial (Epi), pre-supporting (PSC), Sertoli, and pre-granulosa (pre-G) cells. (C) Linkage plot of *Rspo* in E11.5 and E12.5 XX and XY epithelial (Epi), pre-supporting (PSC), Sertoli, and pre-granulosa (pre-G) cells.

Cluster	Eday	Peak	Linked gene	Annotation
Sertoli	E11	chr9-59695229-59695863	Gramd2	DAP with linked non-DEG
Sertoli	E11	chr7-44670363-44671587	Myh14	DAP with linked non-DEG
Sertoli	E11	chr7-44670363-44671587	Rpl13a	DAP with linked non-DEG
Sertoli	E11	chr3-37722511-37724791	*NA*	DAP with no linked-gene
Sertoli	E11	chr16-35588615-35589204	*NA*	DAP with no linked-gene
Sertoli	E11	chr18-36328107-36329238	*NA*	DAP with no linked-gene
Sertoli	E11	chr2-170469006-170470315	*NA*	DAP with no linked-gene
Sertoli	E11	chr4-154961862-154962366	*NA*	DAP with no linked-gene
Sertoli	E11	chr9-66806423-66807130	*NA*	DAP with no linked-gene
pre-G	E11	chr8-87197764-87198797	Cbln1	DAP with linked non-DEG
pre-G	E11	chr5-91073594-91074965	Ereg	DAP with linked DEG
pre-G	E11	chr2-33129634-33132046	Rpl12	DAP with linked non-DEG
pre-G	E11	chr2-70471756-70472505	Sp5	DAP with linked non-DEG
pre-G	E11	chr2-70474040-70475365	Sp5	DAP with linked non-DEG
pre-G	E11	chr11-61673713-61675259	*NA*	DAP with no linked-gene
pre-G	E11	chr1-135018767-135019938	*NA*	DAP with no linked-gene
pre-G	E11	chr1-194353930-194355618	*NA*	DAP with no linked-gene
pre-G	E11	chr18-80991922-80993331	*NA*	DAP with no linked-gene
pre-G	E11	chr19-45293178-45294088	*NA*	DAP with no linked-gene
pre-G	E11	chr6-99984117-99986211	*NA*	DAP with no linked-gene
pre-G	E11	chr5-37966855-37968907	*NA*	DAP with no linked-gene
pre-G	E11	chrX-13279917-13282249	*NA*	DAP with no linked-gene

Table S1. Bivalent peaks within Sertoli and pre-granulosa E11.5 DAP regions. E11.5 Sertoli cell (Sertoli Cluster) or pre-granulosa cell (pre-G Cluster) specific DAPs (Peak) that are bivalent for both H3K4me3 and H3K27me3 histone marks. The table includes their associated genes (Linked gene) and whether these genes are differentially expressed between E11.5 Sertoli and E11.5 pre-granulosa cells (DEG versus non-DEG). Referenced in Figure 2D.

Sample	#Biol. Rep.	Cells	Median genes/ cell	Raw Reads (GEX)	Barcode (GEX)	Saturation (GEX)	Reads/ cell	Median UMI/cell	Mapped (genome)	Mapped (intron)	Median high-quality fragments/ cell	Raw Reads (ATAC)	Barcode (ATAC)	Saturation (ATAC)
E11.5F_R1	19	20,000	2,030	448,115,792	93%	38%	22,406	3,665	91%	44%	7,104	402,448,500	93%	15%
E11.5F_R2	11	10,008	2,798	812,813,546	94%	68%	81,216	8,184	92%	36%	4,222	133,021,685	90%	13%
E11.5M_R1	18	8,080	1,708	193,672,880	93%	35%	23,969	2,790	91%	40%	5,163	165,335,643	89%	13%
E11.5M_R2	12	4,949	3,512	719,219,983	94%	64%	145,326	9,027	91%	34%	9,407	141,108,889	89%	13%
E12.5F_R1	12	18,696	2,422	498,266,895	93%	43%	26,651	4,620	91%	47%	10,034	490,217,426	92%	18%
E12.5F_R2	11	4,960	3,504	654,449,771	94%	66%	131,946	9,194	91%	35%	11,056	141,606,518	87%	15%
E12.5M_R1	8	14,897	2,682	425,422,729	93%	44%	28,558	5,268	91%	45%	6,433	246,823,770	89%	15%
E12.5M_R2	10	5,864	3,446	951,070,062	94%	74%	162,188	8,351	92%	31%	9,194	138,507,635	88%	15%
E13.5F_R1	3	2,040	2,121	135,990,358	95%	86%	66,662	3,608	92%	39%	33,720	430,635,859	99%	69%
E13.5F_R2	5	6,597	2,969	398,709,188	94%	71%	60,438	6,546	92%	52%	21,061	381,257,018	97%	41%
E13.5M_R1	3	1,555	2,642	129,120,442	94%	82%	83,036	5,132	91%	46%	41,691	367,207,783	99%	72%
E13.5M_R2	7	6,369	2,831	472,180,945	94%	73%	74,137	6,013	91%	42%	23,400	420,734,940	97%	45%

Table S2. Sequencing and QC statistics. Sequencing and statistics in RNA (GEX) and ATAC QC for single-nucleus multiome libraries of E11.5-E13.5 XX (F) and XY (M) gonads, including 3-19 biological replicates (pairs of gonads) and two technical replicates (R1 and R2).

Data S1. (separate file)
Linkage analysis of peak-gene association across the entire dataset. The "Score" represents the statistics of the association, while the "zscore" indicates whether a peak is positively or negatively associated with gene expression.

Data S2. (separate file)
Differential accessible peaks (DAPs) of each cell type compared between sexes (cells.1= XY cells, cells.2= XX cells) at each timepoint. In this analysis, Sertoli cells were directly compared to pre-granulosa cells.

Data S3. (separate file)
Differentially expressed genes (DEGs) of each cell type compared between sexes (cells.1= XY cells, cells.2= XX cells) at each timepoint. Sertoli cells were directly compared to pre-granulosa cells.

Data S4. (separate file)
Motif enrichment analysis performed on E11.5 Sertoli "DEG with linked-DAP", newly formed E12.5 Sertoli "DEG with linked-DAP", E11.5 pre-granulosa cells "DEG with linked-DAP", and newly formed E12.5 pre-granulosa "DEG with linked-DAP".

Data S5. (separate file)
Differential accessible peaks (DAPs) comparing E11.5 XY pre-supporting cells (PSC) (cells.1) with E11.5 Sertoli cells (cells.2).

Data S6. (separate file)
Differentially expressed genes (DEGs) comparing E11.5 XY pre-supporting cells (PSC) (cells.1) with E11.5 Sertoli cells (cells.2).

Data S7. (separate file)
Differential accessible peaks (DAPs) comparing E11.5 XX pre-supporting cells (PSC) (cells.1) with E11.5 pre-granulosa cells (cells.2).

Data S8. (separate file)
Differential accessible peaks (DAPs) comparing E11.5 XX pre-supporting cells (PSC) (cells.1) with E12.5 pre-granulosa cells (cells.2).

Single-nucleus multiomics of murine gonads reveals transcriptional regulatory network underlying supporting lineage differentiation: Compiled code

Yu-Ying Chen

Samples

	Male_batch1	Male_batch2	Female_batch1	Female_batch2
E11.5	NOV0336-KR2-e11-Male	NOV0395-KR5-E11-Male	NOV0336-KR1-e11-Female	NOV0395-KR6-E11-Female
E12.5	NOV0336-KR4-e12-Male	NOV0395-KR3-E12-Male	NOV0336-KR3-e12-Female	NOV0395-KR4-E12-Female
E13.5	NOV0278-WT-E13-Male	NOV0351-KR1-Male	NOV0278-WT-E13-Female	NOV0351-KR2-Female

Data pre-processing

Follow CellRanger-ARC tutorial for alignment and counting reads: https://www.10xgenomics.com/support/software/cell-ranger-arc/latest/analysis. tdTomato-WPRE sequence (https://www.addgene.org/22799/sequences/) can be added to the ref genome, however the counts are low. JAX line info: https://www.jax.org/strain/007909

```
library(EnsDb.Mmusculus.v79)
library(BSgenome.Mmusculus.UCSC.mm10)
library(Signac)
library(Seurat)
library(GenomeInfoDb)
library(GenomicRanges)
library(BiocSingular)
library(scDblFinder)
library(SoupX)
library(celda)
library(UCell)
library(plyranges)
library(dittoSeq)
library(ChIPseeker)
library(TxDb.Mmusculus.UCSC.mm10.knownGene)
library(ggplot2)
library(patchwork)
```

```r
library(dplyr)
library(tidyverse)
library(clustree)
library(reshape2)
library(caret)
library(ggsankey)
library(ggalluvial)
library(motifmatchr)
library(JASPAR2022)
library(TFBSTools)
library(genomation)

set.seed(1234)
```

Estimate ambient RNA level using SoupX directly from CellRanger output folder. SoupX may over-correct data, switch to decontX for cleanup

```r
for (path in c(
  "/yourpath/NOV0395-Yao-Rodriguez/CellRanger/KR5-E11-Male/outs",
  #0.12
  "/yourpath/NOV0395-Yao-Rodriguez/CellRanger/KR6-E11-Female/outs",
  #0.15
  "/yourpath/NOV0395-Yao-Rodriguez/CellRanger/KR3-E12-Male/outs",
  #0.08
  "/yourpath/NOV0395-Yao-Rodriguez/CellRanger/KR4-E12-Female/outs",
  #0.07
  "/yourpath/NOV0351-Yao-Rodriguez/CellRanger/KR1-Male/outs",
  #0.04
  "/yourpath/NOV0351-Yao-Rodriguez/CellRanger/KR2-Female/outs",
  #0.10
  "/yourpath/NOV0336-Yao-Rodriguez/CellRanger/KR2-e11-Male/outs",
  #0.16
  "/yourpath/NOV0336-Yao-Rodriguez/CellRanger/KR1-e11-Female/outs",
  #0.32
  "/yourpath/NOV0336-Yao-Rodriguez/CellRanger/KR4-e12-Male/outs",
  #0.09
  "/yourpath/NOV0336-Yao-Rodriguez/CellRanger/KR3-e12-Female/outs",
  #0.24
  "/yourpath/NOV0278-Yao-Rodriguez-multiome-e13/WT-E13-Male/outs",
  #0.04
  "/yourpath/NOV0278-Yao-Rodriguez-multiome-e13/WT-E13-Female/outs"
)) {
  #0.16
  sc <- load10X(path, includeFeatures = c('Gene Expression')) #takes raw and filtered
feature matrix the same time
  readC <- read.delim(
    paste0(path, "/analysis/clustering/gex/graphclust/clusters.csv"),
    header = TRUE,
    sep = ","
  )
  sc <- setClusters(sc, setNames(readC$Cluster, readC$Barcode))
  sc = autoEstCont(sc)
  assign(path, sc)
}
```

Reduce peaks from all files and generate a common peak set for creating fragment objects

```
peaks.M111 <- read.table(file =
"/yourpath/NOV0336-Yao-Rodriguez/CellRanger/KR2-e11-Male/outs/atac_peaks.bed",
                         col.names = c("chr", "start", "end"))
gr.M111 <- makeGRangesFromDataFrame(peaks.M111)

peaks.M112 <- read.table(file =
"/yourpath/NOV0395-Yao-Rodriguez/CellRanger/KR5-E11-Male/outs/atac_peaks.bed",
                         col.names = c("chr", "start", "end"))
gr.M112 <- makeGRangesFromDataFrame(peaks.M112)

peaks.M121 <- read.table(file =
"/yourpath/NOV0336-Yao-Rodriguez/CellRanger/KR4-e12-Male/outs/atac_peaks.bed",
                         col.names = c("chr", "start", "end"))
gr.M121 <- makeGRangesFromDataFrame(peaks.M121)

peaks.M122 <- read.table(file =
"/yourpath/NOV0395-Yao-Rodriguez/CellRanger/KR3-E12-Male/outs/atac_peaks.bed",
                         col.names = c("chr", "start", "end"))
gr.M122 <- makeGRangesFromDataFrame(peaks.M122)

peaks.M131 <- read.table(file =
"/yourpath/NOV0278-Yao-Rodriguez-multiome-e13/WT-E13-Male/outs/atac_peaks.bed",
                         col.names = c("chr", "start", "end"))
gr.M131 <- makeGRangesFromDataFrame(peaks.M131)

peaks.M132 <- read.table(file =
"/yourpath/NOV0351-Yao-Rodriguez/CellRanger/KR1-Male/outs/atac_peaks.bed",
                         col.names = c("chr", "start", "end"))
gr.M132 <- makeGRangesFromDataFrame(peaks.M132)

peaks.F111 <- read.table(file =
"/yourpath/NOV0336-Yao-Rodriguez/CellRanger/KR1-e11-Female/outs/atac_peaks.bed",
                         col.names = c("chr", "start", "end"))
gr.F111 <- makeGRangesFromDataFrame(peaks.F111)

peaks.F112 <- read.table(file =
"/yourpath/NOV0395-Yao-Rodriguez/CellRanger/KR6-E11-Female/outs/atac_peaks.bed",
                         col.names = c("chr", "start", "end"))
gr.F112 <- makeGRangesFromDataFrame(peaks.F112)

peaks.F121 <- read.table(file =
"/yourpath/NOV0336-Yao-Rodriguez/CellRanger/KR3-e12-Female/outs/atac_peaks.bed",
                         col.names = c("chr", "start", "end"))
gr.F121 <- makeGRangesFromDataFrame(peaks.F121)

peaks.F122 <- read.table(file =
"/yourpath/NOV0395-Yao-Rodriguez/CellRanger/KR4-E12-Female/outs/atac_peaks.bed",
                         col.names = c("chr", "start", "end"))
gr.F122 <- makeGRangesFromDataFrame(peaks.F122)

peaks.F131 <- read.table(file =
"/yourpath/NOV0278-Yao-Rodriguez-multiome-e13/WT-E13-Female/outs/atac_peaks.bed",
                         col.names = c("chr", "start", "end"))
```

```r
gr.F131 <- makeGRangesFromDataFrame(peaks.F131)

peaks.F132 <- read.table(file =
"/yourpath/NOV0351-Yao-Rodriguez/CellRanger/KR2-Female/outs/atac_peaks.bed",
                         col.names = c("chr", "start", "end"))
gr.F132 <- makeGRangesFromDataFrame(peaks.F132)

peaks.uni <- reduce(
  x = c(
    gr.M111,
    gr.M112,
    gr.M121,
    gr.M122,
    gr.M131,
    gr.M132,
    gr.F111,
    gr.F112,
    gr.F121,
    gr.F122,
    gr.F131,
    gr.F132
  )
)
peaksUse <- peaks.uni[width(peaks.uni) < 10000 &
                        width(peaks.uni) > 20]
```

From CellRanger output folders create Seurat objects, remove doublets using scDblFinder, run DecontX and write new Seurat objects. Create fragment objects and add the chromatin assay to the decont Seurat file

```r
for (path in c(
  "/yourpath/NOV0395-Yao-Rodriguez/CellRanger/KR5-E11-Male/outs",
  "/yourpath/NOV0395-Yao-Rodriguez/CellRanger/KR6-E11-Female/outs",
  "/yourpath/NOV0395-Yao-Rodriguez/CellRanger/KR3-E12-Male/outs",
  "/yourpath/NOV0395-Yao-Rodriguez/CellRanger/KR4-E12-Female/outs",
  "/yourpath/NOV0351-Yao-Rodriguez/CellRanger/KR1-Male/outs",
  "/yourpath/NOV0351-Yao-Rodriguez/CellRanger/KR2-Female/outs",
  "/yourpath/NOV0336-Yao-Rodriguez/CellRanger/KR2-e11-Male/outs",
  "/yourpath/NOV0336-Yao-Rodriguez/CellRanger/KR1-e11-Female/outs",
  "/yourpath/NOV0336-Yao-Rodriguez/CellRanger/KR4-e12-Male/outs",
  "/yourpath/NOV0336-Yao-Rodriguez/CellRanger/KR3-e12-Female/outs",
  "/yourpath/NOV0278-Yao-Rodriguez-multiome-e13/WT-E13-Male/outs",
  "/yourpath/NOV0278-Yao-Rodriguez-multiome-e13/WT-E13-Female/outs"
)) {
  seurat_data <- Read10X_h5(paste0(path, "/filtered_feature_bc_matrix.h5"))
  seurat_obj <- CreateSeuratObject(counts = seurat_data$`Gene Expression`, assay = "RNA")
  dbl.des <- computeDoubletDensity(seurat_obj@assays$RNA@counts)
  dbl.calls <- doubletThresholding(data.frame(score = dbl.des),
                                   method = "griffiths",
                                   returnType = "call")
  seurat_obj$DoubletCall <- dbl.calls
  seurat_obj_filtered <- subset(seurat_obj, DoubletCall == 'singlet')
  mat <- as.matrix(seurat_obj_filtered@assays$RNA@counts)
  storage.mode(mat) <- "integer"
  counts_decont <- decontX(x = mat, z = NULL, maxIter = 100)
```

```r
  seurat_obj_decont <- CreateSeuratObject(
    counts = round(counts_decont$decontXcounts),
    min.features = 1,
    min.cells = 1,
    assay = "RNA"
  )
  frags <- CreateFragmentObject(paste0(path, "/atac_fragments.tsv.gz"))
  fragcounts <- FeatureMatrix(
    fragments = frags,
    features = peaksUse,
    cells = colnames(seurat_obj_decont)
  )
  seurat_obj_decont[["ATAC"]] <- CreateChromatinAssay(
    fragcounts,
    fragments = frags,
    sep = c(":", "-"),
    annotation = annotation
  )
  saveRDS(seurat_obj_decont, file = gsub("/yourpath/", "", gsub(
    "/outs",
    ".decont.rds",
    gsub(
      "Yao-Rodriguez/CellRanger/",
      "",
      gsub("Yao-Rodriguez-multiome-e13/", "", path)
    )
  )))
  assign(path, seurat_obj_decont)
}
```

Merging objects

```r
Batch_merged_M11_decont <- merge(
  `NOV0336-KR2-e11-Male.decont`,
  `NOV0395-KR5-E11-Male.decont`,
  add.cell.ids = c("old", "new"),
  project = "BatchMerge"
)
Batch_merged_M12_decont <- merge(
  `NOV0336-KR4-e12-Male.decont`,
  `NOV0395-KR3-E12-Male.decont`,
  add.cell.ids = c("old", "new"),
  project = "BatchMerge"
)
Batch_merged_M13_decont <- merge(
  `NOV0278-WT-E13-Male.decont`,
  `NOV0351-KR1-Male.decont`,
  add.cell.ids = c("old", "new"),
  project = "BatchMerge"
)
Batch_merged_F11_decont <- merge(
  `NOV0336-KR1-e11-Female.decont`,
  `NOV0395-KR6-E11-Female.decont`,
  add.cell.ids = c("old", "new"),
```

```r
  project = "BatchMerge"
)
Batch_merged_F12_decont <- merge(
  `NOV0336-KR3-e12-Female.decont`,
  `NOV0395-KR4-E12-Female.decont`,
  add.cell.ids = c("old", "new"),
  project = "BatchMerge"
)
Batch_merged_F13_decont <- merge(
  `NOV0278-WT-E13-Female.decont`,
  `NOV0351-KR2-Female.decont`,
  add.cell.ids = c("old", "new"),
  project = "BatchMerge"
)

Batch_merged_M11_decont$Eday <- 'E11.5'
Batch_merged_M11_decont$SexEday <- 'Male11.5'
Batch_merged_M12_decont$Eday <- 'E12.5'
Batch_merged_M12_decont$SexEday <- 'Male12.5'
Batch_merged_M13_decont$Eday <- 'E13.5'
Batch_merged_M13_decont$SexEday <- 'Male13.5'
Batch_merged_F11_decont$Eday <- 'E11.5'
Batch_merged_F11_decont$SexEday <- 'Female11.5'
Batch_merged_F12_decont$Eday <- 'E12.5'
Batch_merged_F12_decont$SexEday <- 'Female12.5'
Batch_merged_F13_decont$Eday <- 'E13.5'
Batch_merged_F13_decont$SexEday <- 'Female13.5'

Batch_merged_Male_decont <- merge(
  `Batch_merged_M11_decont`,
  y = list(`Batch_merged_M12_decont`, `Batch_merged_M13_decont`),
  add.cell.ids = c("M11", "M12", "M13"),
  project = "MergeMale"
)
Batch_merged_Female_decont <- merge(
  `Batch_merged_F11_decont`,
  y = list(`Batch_merged_F12_decont`, `Batch_merged_F13_decont`),
  add.cell.ids = c("F11", "F12", "F13"),
  project = "MergeFemale"
)
Batch_merged_Male_decont$Sex <- "Male"
Batch_merged_Female_decont$Sex <- "Female"
All_decont <- merge(
  Batch_merged_Male_decont,
  Batch_merged_Female_decont,
  add.cell.ids = c("M", "F"),
  project = "MergeAll"
)
batch1 <- grep("old", colnames(All_decont), value = TRUE)
All_decont$batch <- ifelse(colnames(All_decont) %in% batch1, "batch1", "batch2")
```

Standard QC

```
All_decont[["percent.mt"]] <- PercentageFeatureSet(All_decont, pattern = "^mt-")
DefaultAssay(All_decont) <- "ATAC"
All_decont <- NucleosomeSignal(All_decont)
All_decont <- TSSEnrichment(All_decont)
All_decont <- subset(
  x = All_decont,
  subset = nCount_RNA < 25000 &
    nCount_RNA > 1000 &
    percent.mt < 25 &
    nCount_ATAC < 100000 &
    nCount_ATAC > 1000 &
    nucleosome_signal < 2 &
    TSS.enrichment > 1
)
saveRDS(All_decont, file = "All_decont_filtered.rds")
```

Sex filtering: combine both Y-linked gene expression (RNA) using UCell and chrY peaks (ATAC) to filter cells of the wrong sex. For RNA filtering, expressed Y-linked genes were picked from all candidates (Prssly, Zfy1, Uba1y, Kdm5d, Eif2s3y, Uty, Ddx3y, Usp9y, Zfy2, H2al2y, Sry, Rbmy) for creating a module score

```
#RNA:
ylink_genelist <- list(c('Kdm5d', 'Eif2s3y', 'Uty', 'Ddx3y'))
DefaultAssay(All_decont_filtered) <- "RNA"
All_decont_filtered <- AddModuleScore_UCell(All_decont_filtered, features =
ylink_genelist, name = "ylink_module")
ylink_score <- FetchData(All_decont_filtered, vars = "signature_1ylink_module")
sampletype <- FetchData(All_decont_filtered, vars = "SexEday")
sex <- FetchData(All_decont_filtered, vars = "Sex")
ylink_score <- cbind(ylink_score, sampletype, sex)
M13score <- ylink_score[ylink_score$SexEday == 'Male13.5', ]
#avg y-gene expression in male e13.5 samples, in which we were 100% certain of the sex.
used to filter male cells with lower than mean-1sd expression
F13score <- ylink_score[ylink_score$SexEday == 'Female13.5', ]
#avg y-gene expression in female e13.5 samples, in which we were 100% certain of the sex.
used to filter female cells with values above mean+1sd expression

#ATAC
DefaultAssay(All_decont_filtered) <- "ATAC"
gr <- granges(All_decont_filtered)
gr_chrY <- gr %>% filter(seqnames == "chrY", start > 1, end < 90000000) #Pseudoautosomal
region, PAR:> 90000000 -> GRanges object with 21 ranges
All_decont_filtered$chrY_counts <- CountsInRegion(All_decont_filtered, assay = "ATAC",
regions = gr_chrY) #this looks at frags within called peak regions

Idents(All_decont_filtered) <- "Sex"
All_decont_sexed <- subset(
  x = All_decont_filtered,
  idents = "Male",
  invert = TRUE,
  subset = chrY_counts == 0 &
    signature_1ylink_module < (
      mean(M13score$signature_1ylink_module) - sd(M13score$signature_1ylink_module)
    )
)
```

```
All_decont_sexed <- subset(
  x = All_decont_sexed,
  idents = "Female",
  invert = TRUE,
  subset = chrY_counts > 0 |
    signature_1ylink_module > (
      mean(F13score$signature_1ylink_module) + sd(F13score$signature_1ylink_module)
    )
)
saveRDS(All_decont_sexed, file = "All_decont_sexed.rds")
```

Figure 1- UMAP, clustering, and linkage analyses

RNA UMAP and cluster analysis

```
DefaultAssay(All_decont_sexed) <- "RNA"
All_decont_sexed <- SCTransform(All_decont_sexed, verbose = TRUE)
All_decont_sexed <- RunPCA(All_decont_sexed)
pct <- All_decont_sexed@reductions$pca@stdev / sum(All_decont_sexed@reductions$pca@stdev)
*100 #define the pc to use for umap
co2 <- sort(which((pct[1:length(pct) - 1] - pct[2:length(pct)]) > 0.1), decreasing =
TRUE)[1] + 1
All_decont_sexed <- RunUMAP(All_decont_sexed, reduction = "pca", dims = 1:co2,
reduction.name = "umap.rna")
All_decont_sexed <- FindNeighbors(All_decont_sexed, reduction = "pca", dims = 1:co2)
All_decont_sexed <- FindClusters(All_decont_sexed, resolution = 0.3)
DimPlot(
  All_decont_sexed,
  reduction = "umap.rna",
  group.by = "SexEday",
  label = TRUE,
  label.size = 4,
  cols = c(
    "#E7A39C",
    "#F8766D",
    "#880F06",
    "#92DBDD",
    "#00BFC4",
    "#0A5A5C"
  )
)
DotPlot(
  All_decont_sexed,
  features = c(
    "Ddx4",
    "Sox9",
    "Amh",
    "Ptgds",
    "Aard",
    "Dhh",
    "Fst",
    "Wnt6",
```

```
    "Wnt4",
    "Foxl2",
    "Hba-a1",
    "Cxcl12",
    "Cx3cr1",
    "Fcgr1",
    "Pax2",
    "Cyp11a1",
    "Hsd3b1",
    "Lhx9",
    "Wt1",
    "Runx1",
    "Upk3b",
    "Pecam1",
    "Nr2f2",
    "Ptn",
    "Nr5a1",
    "Aldh1a2",
    "Pax8"
  ),
  cols = c("lightgrey", "#6A040F")
)
Idents(All_decont_sexed) <- "SCT_snn_res.0.3"
DimPlot(
  All_decont_sexed,
  reduction = "umap.rna",
  label = FALSE,
  label.size = 4,
  cols = c(
    "#EF833E",
    "#F9D564",
    "#80C8AB",
    "#F4AF4D",
    "#A1D3E9",
    "#478547",
    "#6AB759",
    "#342C8B",
    "#2F6AAD",
    "#8750A1",
    "#E265A8",
    "#EC5237",
    "#A43772",
    "#5F1B1D"
  )
)
All_decont_sexed$cluster.SexEday <- paste(Idents(All_decont_sexed),
All_decont_sexed$SexEday, sep = "_")
```

Peak calling by RNA clusters for cluster-specific peaks

```
annotation <- GetGRangesFromEnsDb(ensdb = EnsDb.Mmusculus.v79)
seqlevelsStyle(annotation) <- "UCSC"
genome(annotation) <- "mm10"
```

```r
DefaultAssay(All_decont_sexed) <- "ATAC"
peaks_all <- CallPeaks(All_decont_sexed, group.by = "SCT_snn_res.0.3", macs2.path =
"/yourpath/macs2")
peaks_all <- keepStandardChromosomes(peaks_all, pruning.mode = "coarse")
peaks_all <- subsetByOverlaps(x = peaks_all, ranges = blacklist_mm10, invert = TRUE)
macs2_counts_all <- FeatureMatrix(
  fragments = Fragments(All_decont_sexed),
  features = peaks_all,
  cells = colnames(All_decont_sexed)
)
All_decont_sexed[["peaksbyc"]] <- CreateChromatinAssay(
  counts = macs2_counts_all,
  fragments = Fragments(All_decont_sexed),
  annotation = annotation
)
#peaksbyc: peaks called by RNA clusters
saveRDS(All_decont_sexed, file = "All_decont_sexed.rds")
```

UMAP and cluster analysis using the ATAC assay

```r
DefaultAssay(All_decont_sexed) <- "peaksbyc"
All_decont_sexed <- FindTopFeatures(All_decont_sexed, min.cutoff = 5)
All_decont_sexed <- RunTFIDF(All_decont_sexed)
All_decont_sexed <- RunSVD(All_decont_sexed)
All_decont_sexed <- RunUMAP(
  object = All_decont_sexed,
  reduction = 'lsi',
  dims = 2:30,
  reduction.name = "umap.peaksbyc"
)
All_decont_sexed <- FindNeighbors(object = All_decont_sexed,
                                  reduction = 'lsi',
                                  dims = 2:30)
All_decont_sexed <- FindClusters(
  object = All_decont_sexed,
  verbose = TRUE,
  algorithm = 3,
  resolution = 0.3
)
DimPlot(
  All_decont_sexed,
  reduction = "umap.peaksbyc",
  label = TRUE,
  label.size = 4,
  cols = c(
    "#A1D3E9",
    "#66ccff",
    "#6AB759",
    "#006699",
    "#669966",
    "#336633",
    "#99cc99",
    "#f6973e",
    "#fbd164",
```

```
    "#5f3785",
    "#954034",
    "#EC5237",
    "#e67faf",
    "#9999cc"
  )
) #colored by RNA cluster

Idents(All_decont_sexed) <- "peaksbyc_snn_res.0.3"
DimPlot(
  All_decont_sexed,
  reduction = "umap.peaksbyc",
  group.by = "SexEday",
  label = FALSE,
  label.size = 4,
  cols = c(
    "#E7A39C",
    "#F8766D",
    "#880F06",
    "#92DBDD",
    "#00BFC4",
    "#0A5A5C"
  )
)
```

Making probtable comparing RNA and ATAC clusters

```
p <- as.data.frame(prop.table(
  table(
    All_decont_sexed$SCT_snn_res.0.3,
    All_decont_sexed$peaksbyc_snn_res.0.3
  )
))
#the whole table adds up as 1, convert to each row or column adds up as 1
RNA_cluster <- p %>% group_by(Var1) %>% mutate(Percentage = Freq / sum(Freq))
ATAC_cluster <- p %>% group_by(Var2) %>% mutate(Percentage = Freq / sum(Freq))
ggplot(RNA_cluster, aes(Var2, Var1, fill = Percentage)) +
  geom_tile() +
  scale_fill_gradient(low = "white", high = "#4E4B58")
RNA_cluster$Var2 <- factor(RNA_cluster$Var2 , levels = c(0, 1, 2, 3, 4, 5, 6, 7, 8, 9,
10, 11))
RNA_cluster$Var1 <- factor(RNA_cluster$Var1 , levels = c(0, 2, 4, 3, 13, 5, 1, 7, 8, 6,
9, 10, 12, 11))

ggplot(ATAC_cluster, aes(Var2, Var1, fill = Percentage)) +
  geom_tile() +
  scale_fill_gradient(low = "white", high = "#4E4B58")
ATAC_cluster$Var2 <- factor(ATAC_cluster$Var2 , levels = c(0, 1, 2, 3, 4, 5, 6, 7, 8, 9,
10, 11))
ATAC_cluster$Var1 <- factor(ATAC_cluster$Var1 , levels = c(0, 2, 4, 3, 13, 5, 1, 7, 8, 6,
9, 10, 12, 11))
```

Barplots of celltypes and sexeday composition

```r
dittoBarPlot(
  object = All_decont_sexed,
  var = "Sex",
  group.by = "SCT_snn_res.0.3",
  color.panel = c("#F8766D", "#00BFC4"),
  x.reorder = c(7, 11, 13, 9, 6, 1, 5, 2, 12, 3, 14, 8, 10, 4)
)

dittoBarPlot(
  object = All_decont_sexed,
  var = "Eday",
  group.by = "SCT_snn_res.0.3",
  color.panel = c("#CED4DA", "#6C757D", "#333333"),
  x.reorder = c(6, 9, 2, 5, 14, 3, 13, 1, 11, 10, 12, 7, 8, 4)
)

dittoBarPlot(
  object = All_decont_sexed,
  var = "SexEday",
  group.by = "SCT_snn_res.0.3",
  color.panel = c(
    "#E7A39C",
    "#F8766D",
    "#880F06",
    "#92DBDD",
    "#00BFC4",
    "#0A5A5C"
  ),
  x.reorder = c(7, 11, 13, 9, 6, 1, 5, 2, 12, 3, 14, 8, 10, 4)
)

dittoBarPlot(
  object = All_decont_sexed,
  var = "SCT_snn_res.0.3",
  group.by = "SexEday",
  var.labels.reorder = c(1, 2, 7, 8, 9, 10, 11, 12, 13, 14, 3, 4, 5, 6)
)
```

Peak-gene linkage and coverage plots

```r
DefaultAssay(All_decont_sexed) <- "peaksbyc"
# first compute the GC content for each peak
All_decont_sexed <- RegionStats(All_decont_sexed, genome = BSgenome.Mmusculus.UCSC.mm10)
All_decont_sexed <- LinkPeaks(object = All_decont_sexed,
                              peak.assay = "peaksbyc",
                              expression.assay = "SCT")
saveRDS(All_decont_sexed, file = "All_decont_sexed.rds")

linkspeak_whole <- as.data.frame(All_decont_sexed@assays$peaksbyc@links) #save a separate
linkage file for merging datasets
write.csv(linkspeak_whole, "linkspeak_whole.csv")

CoveragePlot(
  object = All_decont_sexed,
```

```
  region = c("chr13-114450000-114462000"),
  features = "Fst",
  expression.assay = "SCT",
  idents = c("0", "4", "2", "5")
)

CoveragePlot(
  object = All_decont_sexed,
  features = "Amh",
  expression.assay = "SCT",
  idents = c("0", "4", "2", "5"),
  region = c("chr10-80803500-80811000")
)
```

Figure 2- Overlapping differentially accessible peaks with histone marks

Cluster DAP between sexes

```
#DAP comparison b/t same cell types of male and female
DefaultAssay(All_decont_sexed) <- "peaksbyc"
Idents(All_decont_sexed) <- "cluster.SexEday"

output <- "All_decont_sexed_E11DAP"
for (i in 0:13) {
  try({
    ident1 <- paste0(i, "_Male11.5")
    ident2 <- paste0(i, "_Female11.5")
    condition.diffgenes <- FindMarkers(
      All_decont_sexed,
      ident.1 = ident1,
      ident.2 = ident2,
      logfc.threshold = .1,
      min.pct = 0.01,
      test.use = 'LR',
      latent.vars = 'nCount_peaksbyc'
    )
    write.csv(condition.diffgenes, file = paste0(output, i, ".csv"))
  })
}

output <- "All_decont_sexed_E12DAP"
for (i in 0:13) {
  try({
    ident1 <- paste0(i, "_Male12.5")
    ident2 <- paste0(i, "_Female12.5")
    condition.diffgenes <- FindMarkers(
      All_decont_sexed,
      ident.1 = ident1,
      ident.2 = ident2,
      logfc.threshold = .1,
      min.pct = 0.01,
      test.use = 'LR',
      latent.vars = 'nCount_peaksbyc'
```

```
    )
    write.csv(condition.diffgenes, file = paste0(output, i, ".csv"))
  })
}

output <- "All_decont_sexed_E13DAP"
for (i in 0:13) {
  try({
    ident1 <- paste0(i, "_Male13.5")
    ident2 <- paste0(i, "_Female13.5")
    condition.diffgenes <- FindMarkers(
      All_decont_sexed,
      ident.1 = ident1,
      ident.2 = ident2,
      logfc.threshold = .1,
      min.pct = 0.01,
      test.use = 'LR',
      latent.vars = 'nCount_peaksbyc'
    )
    write.csv(condition.diffgenes, file = paste0(output, i, ".csv"))
  })
}

#DAP comparison b/t Sertoli (cluster 5) and pre-G (cluster 2)
E11 <- FindMarkers(
  All_decont_sexed,
  ident.1 = "5_Male11.5",
  ident.2 = "2_Female11.5",
  logfc.threshold = .1,
  min.pct = 0.01,
  test.use = 'LR',
  latent.vars = 'nCount_peaksbyc'
)
E12 <- FindMarkers(
  All_decont_sexed,
  ident.1 = "5_Male12.5",
  ident.2 = "2_Female12.5",
  logfc.threshold = .1,
  min.pct = 0.01,
  test.use = 'LR',
  latent.vars = 'nCount_peaksbyc'
)
E13 <- FindMarkers(
  All_decont_sexed,
  ident.1 = "5_Male13.5",
  ident.2 = "2_Female13.5",
  logfc.threshold = .1,
  min.pct = 0.01,
  test.use = 'LR',
  latent.vars = 'nCount_peaksbyc'
)

write.csv(E11, "All_decont_sexed_E11DAP5_2.csv")
write.csv(E12, "All_decont_sexed_E12DAP5_2.csv")
```

```r
write.csv(E13, "All_decont_sexed_E13DAP5_2.csv")

#loop through the results and extract male and female specific DAPs
fnames = str_remove(list.files(pattern = ".csv"), ".csv")
all <- bind_rows(lapply(fnames, function(fname) {
  read.csv(paste0(fname, ".csv")) %>% mutate(grp = fname)
}))

MaleDAPbyCluster <- all[which(all$p_val_adj < 0.05 &
                                all$avg_log2FC > 0.25), ]
FemaleDAPbyCluster <- all[which(all$p_val_adj < 0.05 &
                                  all$avg_log2FC < -0.25), ]

colnames(MaleDAPbyCluster)[1] <- "peak"
MaleDAPbyCluster$cluster <- MaleDAPbyCluster$grp
MaleDAPbyCluster$cluster <- gsub("All_decont_sexed_E11DAP", "", MaleDAPbyCluster$cluster)
MaleDAPbyCluster$cluster <- gsub("All_decont_sexed_E12DAP", "", MaleDAPbyCluster$cluster)
MaleDAPbyCluster$cluster <- gsub("All_decont_sexed_E13DAP", "", MaleDAPbyCluster$cluster)
MaleDAPbyCluster$cluster <- gsub("5_2", "5", MaleDAPbyCluster$cluster)
MaleDAPbyCluster$eday <- MaleDAPbyCluster$grp
MaleDAPbyCluster$eday <- substring(MaleDAPbyCluster$eday, 18, 20) #keep only E11, E12,
E13
MaleDAPbyCluster <- merge(MaleDAPbyCluster, linkspeak_whole, by = c("peak"))

colnames(FemaleDAPbyCluster)[1] <- "peak"
FemaleDAPbyCluster$cluster <- FemaleDAPbyCluster$grp
FemaleDAPbyCluster$cluster <- gsub("All_decont_sexed_E11DAP", "",
FemaleDAPbyCluster$cluster)
FemaleDAPbyCluster$cluster <- gsub("All_decont_sexed_E12DAP", "",
FemaleDAPbyCluster$cluster)
FemaleDAPbyCluster$cluster <- gsub("All_decont_sexed_E13DAP", "",
FemaleDAPbyCluster$cluster)
FemaleDAPbyCluster$cluster <- gsub("5_2", "2", FemaleDAPbyCluster$cluster)
FemaleDAPbyCluster$eday <- FemaleDAPbyCluster$grp
FemaleDAPbyCluster$eday <- substring(FemaleDAPbyCluster$eday, 18, 20) #keep only E11,
E12, E13
FemaleDAPbyCluster <- merge(FemaleDAPbyCluster,
                            linkspeak_whole,
                            by = c("peak"),
                            all.x = TRUE)

write.csv(MaleDAPbyCluster, "MaleDAPbyCluster.csv")
write.csv(FemaleDAPbyCluster, "FemaleDAPbyCluster.csv")
```

ChIP-seeker analysis

```r
sertoli_11DAP <- MaleDAPbyCluster %>%
  filter(cluster == "5" & eday == "E11") %>%
  select(peak)

preG_11DAP <- FemaleDAPbyCluster %>%
  filter(cluster == "2" & eday == "E11") %>%
  select(peak)
```

```r
txdb <- TxDb.Mmusculus.UCSC.mm10.knownGene
peakAnno <- annotatePeak(sertoli_11DAP,
                         tssRegion = c(-3000, 3000),
                         TxDb = txdb)
peakAnno <- annotatePeak(preG_11DAP,
                         tssRegion = c(-3000, 3000),
                         TxDb = txdb)
plotAnnoPie(peakAnno)
```

Peak counts in precursor cell types among E11.5 Sertoli and pre-G DAP regions

```r
#Sertoli
sertoli_11DAP <- StringToGRanges(regions = sertoli_11DAP$peak)
count <- CountsInRegion(object = All_decont_sexed,
                        assay = 'peaksbyc',
                        regions = sertoli_11DAP)
count <- as.data.frame(count)
count$rowname <- rownames(count)
merged_sertoli11 <- merge(count, cluster, by = "rowname", all = TRUE)
filtered_sertoli <- merged_sertoli11[merged_sertoli11$`All_decont_sexed$cluster.SexEday`
%in% c(
  "0_Female11.5", #Epi
  "2_Female11.5", #pre-G
  "4_Female11.5", #PSC
  "0_Male11.5", #Epi
  "5_Male11.5", #Sertoli
  "4_Male11.5" #PSC
), ]

ggplot(
  filtered_sertoli_12,
  aes(
    x = filtered_sertoli_12$`All_decont_sexed$cluster.SexEday`,
    y = count
  )
) +
  geom_violin() +
  stat_summary(
    fun.data = "mean_cl_boot",
    geom = "crossbar",
    colour = "red",
    width = 0.1
  ) +
  theme_minimal()

#pre-G
preG_11DAP <- StringToGRanges(regions = ppreG_11DAP$peak)
count <- CountsInRegion(object = All_decont_sexed,
                        assay = 'peaksbyc',
                        regions = preG_11DAP)
count <- as.data.frame(count)
count$rowname <- rownames(count)
cluster <- as.data.frame(All_decont_sexed$cluster.SexEday)
cluster$rowname <- rownames(cluster)
```

```
merged_preG11 <- merge(count, cluster, by = "rowname", all = TRUE)
filtered_preG <- merged_preG11[merged_preG11$`All_decont_sexed$cluster.SexEday` %in% c(
  "0_Female11.5",
  "2_Female11.5",
  "4_Female11.5",
  "0_Male11.5",
  "5_Male11.5",
  "4_Male11.5"
), ]

ggplot(filtered_preG,
       aes(
         x = filtered_preG$`All_decont_sexed$cluster.SexEday`,
         y = count
       )) +
  geom_violin() +
  stat_summary(
    fun.data = "mean_cl_boot",
    geom = "crossbar",
    colour = "red",
    width = 0.1
  ) +
  theme_minimal()
```

Overlapping Sertoli and pre-G DAPs with published histone marks, using Garciá-Moreno 2019 Dev Biol: ATAC-seq and ChIP-seq (H3K27ac) on E10.5 Sf1 and E13.5 Sertoli and granulosa cells GSE118755 https://www.ncbi.nlm.nih.gov/geo/query/acc.cgi?acc=GSM3346482, and Garciá-Moreno 2019 Plos Genet: ChIP-seq for H3K27me3 and H3K4me3 on E10.5 Sf1 and E13.5 Sertoli and Granulosa cells GSE130749 https://www.ncbi.nlm.nih.gov/geo/query/acc.cgi?acc=GSE130749. These datasets were aligned using mm9, liftover was performed at https://genome.ucsc.edu/cgi-bin/hgLiftOver.

```
#e11.5 sertoli DAP
sertoli_11DAP <- MaleDAPbyCluster %>%
  filter(cluster == "5" & eday == "E11") %>%
  select(peak)
sertoli_11DAP <- unique(sertoli_11DAP)
sertoli_11DAP <- StringToGRanges(regions = sertoli_11DAP$peak)

#e11.5 pre-G DAP
preG_11DAP <- FemaleDAPbyCluster %>%
  filter(cluster == "2" & eday == "E11") %>%
  select(peak)
preG_11DAP <- unique(preG_11DAP)
preG_11DAP <- StringToGRanges(regions = preG_11DAP$peak)

#ac marks
colnames(H3K27ac_preG_mm10) <- c("seqnames", "start", "end")
colnames(H3K27ac_SF1_F_mm10) <- c("seqnames", "start", "end")
colnames(H3K27ac_SF1_M_mm10) <- c("seqnames", "start", "end")
colnames(H3K27ac_Sox9_mm10) <- c("seqnames", "start", "end")

H3K27ac_preG_mm10 <- GRanges(H3K27ac_preG_mm10) #61605
H3K27ac_SF1_F_mm10 <- GRanges(H3K27ac_SF1_F_mm10) #104245
H3K27ac_SF1_M_mm10 <- GRanges(H3K27ac_SF1_M_mm10) #94386
```

```r
H3K27ac_Sox9_mm10 <- GRanges(H3K27ac_Sox9_mm10) #68825

overlap <- as.data.frame(findOverlaps(sertoli_11DAP, H3K27ac_preG_mm10)) # 169 unique
same
overlap <- as.data.frame(findOverlaps(sertoli_11DAP, H3K27ac_SF1_F_mm10)) # 130 unique
127
overlap <- as.data.frame(findOverlaps(sertoli_11DAP, H3K27ac_SF1_M_mm10)) # 118 unique
same
overlap <- as.data.frame(findOverlaps(sertoli_11DAP, H3K27ac_Sox9_mm10)) # 527 unique
same
length(unique(overlap$queryHits))

overlap <- as.data.frame(findOverlaps(preG_11DAP, H3K27ac_preG_mm10)) # 168 unique 166
overlap <- as.data.frame(findOverlaps(preG_11DAP, H3K27ac_SF1_F_mm10)) # 85 unique 82
overlap <- as.data.frame(findOverlaps(preG_11DAP, H3K27ac_SF1_M_mm10)) # 73 unique 71
overlap <- as.data.frame(findOverlaps(preG_11DAP, H3K27ac_Sox9_mm10)) # 63 unique same
length(unique(overlap$queryHits))

#k4 active promoter peaks
colnames(K4_preG_mm10)[1:3] <- c("seqnames", "start", "end")
colnames(K4_SF1_F_mm10)[1:3] <- c("seqnames", "start", "end")
colnames(K4_SF1_M_mm10)[1:3] <- c("seqnames", "start", "end")
colnames(K4_Sox9_mm10)[1:3] <- c("seqnames", "start", "end")

K4_preG_mm10 <- GRanges(K4_preG_mm10) #44316
K4_SF1_F_mm10 <- GRanges(K4_SF1_F_mm10) #41751
K4_SF1_M_mm10 <- GRanges(K4_SF1_M_mm10) #37811
K4_Sox9_mm10 <- GRanges(K4_Sox9_mm10) #39019

overlap <- as.data.frame(findOverlaps(sertoli_11DAP, K4_preG_mm10)) # 99 unique same
overlap <- as.data.frame(findOverlaps(sertoli_11DAP, K4_SF1_F_mm10)) # 92 unique same
overlap <- as.data.frame(findOverlaps(sertoli_11DAP, K4_SF1_M_mm10)) # 55 unique same
overlap <- as.data.frame(findOverlaps(sertoli_11DAP, K4_Sox9_mm10)) # 380 unique same
length(unique(overlap$queryHits))

overlap <- as.data.frame(findOverlaps(preG_11DAP, K4_preG_mm10)) # 154 unique 153
overlap <- as.data.frame(findOverlaps(preG_11DAP, K4_SF1_F_mm10)) # 88 unique 87
overlap <- as.data.frame(findOverlaps(preG_11DAP, K4_SF1_M_mm10)) # 42 unique 41
overlap <- as.data.frame(findOverlaps(preG_11DAP, K4_Sox9_mm10)) # 46 unique same
length(unique(overlap$queryHits))

#k27 repressive mark
colnames(K27_preG_mm10)[1:3] <- c("seqnames", "start", "end")
colnames(K27_SF1_F_mm10)[1:3] <- c("seqnames", "start", "end")
colnames(K27_SF1_M_mm10)[1:3] <- c("seqnames", "start", "end")
colnames(K27_Sox9_mm10)[1:3] <- c("seqnames", "start", "end")

K27_preG_mm10 <- GRanges(K27_preG_mm10) #12586
K27_SF1_F_mm10 <- GRanges(K27_SF1_F_mm10) #73950
K27_SF1_M_mm10 <- GRanges(K27_SF1_M_mm10) #36432
K27_Sox9_mm10 <- GRanges(K27_Sox9_mm10) #74100

overlap <- as.data.frame(findOverlaps(sertoli_11DAP, K27_preG_mm10)) # 46 unique same
overlap <- as.data.frame(findOverlaps(sertoli_11DAP, K27_SF1_F_mm10)) # 70 unique 31
```

```
overlap <- as.data.frame(findOverlaps(sertoli_11DAP, K27_SF1_M_mm10)) # 57 unique 25
overlap <- as.data.frame(findOverlaps(sertoli_11DAP, K27_Sox9_mm10)) # 18 unique 11
length(unique(overlap$queryHits))

overlap <- as.data.frame(findOverlaps(preG_11DAP, K27_preG_mm10)) # 21 unique same
overlap <- as.data.frame(findOverlaps(preG_11DAP, K27_SF1_F_mm10)) # 48 unique 15
overlap <- as.data.frame(findOverlaps(preG_11DAP, K27_SF1_M_mm10)) # 38 unique 15
overlap <- as.data.frame(findOverlaps(preG_11DAP, K27_Sox9_mm10)) # 95 unique 31
length(unique(overlap$queryHits))

#plotting Sertoli DAP overlap
sertoli_overlap <- data.frame(
  AC_mPSC = c(118, 537),
  AC_Sertoli = c(527, 537),
  AC_fPSC = c(127, 537),
  AC_preG = c(169, 537),
  K4_mPSC = c(55, 537),
  K4_Sertoli = c(380, 537),
  K4_fPSC = c(92, 537),
  K4_preG = c(99, 537),
  K27_mPSC = c(25, 537),
  K27_Sertoli = c(11, 537),
  K27_fPSC = c(31, 537),
  K27_preG = c(46, 537)
)
new_row <- sertoli_overlap[1, ] / sertoli_overlap[2, ] * 100
sertoli_overlap <- rbind(sertoli_overlap, new_row)
rownames(sertoli_overlap) <- c("overlap", "total", "percentage")

sertoli_overlap$class <- rownames(sertoli_overlap)
sertoli_overlap_long <- melt(sertoli_overlap)
sertoli_overlap_long <- subset(sertoli_overlap_long, class == "percentage")

ggplot(sertoli_overlap_long, aes(y = value, x = variable)) +
  geom_bar(stat = "identity") +
  theme(
    panel.grid.major = element_blank(),
    panel.grid.minor = element_blank(),
    panel.background = element_blank(),
    axis.line = element_line(colour = "black")
  ) +
  ylim(0, 100)

#plotting pre-G DAP overlap
preG_overlap <- data.frame(
  AC_mPSC = c(71, 195),
  AC_Sertoli = c(63, 195),
  AC_fPSC = c(82, 195),
  AC_preG = c(166, 195),
  K4_mPSC = c(41, 195),
  K4_Sertoli = c(46, 195),
  K4_fPSC = c(87, 195),
  K4_preG = c(153, 195),
  K27_mPSC = c(15, 195),
```

```
  K27_Sertoli = c(31, 195),
  K27_fPSC = c(15, 195),
  K27_preG = c(21, 195)
)
new_row <- preG_overlap[1, ] / preG_overlap[2, ] * 100
preG_overlap <- rbind(preG_overlap, new_row)
rownames(preG_overlap) <- c("overlap", "total", "percentage")

preG_overlap$class <- rownames(preG_overlap)
preG_overlap_long <- melt(preG_overlap)
preG_overlap_long <- subset(preG_overlap_long, class == "percentage")

ggplot(preG_overlap_long, aes(y = value, x = variable)) +
  geom_bar(stat = "identity") +
  theme(
    panel.grid.major = element_blank(),
    panel.grid.minor = element_blank(),
    panel.background = element_blank(),
    axis.line = element_line(colour = "black")
  ) +
  ylim(0, 100)
```

Figure 3- DEG linkage annotation

Cluster DEG b/t sexes

```
#DEG comparison of all celltypes b/t sexes
Idents(All_decont_sexed) <- "cluster.SexEday"
output <- "All_decont_sexed_E11DEG" #repeat for E12 and E13
for (i in 0:13) {
  try({
    ident1 <- paste0(i, "_Male11.5") #repeat for E12 and E13
    ident2 <- paste0(i, "_Female11.5") #repeat for E12 and E13
    condition.diffgenes <- FindMarkers(All_decont_sexed, ident.1 = ident1, ident.2 =
ident2)
    write.csv(condition.diffgenes, file = paste0(output, i, ".csv"))
  })
}
#DEG comparison b/t sertoli and preG directly
E11 <- FindMarkers(All_decont_sexed, ident.1 = "5_Male11.5", ident.2 = "2_Female11.5")
write.csv(E11, "All_decont_sexed_E11DEG5_2.csv")
E12 <- FindMarkers(All_decont_sexed, ident.1 = "5_Male12.5", ident.2 = "2_Female12.5")
write.csv(E12, "All_decont_sexed_E12DEG5_2.csv")
E13 <- FindMarkers(All_decont_sexed, ident.1 = "5_Male13.5", ident.2 = "2_Female13.5")
write.csv(E13, "All_decont_sexed_E13DEG5_2.csv")

fnames = str_remove(list.files(pattern = ".csv"), ".csv")
all <- bind_rows(lapply(fnames, function(fname) {
  read.csv(paste0(fname, ".csv")) %>%
    mutate(grp = fname)
}))

MaleDEGbyCluster <- all[which(all$p_val_adj < 0.05 &
```

```
                                all$avg_log2FC > 0.25), ]
write.csv(MaleDEGbyCluster, "MaleDEGbyCluster.csv")
FemaleDEGbyCluster <- all[which(all$p_val_adj < 0.05 &
                                  all$avg_log2FC < -0.25), ]
write.csv(FemaleDEGbyCluster, "FemaleDEGbyCluster.csv")
```

Annotate DEGs by overlapping with the DAP list: "linked" -> DEG with linked DAP; "non-linked:open" -> DEG with linked non-DAP; "non-linked:close" -> DEG with no linked-peak

```
#male
colnames(MaleDEGbyCluster)[1] <- "gene"
MaleDEGbyCluster$cluster <- MaleDEGbyCluster$grp
MaleDEGbyCluster$cluster <- gsub("All_decont_sexed_E11DEG", "", MaleDEGbyCluster$cluster)
MaleDEGbyCluster$cluster <- gsub("All_decont_sexed_E12DEG", "", MaleDEGbyCluster$cluster)
MaleDEGbyCluster$cluster <- gsub("All_decont_sexed_E13DEG", "", MaleDEGbyCluster$cluster)
MaleDEGbyCluster$eday <- MaleDEGbyCluster$grp
MaleDEGbyCluster$eday <- substring(MaleDEGbyCluster$eday, 18, 20) #keep only E11, E12,
E13
MaleDEGbyCluster <- MaleDEGbyCluster[MaleDEGbyCluster$cluster != "2" &
                                       MaleDEGbyCluster$cluster != "5", ] #remove 2 and
5, then replace 5_2 to 5, making sure to merge with the linkage file in 5
MaleDEGbyCluster$cluster <- gsub("5_2", "5", MaleDEGbyCluster$cluster)

male_anno <- merge(
  MaleDEGbyCluster,
  MaleDAPbyCluster,
  by = c("gene", "cluster", "eday"),
  all.x = TRUE
)
male_anno$annotation <- with(male_anno, ifelse(is.na(peak), "non-linked", "linked"))
non_linked <- subset(male_anno, annotation == "non-linked")
nl_overlap <- merge(non_linked,
                    linkspeak_whole,
                    by = c("gene"),
                    all.x = TRUE)
nl_overlap$annotation <- with(nl_overlap,
                              ifelse(is.na(seqnames.y), "non-linked:close",
                              "non-linked:open"))
nl_overlap <- nl_overlap[, -10:-24]
linked <- subset(male_anno, annotation == "linked")
maleDEGanno_whole <- bind_rows(linked, nl_overlap)
write.csv(maleDEGanno_whole, "maleDEGanno_whole.csv")

#female
colnames(FemaleDEGbyCluster)[1] <- "gene"
FemaleDEGbyCluster$cluster <- FemaleDEGbyCluster$grp
FemaleDEGbyCluster$cluster <- gsub("All_decont_sexed_E11DEG", "",
FemaleDEGbyCluster$cluster)
FemaleDEGbyCluster$cluster <- gsub("All_decont_sexed_E12DEG", "",
FemaleDEGbyCluster$cluster)
FemaleDEGbyCluster$cluster <- gsub("All_decont_sexed_E13DEG", "",
FemaleDEGbyCluster$cluster)
FemaleDEGbyCluster$eday <- FemaleDEGbyCluster$grp
FemaleDEGbyCluster$eday <- substring(FemaleDEGbyCluster$eday, 18, 20) #keep only E11,
E12, E13
```

```r
FemaleDEGbyCluster <- FemaleDEGbyCluster[FemaleDEGbyCluster$cluster != "2" &
                                           FemaleDEGbyCluster$cluster != "5", ]
FemaleDEGbyCluster$cluster <- gsub("5_2", "2", FemaleDEGbyCluster$cluster)

female_anno <- merge(
  FemaleDEGbyCluster,
  FemaleDAPbyCluster,
  by = c("gene", "cluster", "eday"),
  all.x = TRUE
)
female_anno$annotation <- with(female_anno, ifelse(is.na(peak), "non-linked", "linked"))
non_linked <- subset(female_anno, annotation == "non-linked")
nl_overlap <- merge(non_linked,
                    linkspeak_whole,
                    by = c("gene"),
                    all.x = TRUE)
nl_overlap$annotation <- with(nl_overlap,
                              ifelse(is.na(seqnames.y), "non-linked:close",
                              "non-linked:open"))
nl_overlap <- nl_overlap[, -10:-24]
linked <- subset(female_anno2, annotation == "linked")
femaleDEGanno_whole <- bind_rows(linked, nl_overlap)
write.csv(femaleDEGanno_whole, "femaleDEGanno_whole.csv")

#plotting
annotation <- maleDEGanno_whole %>% #or femaleDEGanno_whole
  group_by(cluster, eday, annotation) %>%
  summarise(count = n_distinct(gene)) #this way looks at distinct genes, and the number
  is the same with MaleDEGbycluster number
annotation <- melt(dcast(annotation, annotation ~ cluster + eday))
annotation[is.na(annotation)] = 0

ggplot(annotation, aes(
  fill = factor(
    annotation,
    levels = c("linked", "non-linked:open", "non-linked:close")
  ),
  y = value,
  x = variable
)) +
  geom_bar(position = "stack", stat = "identity") +
  scale_fill_manual(name = "Linkage",
                    values = c("#edae49", "#00798c", "#8d96a3")) +
  theme(
    panel.grid.major = element_blank(),
    panel.grid.minor = element_blank(),
    panel.background = element_blank(),
    axis.line = element_line(colour = "black")
  ) +
  ylim(0, 1050) #or 770 for female
```

Sankey plots

```r
#sertoli DEG
sertoli <- maleDEGanno_whole %>%
  filter(cluster == "5") %>%
  group_by(gene, eday) %>%
  select(gene, eday, annotation)
sertoli <- sertoli[!duplicated(sertoli), ] #after removing duplicates = 2675
sertoli$annotation <- factor(sertoli$annotation,
                             levels = c("linked", "non-linked:open", "non-linked:close"))
ggplot(
  sertoli,
  aes(
    x = eday,
    stratum = annotation,
    alluvium = gene,
    fill = annotation,
    label = annotation
  )
) +
  scale_fill_manual(name = "Annotation",
                    values = c("#edae49", "#00798c", "#8d96a3")) +
  geom_flow(stat = "alluvium", lode.guidance = "frontback") +
  geom_stratum(size = 0) +
  theme(
    legend.position = "bottom",
    panel.grid.major = element_blank(),
    panel.grid.minor = element_blank(),
    panel.background = element_blank(),
    axis.line = element_line(colour = "black")
  ) +
  ggtitle("sertoli DEG annotation") +
  ylim(0, 1050)

#pre-G DEG
preG <- femaleDEGanno_whole %>%
  filter(cluster == "2") %>%
  group_by(gene, eday) %>%
  select(gene, eday, annotation)
preG <- preG[!duplicated(preG), ] #whole after removing duplicates= 1782
preG$annotation <- factor(preG$annotation,
                          levels = c("linked", "non-linked:open", "non-linked:close"))
ggplot(
  preG,
  aes(
    x = eday,
    stratum = annotation,
    alluvium = gene,
    fill = annotation,
    label = annotation
  )
) +
  scale_fill_manual(name = "Annotation",
                    values = c("#edae49", "#00798c", "#8d96a3")) +
  geom_flow(stat = "alluvium", lode.guidance = "frontback") +
  geom_stratum(size = 0) +
```

```
  theme(
    legend.position = "bottom",
    panel.grid.major = element_blank(),
    panel.grid.minor = element_blank(),
    panel.background = element_blank(),
    axis.line = element_line(colour = "black")
  ) +
  ggtitle("preG DEG annotation") +
  ylim(0, 770)
```

Figure 4- Motif enrichment within Sertoli DAPs, motif scan, and PSC vs Sertoli DEG/ DAP analysis

Download motif PFMs directly from JASPAR2022 site using "vertebrates, CORE, and non-redundant" settings

```
JASPAR2022_ID <- JASPAR2022_CORE_non.redundant_pfms_jaspar %>% mutate_all(na_if, "")
JASPAR2022_ID <- na.omit(JASPAR2022_ID)
JASPAR2022_ID <- gsub(">", "", JASPAR2022_ID$V1)
pfm_2022 <- getMatrixByID(x = JASPAR2022, ID = JASPAR2022_ID) #in total 1956 elements
```

Motif enrichment within Sertoli E11.5 "DEG with linked DAP" regions

```
#adding the jaspar2022 motif set to the subsetted sertoli dataset
Idents(All_decont_sexed) <- "SCT_snn_res.0.3"
Sertoli <- subset(All_decont_sexed, SCT_snn_res.0.3 == "5" &
                    Sex == "Male")
Sertoli <- AddMotifs(
  object = Sertoli,
  assay = "peaksbyc",
  genome = BSgenome.Mmusculus.UCSC.mm10,
  pfm = pfm_2022
)
meta.feature <- GetAssayData(Sertoli, assay = "peaksbyc", slot = "meta.features")

#motif enrichment within Sertoli "E11.5 DEG with linked DAP"
sertoli11_orange <- maleDEGanno_whole %>%
  filter(cluster == "5" & eday == "E11" & annotation == "linked") %>%
  group_by(gene) %>%
  select(gene, eday, annotation, peak)

open.peaks <- AccessiblePeaks(Sertoli, assay = "peaksbyc", cells = Sertoli$Eday ==
"E11.5")
peaks.matched <- MatchRegionStats(meta.feature = meta.feature[open.peaks, ],
query.feature = meta.feature[sertoli11_orange$peak, ])
enriched.motifs <- FindMotifs(
  object = Sertoli,
  assay = "peaksbyc",
  features = sertoli11_orange$peak,
  background = peaks.matched
)
filtered_enriched.motifs <- enriched.motifs %>%
```

```r
  filter(p.adjust < 0.05 &
           fold.enrichment > 1.25) #1956 before and 33 after filtering
filtered_enriched.motifs <- filtered_enriched.motifs %>%
  mutate(motif.name = stringr::str_to_title(motif.name)) %>% #reorganize the case of the
  motifs
  separate(
    col = motif.name,
    into = c("first", "second", "third"),
    sep = "::",
    remove = FALSE
  ) %>% #separate compound motifs and overlap with DEG lists to check for expression
  mutate(first = gsub("\\s*\\([^\\)]+\\)", "", first)) #remove () and everything inside

#overlap with Sertoli DEG
sertoli_DEG <- maleDEGanno_whole %>%
  filter(cluster == "5" & eday == "E11")
sertoli_motif <- merge(filtered_enriched.motifs,
                       sertoli_DEG,
                       by.x = "first",
                       by.y = "gene") #12 for jsp22, 5 distinct
MotifPlot(
  Sertoli,
  assay = "peaksbyc",
  motifs = sertoli_motif$motif
)

#GEX dotplot
DefaultAssay(All_decont_sexed) <- 'SCT'
Idents(All_decont_sexed) <- "cluster.SexEday"
idents <- c(
  "0_Male11.5",
  "4_Male11.5",
  "5_Male11.5",
  "0_Female11.5",
  "4_Female11.5",
  "2_Female11.5"
)
DotPlot(
  All_decont_sexed,
  assay = 'SCT',
  features = c("Sox10", "Sox13", "Sox4", "Sox6", "Sox9"),
  idents = factor(
    idents,
    levels = c(
      "0_Male11.5",
      "0_Female11.5",
      "4_Male11.5",
      "4_Female11.5",
      "5_Male11.5",
      "2_Female11.5"
    )
  ),
  cols = c("lightgrey", "#6A040F")
) +
```

```
  coord_flip()
```

Motif enrichment within Sertoli "newly formed DEG with linked DAP" regions from E11.5 to E12.5: What are the new linked DEG/ DAPs at E12.5 that are not present in E11.5?

```
#"newly formed" includes "new orange at E12.5" and "transitioning from teal to orange"
#first get the "new orange at E12.5" regions
sertoli11 <- maleDEGanno_whole %>%
  filter(cluster == "5" & eday == "E11") %>%
  group_by(gene) %>%
  select(gene, eday, annotation, peak, zscore, peak.y, zscore.y)
sertoli12 <- maleDEGanno_whole %>%
  filter(cluster == "5" & eday == "E12") %>%
  group_by(gene) %>%
  select(gene, eday, annotation, peak, zscore, peak.y, zscore.y)
merge <- merge(
  sertoli11,
  sertoli12,
  by.x = c("gene"),
  by.y = c("gene"),
  all = TRUE
)
new12 <- merge %>%
  filter(is.na(eday.x) & annotation.y == "linked")

#extract transitioning (teal to orange) regions
transitioning <- merge %>%
  filter(eday.x == "E11" & annotation.x == "non-linked:open") %>%
  filter(eday.y == "E12" & annotation.y == "linked") %>%
  filter(peak.y == peak.y.x)

#combining both new orange and teal to orange
newly_linked <- rbind(new12, transitioning) #756 obs, use peak.y for all peaks #667
unique

#motif enrichment
open.peaks <- AccessiblePeaks(Sertoli,
                              assay = "peaksbyc",
                              cells = Sertoli$Eday == "E11.5" |
                                Sertoli$Eday == "E12.5")
peaks.matched <- MatchRegionStats(meta.feature = meta.feature[open.peaks, ],
query.feature = meta.feature[newlylinked$peak.y, ])
enriched.motifs <- FindMotifs(
  object = Sertoli,
  assay = "peaksbyc",
  features = newlylinked$peak.y,
  background = peaks.matched
)
filtered_enriched.motifs <- enriched.motifs %>%
  filter(p.adjust < 0.05 &
           fold.enrichment > 1.25) #1956 before and 577 after filtering

#double checked if there are any '/' -> Klf3/8/12 is the only one, I only have Klf1-5 in
my data so I think it's ok not to separate by /
```

```
filtered_enriched.motifs <- filtered_enriched.motifs %>%
  mutate(motif.name = stringr::str_to_title(motif.name)) %>% #reorganize the case of the
  motifs
  separate(
    col = motif.name,
    into = c("first", "second", "third"),
    sep = "::",
    remove = FALSE
  ) %>% #separate compound motifs and see if everything is expressed
  mutate(first = gsub("\\s*\\([^\\)]+\\)", "", first)) #remove () and everything inside
test <- rbind(
  filtered_enriched.motifs,
  #using rbind to duplicate motifs with more than one genes
  filtered_enriched.motifs %>%
    filter(!is.na(second)) %>%
    mutate(first = second),
  filtered_enriched.motifs %>%
    filter(!is.na(third)) %>%
    mutate(first = third)
)

#overlapping with DEG
sertoli_DEG <- maleDEGanno_whole %>%
  filter(cluster == "5") %>%
  filter(eday == "E11" | eday == "E12")

sertoli_motif <- merge(test, sertoli_DEG, by.x = "first", by.y = "gene") #23 unique
motifs
MotifPlot(
  object = Sertoli,
  assay = "peaksbyc",
  motifs = unique(sertoli_motif$motif)
)

#GEX dotplot
DotPlot(
  All_decont_sexed,
  assay = 'SCT',
  features = c(
    "Sox10",
    "Sox13",
    "Sox4",
    "Sox6",
    "Sox8",
    "Sox9",
    "Nr5a1",
    "Nr6a1",
    "Klf13",
    "Thra",
    "Glis3",
    "Foxn3",
    "Jun",
    "Gata4",
    "Irf2",
```

```
    "Mef2a",
    "Mef2c",
    "Dmrt1",
    "Tead1",
    "Tgif1"
  ),
  idents = c(
    "0_Male11.5",
    "0_Male12.5",
    "4_Male11.5",
    "4_Male12.5",
    "5_Male11.5",
    "5_Male12.5",
    "0_Female11.5",
    "0_Female12.5",
    "4_Female11.5",
    "4_Female12.5",
    "2_Female11.5",
    "2_Female12.5"
  ),
  cols = c("lightgrey", "#6A040F")
) +
  coord_flip()
```

Run motif scan using tutorial:https://motifscan.readthedocs.io/en/latest/index.html

```
#writing peak files as motif scan input
granges.df <- as.data.frame(All_decont_sexed@assays$peaksbyc@ranges)
write.table(
  granges.df,
  file = "~/.motifscan/peaks.bed",
  row.names = FALSE,
  col.names = FALSE,
  quote = FALSE,
  sep = "\t"
)
```

Sort the peak file before running motif scan

```
sort -k 1,1 -k2,2n peaks.bed > peaks.sorted.bed

#using default motif scan installation location
motifscan config --set-default-genome $HOME/.motifscan/genomes/
motifscan config --set-default-motif $HOME/.motifscan/motifs/

#installing mm10 assembly
motifscan genome --install -n mm10 -r mm10

#install a motif set with my downloaded PFMs file
motifscan motif --install -n JASPAR -i ~/JASPAR2022_CORE_non-redundant_pfms_jaspar.txt
-g mm10

#scanning motifs
```

```
motifscan scan --site -i peaks.sorted.bed -g mm10 -m JASPAR -o motif #output 1956 bed
files
```

Extract Sertoli motifs and overlap with Sertoli DAP regions

```
#look for the bed files that are enriched sertoli motifs
list <- list.files("/yourpath/motif_sites/")
search <- unique(sertoli_motif$motif)
search <- gsub("\\.", "_", search)
matches_list <- as.list(lapply(
  search,
  grep,
  x = list,
  value = TRUE,
  ignore.case = TRUE
))

#extract sertoli peaks
sertoliDAP <- MaleDAPbyCluster %>%
  filter(cluster == "5") %>%
  filter(eday == "E11" | eday == "E12")
sertoliDAP_ranges <- StringToGRanges(sertoliDAP$peak, sep = c("-", "-"))

#overlap sertoli motif bed files with sertoli peaks -> where are the enriched motifs
located within sertoli DAPs?
for (file in matches_list) {
  granges = readBed(file, track.line = FALSE, remove.unusual = FALSE)
  result <- as.data.frame(findOverlaps(granges, sertoliDAP_ranges))
  hit <- sertoliDAP[result$subjectHits, ]
  csv <- read.delim(file, header = FALSE)
  motif <- csv[result$queryHits, ]
  write.csv(hit, file = paste0("/yourpath/overlaps/", gsub('.bed', '_linkedDAP.csv',
  file)))
  write.csv(motif, file = paste0("/yourpath/overlaps/", gsub('.bed', '_motif.csv',
  file)))
}
```

Identify linked motif targets

```
#in the folder where all output _linkedDAP files are saved
#identify positively linked gene (zscore>0)
matches_list = list.files(pattern = "linkedDAP.csv")
output <- data_frame()
for (file in matches_list) {
  csv <- read_csv(file)
  gene <- as.data.frame(t(as.data.frame(unique(csv[csv$zscore > 0, ]$gene)))) #t a
dataframe with one column makes it chr string
  output <- bind_rows(output, gene)
}
output <- as.data.frame(t(output))
colnames(output) <- matches_list
write.csv(output, "SertoliMotif_targetGene.csv")
```

```r
#overlapping positively linked genes with sertoli DEG (this is done for both E11.5 and
E12.5 separately)
sertoliDEG12 <- maleDEGanno_whole %>%
  filter(cluster == "5" & eday == "E12")

filter_target <- data.frame()
names <- names(SertoliMotif_targetGene)
for (col_name in names(SertoliMotif_targetGene)) {
  filter_row <- as.data.frame(t(
    filter(
      SertoliMotif_targetGene[col_name],
      SertoliMotif_targetGene[[col_name]] %in% sertoliDEG12$gene
    )
  ))
  filter_target <- bind_rows(filter_target, filter_row)
}
filter_target <- as.data.frame(t(filter_target))
write.csv(filter_target, "SertoliMotif_targetGene_DEG.csv")

#repeat with negatively linked gene
output2 <- data_frame()
for (file in matches_list) {
  csv <- read_csv(file)
  gene <- as.data.frame(t(as.data.frame(unique(csv[csv$zscore < 0, ]$gene)))) #t a
dataframe with one column makes it chr string
  gene$file_name <- file
  output2 <- bind_rows(output2, gene)
}
output2 <- as.data.frame(t(output2))
colnames(output2) <- matches_list
write.csv(output2, "SertoliMotif_NEGATIVE_targetGene.csv")

#plotting heatmaps of linked GEX
#heatmap of linked gene
sertoli_pos <- unique(unlist(SertoliMotif_targetGene_DEG))
sertoli_neg <- unique(unlist(SertoliMotif_NEGATIVE_targetGene))
sertoli_all <- c(sertoli_pos, sertoli_neg)
sertoli_all <- sertoli_all[!is.na(sertoli_all)]

DefaultAssay(All_decont_sexed) <- 'SCT'
Idents(All_decont_sexed) <- "cluster.SexEday"
cells <- WhichCells(
  All_decont_sexed,
  ident = c("5_Male11.5", "5_Male12.5", "2_Female11.5", "2_Female12.5")
)

DoHeatmap(
  All_decont_sexed,
  features = sertoli_all,
  cells = cells,
  assay = "SCT",
  slot = "data",
  group.by = "ident",
  label = TRUE,
```

```
) + scale_fill_gradientn(colors = c("blue", "white", "red"))
```

Computing motif co-occurrence

```
sertoli_freq <- as.data.frame(table(unlist(SertoliMotif_targetGene_DEG)))
name <- sertoli_freq$Var1
output <- data_frame()
for (gene in name) {
  motif <- as.data.frame(t(as.data.frame(
    colnames(SertoliMotif_targetGene_DEG[, grepl(paste0("\\b", gene, "\\b"),
    SertoliMotif_targetGene_DEG)])
  )))
  output <- bind_rows(output, motif)
}
output <- cbind(sertoli_freq, output)
write.csv(output, "Sertoli_targetDEG_Freq.csv")

foo <- Sertoli_targetDEG_Freq[, -2]
colnames(foo)[1] <- "Gene"
foo <- melt(setDT(foo), id.vars = "Gene", measure = patterns("^V"))[nchar(value) > 0 &
                                                                   complete.cases
                                                                   (value)]

mymat <- crossprod(table(foo[, c(1, 3)])) #create a matrix with crosspod
diag(mymat) <- 0 #change diagonal values to 0

#plot co-occurance as a heatmap
colnames(mymat) <- gsub("_sites_linkedDAP.csv", "", colnames(mymat))
rownames(mymat) <- gsub("_sites_linkedDAP.csv", "", rownames(mymat))
colnames(mymat) <- rownames(mymat)
palf <- colorRampPalette(c("lightgrey", "#6A040F"))
heatmap.2(
  mymat,
  col = palf(10),
  trace = "none",
  Rowv = FALSE,
  Colv = FALSE,
  margins = c(20, 20)
)
```

chromVAR motif activity

```
#at this point, motif data is added to the All_decont_sexed dataset and chromVAR is run
on this object as well
All_decont_sexed <- AddMotifs(
  object = All_decont_sexed,
  assay = "peaksbyc",
  genome = BSgenome.Mmusculus.UCSC.mm10,
  pfm = pfm_2022
)
All_decont_sexed <- RunChromVAR(object = All_decont_sexed,
                                genome = BSgenome.Mmusculus.UCSC.mm10,
                                assay = 'peaksbyc')
```

```r
saveRDS(All_decont_sexed, file = "All_decont_sexed.rds")

#plotting GEX
DefaultAssay(All_decont_sexed) <- 'chromvar'
Idents(All_decont_sexed) <- "cluster.SexEday"
DotPlot(
  All_decont_sexed,
  assay = 'chromvar',
  features = unique(sertoli_motif$motif),
  idents = c(
    "0_Male11.5",
    "0_Male12.5",
    "4_Male11.5",
    "4_Male12.5",
    "5_Male11.5",
    "5_Male12.5",
    "0_Female11.5",
    "0_Female12.5",
    "4_Female11.5",
    "4_Female12.5",
    "2_Female11.5",
    "2_Female12.5"
  )
  cols = c("lightgrey", "#6A040F")
)
+ coord_flip() + scale_x_discrete(limits = rev)

#plotting UMAP and chromVAR featureplots
#subset male and female supporting cells
Idents(All_decont_sexed) <- "cluster.SexEday"
supporting <- subset(
  All_decont_sexed,
  cluster.SexEday == "0_Male11.5" |
    cluster.SexEday == "0_Male12.5" |
    cluster.SexEday == "0_Male13.5" |
    cluster.SexEday == "4_Male11.5" |
    cluster.SexEday == "4_Male12.5" |
    cluster.SexEday == "4_Male13.5" |
    cluster.SexEday == "5_Male11.5" |
    cluster.SexEday == "5_Male12.5" |
    cluster.SexEday == "5_Male13.5"
)

DefaultAssay(supporting) <- "RNA"
supporting <- SCTransform(supporting, verbose = TRUE)
supporting <- RunPCA(supporting)
pct <- supporting@reductions$pca@stdev / sum(supporting@reductions$pca@stdev) *100 #
Determine the pc to use
co2 <- sort(which((pct[1:length(pct) - 1] - pct[2:length(pct)]) > 0.1), decreasing =
TRUE)[1] + 1

DefaultAssay(supporting) <- "peaksbyc"
supporting <- FindTopFeatures(supporting, min.cutoff = 5)
supporting <- RunTFIDF(supporting)
```

```r
supporting <- RunSVD(supporting)
supporting <- FindMultiModalNeighbors(
  supporting,
  reduction.list = list("pca", "lsi"),
  dims.list = list(1:co2, 2:30)
)
supporting <- RunUMAP(
  supporting,
  nn.name = "weighted.nn",
  reduction.name = "wnn.umap",
  reduction.key = "wnnUMAP_"
)
DimPlot(
  supporting,
  reduction = "wnn.umap",
  label = FALSE,
  label.size = 4,
  group.by = "Eday",
  cols = c("#92DBDD", "#00BFC4", "#0A5A5C")
)
DimPlot(
  supporting,
  reduction = "wnn.umap",
  label = FALSE,
  label.size = 4,
  group.by = "SCT_snn_res.0.3",
  cols = c('#9E2A2B', '#3A5A40', "#A1D3E9")
)

DefaultAssay(supporting) <- 'chromvar'
FeaturePlot(
  supporting,
  reduction = "wnn.umap",
  keep.scale = "feature",
  features = c("MA0077.1", "MA1603.1", "MA1127.1", "MA0497.1"),
  cols = c("lightgrey", "#6A040F"),
  order = TRUE,
  min.cutoff = "q1",
  max.cutoff = "q99"
)
```

Comparing E11.5 XY PSC to Sertoli cells

```r
#DAPs
DefaultAssay(All_decont_sexed) <- "peaksbyc"
male_dap <- FindMarkers(
  All_decont_sexed,
  ident.1 = "4_Male11.5",
  ident.2 = "5_Male11.5",
  logfc.threshold = .1,
  min.pct = 0.01,
  test.use = 'LR',
  latent.vars = 'nCount_peaksbyc'
)
```

```r
male_dap_psc <- male_dap[which(male_dap$p_val_adj < 0.05 &
                                 male_dap$avg_log2FC > 0.25), ]
male_dap_sertoli <- male_dap[which(male_dap$p_val_adj < 0.05 &
                                     male_dap$avg_log2FC < 0.25), ]
male_dap_psc$cluster <- "4"
male_dap_sertoli$cluster <- "5"

male_dap_psc_linked <- merge(male_dap_psc,
                             linkspeak_whole,
                             by = c("peak"),
                             all.x = TRUE)
male_dap_sertoli_linked <- merge(male_dap_sertoli,
                                 linkspeak_whole,
                                 by = c("peak"),
                                 all.x = TRUE)

male_dap_psc_anno <- merge(male_dap_psc_linked,
                           male_deg_psc,
                           by = c("gene"),
                           all.x = TRUE)
male_dap_sertoli_anno <- merge(
  male_dap_sertoli_linked,
  male_deg_sertoli,
  by = c("gene"),
  all.x = TRUE
)

male_dap_psc_anno$annotation <- with(male_dap_psc_anno, ifelse(is.na(gene), "non-linked",
"linked"))
male_dap_sertoli_anno$annotation <- with(male_dap_sertoli_anno, ifelse(is.na(gene),
"non-linked", "linked"))

male_dap_psc_anno <- male_dap_psc_anno %>%
  mutate(
    annotation = case_when(
      annotation == "linked" & is.na(p_val.y) ~ "linked:noDEG",
      annotation == "linked" &
        !is.na(p_val.y) ~ "linked:DEG",
      annotation == "non-linked" ~ "nolink"
    )
  )

male_dap_sertoli_anno <- male_dap_sertoli_anno %>%
  mutate(
    annotation = case_when(
      annotation == "linked" & is.na(p_val.y) ~ "linked:noDEG",
      annotation == "linked" &
        !is.na(p_val.y) ~ "linked:DEG",
      annotation == "non-linked" ~ "nolink"
    )
  )
combined <- bind_rows(male_dap_psc_anno, male_dap_sertoli_anno)

annotation <- combined %>%
```

```r
  group_by(cluster, annotation) %>%
  summarise(count = n_distinct(peak))

annotation <- melt(dcast(annotation, annotation ~ cluster))
annotation[is.na(annotation)] = 0

ggplot(annotation, aes(
  fill = factor(annotation, levels = c("linked:DEG", "linked:noDEG", "nolink")),
  y = value,
  x = variable
)) +
  geom_bar(position = "stack", stat = "identity") +
  scale_fill_manual(name = "Linkage",
                    values = c("#edae49", "#00798c", "#8d96a3")) +
  theme(
    panel.grid.major = element_blank(),
    panel.grid.minor = element_blank(),
    panel.background = element_blank(),
    axis.line = element_line(colour = "black")
  ) +
  ylim(0, 1000) #figure 4I didn't show linkage annotations but only DAP number

#DEG annotation
male_deg <- FindMarkers(All_decont_sexed, ident.1 = "4_Male11.5", ident.2 = "5_Male11.5")
male_deg_psc <- male_deg[which(male_deg$p_val_adj < 0.05 &
                                 male_deg$avg_log2FC > 0.25), ]
male_deg_sertoli <- male_deg[which(male_deg$p_val_adj < 0.05 &
                                     male_deg$avg_log2FC < -0.25), ]
male_deg_psc$cluster <- "4"
male_deg_sertoli$cluster <- "5"

male_deg_psc_anno <- merge(male_deg_psc,
                           linkspeak_whole,
                           by = c("gene"),
                           all.x = TRUE)
male_deg_psc_anno$annotation <- with(male_deg_psc_anno, ifelse(is.na(peak), "non-linked",
"linked"))

male_deg_psc_anno <- merge(male_deg_psc_anno,
                           male_dap_psc_linked,
                           by = c("gene"),
                           all.x = TRUE)
male_deg_psc_anno <- male_deg_psc_anno %>%
  mutate(
    annotation = case_when(
      annotation == "linked" & is.na(p_val.y) ~ "linked:noDAP",
      annotation == "linked" &
        !is.na(p_val.y) ~ "linked:DAP",
      annotation == "non-linked" ~ "nolink"
    )
  )

male_deg_sertoli_anno <- merge(male_deg_sertoli,
                               linkspeak_whole,
```

```
                                by = c("gene"),
                                all.x = TRUE)
male_deg_sertoli_anno$annotation <- with(male_deg_sertoli_anno, ifelse(is.na(peak),
"non-linked", "linked"))

male_deg_sertoli_anno <- merge(
  male_deg_sertoli_anno,
  male_dap_sertoli_linked,
  by = c("gene"),
  all.x = TRUE
)
male_deg_sertoli_anno <- male_deg_sertoli_anno %>%
  mutate(
    annotation = case_when(
      annotation == "linked" & is.na(p_val.y) ~ "linked:noDAP",
      annotation == "linked" &
        !is.na(p_val.y) ~ "linked:DAP",
      annotation == "non-linked" ~ "nolink"
    )
  )

male_deg_anno <- Male_E11_PSC_DEGanno %>%
  group_by(annotation) %>%
  summarise(count = n_distinct(gene))
male_deg_anno$cluster <- "4"

sertoli_anno <- Male_E11_sertoli_DEGanno %>%
  group_by(annotation) %>%
  summarise(count = n_distinct(gene))
sertoli_anno$cluster <- "5"

plot <- rbind(male_deg_anno, sertoli_anno)

ggplot(plot, aes(
  fill = factor(annotation, levels = c("linked:DAP", "linked:noDAP", "nolink")),
  y = count,
  x = cluster
)) +
  geom_bar(position = "stack", stat = "identity") +
  scale_fill_manual(name = "Linkage",
                    values = c("#edae49", "#00798c", "#8d96a3")) +
  theme(
    panel.grid.major = element_blank(),
    panel.grid.minor = element_blank(),
    panel.background = element_blank(),
    axis.line = element_line(colour = "black")
  ) +
  ylim(0, 650)
```

Figure 5- Motif enrichment within pre-G DAPs and PSC vs pre-G DAP analysis

Motif enrichment within pre-G E11.5 "DEG with linked DAP" regions

```r
#adding the jaspar2022 motif set to the subsetted pre-G dataset
Idents(All_decont_sexed) <- "SCT_snn_res.0.3"
preG <- subset(All_decont_sexed, SCT_snn_res.0.3 == "2" &
                 Sex == "Female")
preG <- AddMotifs(
  object = preG,
  assay = "peaksbyc",
  genome = BSgenome.Mmusculus.UCSC.mm10,
  pfm = pfm_2022
)
meta.feature <- GetAssayData(preG, assay = "peaksbyc", slot = "meta.features")

#motif enrichment within pre-G "E11.5 DEG with linked DAP"
preG11_orange <- femaleDEGanno_whole %>%
  filter(cluster == "2" &
           eday == "E11" & annotation == "linked") %>%
  group_by(gene, eday, annotation, peak)

open.peaks <- AccessiblePeaks(preG, assay = "peaksbyc", cells = preG$Eday == "E11.5")
peaks.matched <- MatchRegionStats(meta.feature = meta.feature[open.peaks, ],
query.feature = meta.feature[preG11_orange$peak, ])
enriched.motifs <- FindMotifs(
  object = preG,
  assay = "peaksbyc",
  features = preG11_orange$peak,
  background = peaks.matched
)

filtered_enriched.motifs <- enriched.motifs %>%
  filter(p.adjust < 0.05 &
           fold.enrichment > 1.25) #1956 before and 127 after filtering

filtered_enriched.motifs <- filtered_enriched.motifs %>%
  mutate(motif.name = stringr::str_to_title(motif.name)) %>% #reorganize the case of the
  motifs
  separate(
    col = motif.name,
    into = c("first", "second", "third"),
    sep = "/",
    remove = FALSE
  ) %>% #separate specifically Lhx3/4
  mutate(first = gsub("\\s*\\([^\\)]+\\)", "", first)) #remove () and everything inside

test <- rbind(
  filtered_enriched.motifs,
  #using rbind to duplicate motifs with more than one gene names
  filtered_enriched.motifs %>%
    filter(!is.na(second)) %>%
    mutate(first = second),
  filtered_enriched.motifs %>%
    filter(!is.na(third)) %>%
    mutate(first = third)
)
test <- test %>%
```

```
  mutate(first = gsub("\\s*\\([^\\)]+\\)", "", first))
test$first[128] <- "Lhx4"

#overlapping with DEG
preG_DEG <- femaleDEGanno_whole %>%
  filter(cluster == "2" & eday == "E11")

preG_motif <- merge(filtered_enriched.motifs,
                    preG_DEG,
                    by.x = "first",
                    by.y = "gene") #5 for jsp22, 2 distinct

#GEX dotplot
DefaultAssay(All_decont_sexed) <- 'SCT'
Idents(All_decont_sexed) <- "cluster.SexEday"
idents <- c(
  "0_Male11.5",
  "4_Male11.5",
  "5_Male11.5",
  "0_Female11.5",
  "4_Female11.5",
  "2_Female11.5"
)
DotPlot(
  All_decont_sexed,
  assay = 'SCT',
  features = c("Lef1", "Msx1"),
  idents = factor(
    idents,
    levels = c(
      "0_Male11.5",
      "0_Female11.5",
      "4_Male11.5",
      "4_Female11.5",
      "5_Male11.5",
      "2_Female11.5"
    )
  ),
  cols = c("lightgrey", "#6A040F")
) +
  coord_flip()
```

Motif enrichment within pre-G “newly formed DEG with linked DAP” regions from E11.5 to E12.5

```
#"newly formed" includes "new orange at E12.5" and "transitioning from teal to orange"
#first get the "new orange at E12.5" regions
preG11 <- femaleDEGanno_whole %>%
  filter(cluster == "2" & eday == "E11") %>%
  group_by(gene) %>%
  select(gene, eday, annotation, peak, zscore, peak.y, zscore.y)
preG12 <- femaleDEGanno_whole %>%
  filter(cluster == "2" & eday == "E12") %>%
  group_by(gene) %>%
  select(gene, eday, annotation, peak, zscore, peak.y, zscore.y)
```

```
merge <- merge(
  preG11,
  preG12,
  by.x = c("gene"),
  by.y = c("gene"),
  all = TRUE
)
new12 <- merge %>%
  filter(is.na(eday.x) & annotation.y == "linked")

#extract transitioning (teal to orange) regions
transitioning <- merge %>%
  filter(eday.x == "E11" & annotation.x == "non-linked:open") %>%
  filter(eday.y == "E12" & annotation.y == "linked") %>%
  filter(peak.y == peak.y.x)

#combining both new orange and teal to orange
newly_linked <- rbind(new12, transitioning) #570 obs, use peak.y for all peaks #530
unique peaks


#motif enrichment
open.peaks <- AccessiblePeaks(preG,
                              assay = "peaksbyc",
                              cells = preG$Eday == "E11.5" |
                                preG$Eday == "E12.5")
peaks.matched <- MatchRegionStats(meta.feature = meta.feature[open.peaks, ],
query.feature = meta.feature[newlylinked$peak.y, ])
enriched.motifs <- FindMotifs(
  object = preG,
  assay = "peaksbyc",
  features = newlylinked$peak.y,
  background = peaks.matched
)
filtered_enriched.motifs <- enriched.motifs %>%
  filter(p.adjust < 0.05 &
           fold.enrichment > 1.25) #1956 before and 1691 after filtering

filtered_enriched.motifs <- filtered_enriched.motifs %>%
  mutate(motif.name = stringr::str_to_title(motif.name)) %>% #reorganize the case of the
  motifs
  separate(
    col = motif.name,
    into = c("first", "second", "third"),
    sep = "::",
    remove = FALSE
  ) %>% #separate individual motifs
  mutate(first = gsub("\\s*\\([^\\)]+\\)", "", first)) #remove () and everything inside

filtered2 <- filtered_enriched.motifs %>%
  mutate(motif.name = stringr::str_to_title(motif.name)) %>% #reorganize the case of the
  motifs
  separate(
    col = motif.name,
```

```r
    into = c("first", "second", "third"),
    sep = "/",
    remove = FALSE
  ) %>% #separate individual motifs
  mutate(second = ifelse(!is.na(second), paste(gsub("[0-9]*$", "", first), second, sep =
  ""), second)) %>%
  mutate(third = ifelse(!is.na(third), paste(gsub("[0-9]*$", "", first), third, sep =
  ""), third)) %>%
  filter(!is.na(second))

test <- rbind(
  filtered_enriched.motifs,
  #using rbind to duplicate motifs with more than one gene names
  filtered_enriched.motifs %>%
    filter(!is.na(second)) %>%
    mutate(first = second),
  filtered_enriched.motifs %>%
    filter(!is.na(third)) %>%
    mutate(first = third),
  filtered2,
  filtered2 %>%
    mutate(first = second),
  filtered2 %>%
    mutate(first = third)
)

#overlapping with DEG
preG_DEG <- femaleDEGanno_whole %>%
  filter(cluster == "2") %>%
  filter(eday == "E11" | eday == "E12")

preG_motif <- merge(test, preG_DEG, by.x = "first", by.y = "gene") # 38 unique motifs

MotifPlot(
  object = preG,
  assay = "peaksbyc",
  motifs = unique(preG_motif$motif)
)

#GEX dotplot
DotPlot(
  All_decont_sexed,
  assay = 'SCT',
  features = c(
    "Lhx9",
    "Msx1",
    "Emx2",
    "Gata6",
    "Trps1",
    "Rora",
    "Nr2f2",
    "Tcf7l1",
    "Tcf7l2",
    "Lef1",
```

```r
    "Meis1",
    "Meis2",
    "Nfia",
    "Nfib",
    "Tcf4",
    "Tcf12",
    "Rfx3",
    "Rfx7",
    "Prdm5",
    "Scrt2",
    "Runx1",
    "Pbx3",
    "Foxp1",
    "Cux1",
    "Pbx1",
    "Bnc2",
    "Bach2",
    "Nfat5",
    "Pknox2",
    "Esr1",
    "Sox5",
    "Maf",
    "Gli3",
    "Zbtb7c",
    "Klf3",
    "Klf8",
    "Klf12",
    "Klf7",
    "Sp3"
  )
), idents = c(
  "0_Male11.5",
  "0_Male12.5",
  "4_Male11.5",
  "4_Male12.5",
  "5_Male11.5",
  "5_Male12.5",
  "0_Female11.5",
  "0_Female12.5",
  "4_Female11.5",
  "4_Female12.5",
  "2_Female11.5",
  "2_Female12.5"
), cols = c("lightgrey", "#6A040F")
) +
  coord_flip()
```

Motif scan was run on all open peaks of the All_decont_sexed dataset, no need to repeat for female peaks. Extract pre-G enriched motifs and overlap with pre-G DAP regions

```r
#look for the bed files that are enriched pre-G motifs
list <- list.files("/yourpath/motif_sites/")
search <- unique(preG_motif$motif)
search <- gsub("\\.", "_", search)
```

```
matches_list <- as.list(lapply(
  search,
  grep,
  x = list,
  value = TRUE,
  ignore.case = TRUE
))

#extract pre-G peaks
preGDAP <- FemaleDAPbyCluster %>%
  filter(cluster == "5") %>%
  filter(eday == "E11" | eday == "E12")
preGDAP_ranges <- StringToGRanges(preGDAP$peak, sep = c("-", "-"))

#overlap pre-G motif bed files with pre-G peaks
for (file in matches_list) {
  granges = readBed(file, track.line = FALSE, remove.unusual = FALSE)
  result <- as.data.frame(findOverlaps(granges, preGDAP_ranges))
  hit <- sertoliDAP[result$subjectHits, ]
  csv <- read.delim(file, header = FALSE)
  motif <- csv[result$queryHits, ]
  write.csv(hit, file = paste0("/yourpath/overlaps/", gsub('.bed', '_linkedDAP.csv',
  file)))
  write.csv(motif, file = paste0("/yourpath/overlaps/", gsub('.bed', '_motif.csv',
  file)))
}
```

Identify linked motif targets

```
#in the folder where all output _linkedDAP files are saved
#identify positively linked gene (zscore>0)
matches_list = list.files(pattern = "linkedDAP.csv")
output <- data_frame()
for (file in matches_list) {
  csv <- read_csv(file)
  gene <- as.data.frame(t(as.data.frame(unique(csv[csv$zscore > 0, ]$gene)))) #t a
dataframe with one column makes it chr string
  output <- bind_rows(output, gene)
}
output <- as.data.frame(t(output))
colnames(output) <- matches_list
write.csv(output, "preGMotif_targetGene.csv")

#overlapping positively linked genes with sertoli DEG (this is done for both E11.5 and
E12.5 separately)
preGDEG12 <- femaleDEGanno_whole %>%
  filter(cluster == "2" & eday == "E12")

filter_target <- data.frame()
names <- names(preGMotif_targetGene)
for (col_name in names(preGMotif_targetGene)) {
  filter_row <- as.data.frame(t(
    filter(
      preGMotif_targetGene[col_name],
```

```r
      preGMotif_targetGene[[col_name]] %in% preGDEG12$gene
    )
  ))
  filter_target <- bind_rows(filter_target, filter_row)
}
filter_target <- as.data.frame(t(filter_target))
write.csv(filter_target, "preGMotif_targetGene_DEG.csv")

#repeat with negatively linked gene
output2 <- data_frame()
for (file in matches_list) {
  csv <- read_csv(file)
  gene <- as.data.frame(t(as.data.frame(unique(csv[csv$zscore < 0, ]$gene)))) #t a
dataframe with one column makes it chr string
  gene$file_name <- file
  output2 <- bind_rows(output2, gene)
}
output2 <- as.data.frame(t(output2))
colnames(output2) <- matches_list
write.csv(output2, "preGMotif_NEGATIVE_targetGene.csv")

#plotting heatmaps of linked GEX
#heatmap of linked gene
preG_pos <- unique(unlist(preGMotif_targetGene_DEG))
preG_neg <- unique(unlist(preGMotif_NEGATIVE_targetGene))
preG_all <- c(preG_pos, preG_neg)
preG_all <- preG_all[!is.na(preG_all)]

DefaultAssay(All_decont_sexed) <- 'SCT'
Idents(All_decont_sexed) <- "cluster.SexEday"
cells <- WhichCells(
  All_decont_sexed,
  ident = c("5_Male11.5", "5_Male12.5", "2_Female11.5", "2_Female12.5")
)

DoHeatmap(
  All_decont_sexed,
  features = preG_all,
  cells = cells,
  assay = "SCT",
  slot = "data",
  group.by = "ident",
  label = TRUE,
) + scale_fill_gradientn(colors = c("blue", "white", "red"))
```

Computing motif co-occurrence

```r
preG_freq <- as.data.frame(table(unlist(preGMotif_targetGene_DEG)))
name <- preG_freq$Var1
output <- data_frame()
for (gene in name) {
  motif <- as.data.frame(t(as.data.frame(
    colnames(preGMotif_targetGene_DEG[, grepl(paste0("\\b", gene, "\\b"),
    preGMotif_targetGene_DEG)])
```

```
  )))
  output <- bind_rows(output, motif)
}
output <- cbind(preG_freq, output)
write.csv(output, "preG_targetDEG_Freq.csv")

foo <- preG_targetDEG_Freq[, -2]
colnames(foo)[1] <- "Gene"
foo <- melt(setDT(foo), id.vars = "Gene", measure = patterns("^V"))[nchar(value) > 0 &
                                                                    complete.cases
                                                                    (value)]

mymat <- crossprod(table(foo[, c(1, 3)])) #create a matrix with crosspod
diag(mymat) <- 0 #change diagonal values to 0

#plot co-occurance as a heatmap
colnames(mymat) <- gsub("_sites_linkedDAP.csv", "", colnames(mymat))
rownames(mymat) <- gsub("_sites_linkedDAP.csv", "", rownames(mymat))
colnames(mymat) <- rownames(mymat)
palf <- colorRampPalette(c("lightgrey", "#6A040F"))
heatmap.2(
  mymat,
  col = palf(10),
  trace = "none",
  Rowv = FALSE,
  Colv = FALSE,
  margins = c(20, 20)
)
```

chromVAR motif activity

```
#plotting GEX
DefaultAssay(All_decont_sexed) <- 'chromvar'
Idents(All_decont_sexed) <- "cluster.SexEday"
DotPlot(
  All_decont_sexed,
  assay = 'chromvar',
  features = unique(preG_motif$motif),
  idents = c(
    "0_Male11.5",
    "0_Male12.5",
    "4_Male11.5",
    "4_Male12.5",
    "5_Male11.5",
    "5_Male12.5",
    "0_Female11.5",
    "0_Female12.5",
    "4_Female11.5",
    "4_Female12.5",
    "2_Female11.5",
    "2_Female12.5"
  )
  cols = c("lightgrey", "#6A040F")
)
```

```
+ coord_flip() + scale_x_discrete(limits = rev)

#plotting UMAP and chromVAR featureplots
DefaultAssay(supporting) <- 'chromvar'
FeaturePlot(
  supporting,
  reduction = "wnn.umap",
  keep.scale = "feature",
  features = c("MA0768.2", "MA0666.2", "MA0002.2", "MA0481.3"),
  cols = c("lightgrey", "#6A040F"),
  order = TRUE,
  min.cutoff = "q1",
  max.cutoff = "q99"
)
```

Comparing DAPs of E11.5 XX PSC b/t E11.5 and E12.5 pre-G

```
#E11.5 XX PSC vs E11.5 pre-G
#DEG
DefaultAssay(All_decont_sexed) <- "SCT"
Idents(All_decont_sexed) <- All_decont_sexed$cluster.SexEday
female_deg11 <- FindMarkers(All_decont_sexed, ident.1 = "4_Female11.5", ident.2 =
                              "2_Female11.5")

female_deg11_PSC <- female_deg11[which(female_deg11$p_val_adj < 0.05 &
                                         female_deg11$avg_log2FC > 0.25), ]
colnames(female_deg11_PSC)[1] <- "gene"
female_deg11_preG <- female_deg11[which(female_deg11$p_val_adj < 0.05 &
                                          female_deg11$avg_log2FC < -0.25), ]
colnames(female_deg11_preG)[1] <- "gene"
female_deg11_PSC$cluster <- "4"
female_deg11_preG$cluster <- "2"

#DAP annotation (Fig 5I didn't show annotation, only number)
DefaultAssay(All_decont_sexed) <- "peaksbyc"
Idents(All_decont_sexed) <- All_decont_sexed$cluster.SexEday
female_dap11 <- FindMarkers(
  All_decont_sexed,
  ident.1 = "4_Female11.5",
  ident.2 = "2_Female11.5",
  logfc.threshold = .1,
  min.pct = 0.01,
  test.use = 'LR',
  latent.vars = 'nCount_peaksbyc'
)

female_dap11_PSC <- female_dap11[which(female_dap11$p_val_adj < 0.05 &
                                         female_dap11$avg_log2FC > 0.25), ]
female_dap11_PSC$cluster <- "4"
female_dap11_preG <- female_dap11[which(female_dap11$p_val_adj < 0.05 &
                                          female_dap11$avg_log2FC < -0.25), ]
female_dap11_preG$cluster <- "2"
colnames(female_dap11_PSC)[1] <- "peak"
colnames(female_dap11_preG)[1] <- "peak"
```

```r
female_dap11_PSC_linked <- merge(female_dap11_PSC,
                                 linkspeak_whole,
                                 by = c("peak"),
                                 all.x = TRUE)
female_dap11_preG_linked <- merge(female_dap11_preG,
                                  linkspeak_whole,
                                  by = c("peak"),
                                  all.x = TRUE)

female_dap11_PSC_linked <- merge(
  female_dap11_PSC_linked,
  female_deg11_PSC,
  by = c("gene"),
  all.x = TRUE
)
female_dap11_preG_linked <- merge(
  female_dap11_preG_linked,
  female_deg11_preG,
  by = c("gene"),
  all.x = TRUE
)

female_dap11_PSC_linked$annotation <- with(female_dap11_PSC_linked,
                                           ifelse(is.na(gene), "non-linked", "linked"))
female_dap11_preG_linked$annotation <- with(female_dap11_preG_linked,
                                            ifelse(is.na(gene), "non-linked", "linked"))

female_dap11_PSC_anno <- female_dap11_PSC_linked %>%
  mutate(
    annotation = case_when(
      annotation == "linked" & is.na(p_val.y) ~ "linked:noDEG",
      annotation == "linked" &
        !is.na(p_val.y) ~ "linked:DEG",
      annotation == "non-linked" ~ "nolink"
    )
  )

female_dap11_preG_anno <- female_dap11_preG_linked %>%
  mutate(
    annotation = case_when(
      annotation == "linked" & is.na(p_val.y) ~ "linked:noDEG",
      annotation == "linked" &
        !is.na(p_val.y) ~ "linked:DEG",
      annotation == "non-linked" ~ "nolink"
    )
  )

combine <- rbind(female_dap11_PSC_anno, female_dap11_preG_anno)
annotation <- combine %>%
  group_by(cluster.x, annotation) %>%
  summarise(count = n_distinct(peak))

annotation <- melt(dcast(annotation, annotation ~ cluster.x))
```

```r
annotation[is.na(annotation)] = 0

ggplot(annotation, aes(
  fill = factor(annotation, levels = c("linked:DEG", "linked:noDEG", "nolink")),
  y = count,
  x = cluster.x
)) +
  geom_bar(position = "stack", stat = "identity") +
  scale_fill_manual(name = "Linkage",
                    values = c("#edae49", "#00798c", "#8d96a3")) +
  theme(
    panel.grid.major = element_blank(),
    panel.grid.minor = element_blank(),
    panel.background = element_blank(),
    axis.line = element_line(colour = "black")
  ) +
  ylim(0, 10)

#E11.5 XX PSC vs E12.5 pre-G
#DEG
DefaultAssay(All_decont_sexed) <- "SCT"
Idents(All_decont_sexed) <- All_decont_sexed$cluster.SexEday
female_deg12 <- FindMarkers(All_decont_sexed, ident.1 = "4_Female11.5", ident.2 =
                              "2_Female12.5")

female_deg12_PSC <- female_deg12[which(female_deg12$p_val_adj < 0.05 &
                                         female_deg12$avg_log2FC > 0.25), ]
female_deg12_PSC$gene <- rownames(female_deg12_PSC)
female_deg12_preG <- female_deg12[which(female_deg12$p_val_adj < 0.05 &
                                          female_deg12$avg_log2FC < -0.25), ]
female_deg12_preG$gene <- rownames(female_deg12_preG)
female_deg12_PSC$cluster <- "4"
female_deg12_preG$cluster <- "2"

#DAP
DefaultAssay(All_decont_sexed) <- "peaksbyc"
Idents(All_decont_sexed) <- All_decont_sexed$cluster.SexEday
female_dap12 <- FindMarkers(
  All_decont_sexed,
  ident.1 = "4_Female11.5",
  ident.2 = "2_Female12.5",
  logfc.threshold = .1,
  min.pct = 0.01,
  test.use = 'LR',
  latent.vars = 'nCount_peaksbyc'
)

female_dap12_PSC <- female_dap12[which(female_dap12$p_val_adj < 0.05 &
                                         female_dap12$avg_log2FC > 0.25), ]
female_dap12_PSC$cluster <- "4"
female_dap12_preG <- female_dap12[which(female_dap12$p_val_adj < 0.05 &
                                          female_dap12$avg_log2FC < -0.25), ]
female_dap12_preG$cluster <- "2"
colnames(female_dap12_PSC)[1] <- "peak"
```

```r
colnames(female_dap12_preG)[1] <- "peak"

female_dap12_PSC_linked <- merge(female_dap12_PSC,
                                 linkspeak_whole,
                                 by = c("peak"),
                                 all.x = TRUE)
female_dap12_preG_linked <- merge(female_dap12_preG,
                                  linkspeak_whole,
                                  by = c("peak"),
                                  all.x = TRUE)

female_dap12_PSC_linked <- merge(
  female_dap12_PSC_linked,
  female_deg12_PSC,
  by = c("gene"),
  all.x = TRUE
)
female_dap12_preG_linked <- merge(
  female_dap12_preG_linked,
  female_deg12_preG,
  by = c("gene"),
  all.x = TRUE
)

female_dap12_PSC_linked$annotation <- with(female_dap_PSC_linked, ifelse(is.na(gene),
"non-linked", "linked"))
female_dap12_preG_linked$annotation <- with(female_dap_preG_linked,
                                            ifelse(is.na(gene), "non-linked", "linked"))

female_dap12_PSC_anno <- female_dap12_PSC_linked %>%
  mutate(
    annotation = case_when(
      annotation == "linked" & is.na(p_val.y) ~ "linked:noDEG",
      annotation == "linked" &
        !is.na(p_val.y) ~ "linked:DEG",
      annotation == "non-linked" ~ "nolink"
    )
  )

female_dap12_preG_anno <- female_dap12_preG_linked %>%
  mutate(
    annotation = case_when(
      annotation == "linked" & is.na(p_val.y) ~ "linked:noDEG",
      annotation == "linked" &
        !is.na(p_val.y) ~ "linked:DEG",
      annotation == "non-linked" ~ "nolink"
    )
  )

combine <- rbind(female_dap12_PSC_anno, female_dap12_preG_anno)
annotation <- combine %>%
  group_by(cluster.x, annotation) %>%
  summarise(count = n_distinct(peak))
```

```
annotation <- melt(dcast(annotation, annotation ~ cluster.x))
annotation[is.na(annotation)] = 0

ggplot(annotation, aes(
  fill = factor(annotation, levels = c("linked:DEG", "linked:noDEG", "nolink")),
  y = value,
  x = variable
)) +
  geom_bar(position = "stack", stat = "identity") +
  scale_fill_manual(name = "Linkage",
                    values = c("#edae49", "#00798c", "#8d96a3")) +
  theme(
    panel.grid.major = element_blank(),
    panel.grid.minor = element_blank(),
    panel.background = element_blank(),
    axis.line = element_line(colour = "black")
  ) +
  ylim(0, 4000)
```

Figure 6- Lef1 and Msx1 coverage plots

```
Idents(All_decont_sexed) <- "cluster.SexEday"
DefaultAssay(All_decont_sexed) <- "peaksbyc"
original_levels <- levels(Idents(All_decont_sexed))
order <- c(
  "0_Female11.5",
  "0_Female12.5",
  "0_Female13.5",
  "4_Female11.5",
  "4_Female12.5",
  "4_Female13.5",
  "2_Female11.5",
  "2_Female12.5",
  "2_Female13.5"
)
male_order <- c(
  "0_Male11.5",
  "0_Male12.5",
  "0_Male13.5",
  "4_Male11.5",
  "4_Male12.5",
  "4_Male13.5",
  "5_Male11.5",
  "5_Male12.5",
  "5_Male13.5"
)
new_levels <- c(order, male_order, setdiff(original_levels, c(order, male_order)))
Idents(All_decont_sexed) <- factor(Idents(All_decont_sexed), levels = new_levels)

plot_idents <- factor(
  c(
    "0_Female11.5",
```

```
    "0_Female12.5",
    "0_Female13.5",
    "4_Female11.5",
    "4_Female12.5",
    "4_Female13.5",
    "2_Female11.5",
    "2_Female12.5",
    "2_Female13.5"
  ),
  levels = c(
    "0_Female11.5",
    "0_Female12.5",
    "0_Female13.5",
    "4_Female11.5",
    "4_Female12.5",
    "4_Female13.5",
    "2_Female11.5",
    "2_Female12.5",
    "2_Female13.5"
  )
)

#Msx1
CoveragePlot(
  object = All_decont_sexed,
  region = "Msx1",
  features = "Msx1",
  expression.assay = "SCT",
  idents = order,
  extend.upstream = 500,
  extend.downstream = 10000,
  ymax = 50
)
CoveragePlot(
  object = All_decont_sexed,
  region = c("chr5-37975000-37982000"),
  features = "Msx1",
  expression.assay = "SCT",
  idents = order,
  ymax = 50
)
CoveragePlot(
  object = All_decont_sexed,
  region = "Msx1",
  features = "Msx1",
  expression.assay = "SCT",
  idents = male_order,
  extend.upstream = 500,
  extend.downstream = 10000,
  ymax = 50
)
CoveragePlot(
  object = All_decont_sexed,
  region = c("chr5-37975000-37982000"),
```

```
  features = "Msx1",
  expression.assay = "SCT",
  idents = male_order,
  ymax = 50
)

#Lef1
CoveragePlot(
  object = All_decont_sexed,
  region =  c("chr3-131100000-131120000"),
  features = "Lef1",
  expression.assay = "SCT",
  idents = order,
  ymax = 50
)
CoveragePlot(
  object = All_decont_sexed,
  region =  c("chr3-131210000-131230000"),
  features = "Lef1",
  expression.assay = "SCT",
  idents = order,
  ymax = 50
)
CoveragePlot(
  object = All_decont_sexed,
  region =  c("chr3-131100000-131120000"),
  features = "Lef1",
  expression.assay = "SCT",
  idents = male_order,
  ymax = 50
)
CoveragePlot(
  object = All_decont_sexed,
  region =  c("chr3-131210000-131230000"),
  features = "Lef1",
  expression.assay = "SCT",
  idents = male_order,
  ymax = 50
)

#Klf7
CoveragePlot(
  object = All_decont_sexed,
  region = "Klf7",
  features = "Klf7",
  expression.assay = "SCT",
  idents = c("2_Female11.5", "5_Male11.5"),
  extend.upstream = 150000,
  extend.downstream = 5000
)

CoveragePlot(
  object = All_decont_sexed,
  region = c("chr1-64110000-64128000"),
```

```r
  features = "Klf7",
  expression.assay = "SCT",
  idents = c("2_Female11.5", "5_Male11.5")
)

#Tcf4
CoveragePlot(
  object = All_decont_sexed,
  region = c("chr18-69342500-69350000"),
  features = "Tcf4",
  expression.assay = "SCT",
  idents = c("2_Female11.5", "5_Male11.5"),
  ymax = 30
)
CoveragePlot(
  object = All_decont_sexed,
  region = c("chr18-69775000-69785000"),
  features = "Tcf4",
  expression.assay = "SCT",
  idents = c("2_Female11.5", "5_Male11.5"),
  ymax = 30
)

#Foxl2
CoveragePlot(
  object = All_decont_sexed,
  region = "Foxl2",
  features = "Foxl2",
  expression.assay = "SCT",
  idents = c("2_Female11.5", "2_Female12.5", "5_Male11.5", "5_Male12.5"),
  extend.upstream = 350000,
  extend.downstream = 1000
)
CoveragePlot(
  object = All_decont_sexed,
  region = c("chr9-98610000-98625000"),
  features = "Foxl2",
  expression.assay = "SCT",
  idents = c("2_Female11.5", "2_Female12.5", "5_Male11.5", "5_Male12.5"),
  ymax = 17
)
CoveragePlot(
  object = All_decont_sexed,
  region = c("chr9-98650000-98670000"),
  features = "Foxl2",
  expression.assay = "SCT",
  idents = c("2_Female11.5", "2_Female12.5", "5_Male11.5", "5_Male12.5"),
  ymax = 17
)
CoveragePlot(
  object = All_decont_sexed,
  region = c("chr9-98950000-98960000"),
  features = "Foxl2",
  expression.assay = "SCT",
```

```
  idents = c("2_Female11.5", "2_Female12.5", "5_Male11.5", "5_Male12.5"),
  ymax = 17
)
```
